# Analysis and Comparison of the Laboratory and Ultrasonographic Findings in Cattle with Various Types of Small and Large Intestinal Ileus

**DOI:** 10.3390/ani16182850

**Published:** 2026-09-10

**Authors:** Ueli Braun, Karl Nuss, Christian Gerspach

**Affiliations:** Department of Farm Animals, Vetsuisse Faculty, University of Zurich, 8057 Zurich, Switzerland

**Keywords:** cattle, small intestine, large intestine, ileus, laboratory findings, ultrasonographic findings

## Abstract

**Simple Summary:**

Ileus is the interruption of the passage of intestinal contents. Ileus can be either functional (paralytic) or mechanical; the latter is most common in cattle and can be caused by caecal dilatation, compression, haemorrhagic bowel syndrome, intussusception, incarceration, intussusception, mesenteric torsion, strangulation, torsion of the spiral colon, or volvulus. The clinical, laboratory, and ultrasonographic findings as well as the treatment and the course of disease have been described by the authors in numerous publications. The various types of ileus and numerous scientific publications on this topic make it difficult for veterinarians to recognize clinical signs and navigate the large volume of information. Therefore, this study aimed to analyse and compare the laboratory and ultrasonographic findings in 1163 cows with one of eleven types of small and large intestinal ileus. The results will hopefully allow veterinarians to assign laboratory and ultrasonographic findings to a specific type of ileus and improve diagnostic accuracy. The latter is imperative because the prognosis for each type of ileus differs greatly.

**Abstract:**

The laboratory and ultrasonographic findings in 1163 cattle with one of 11 types of ileus, including caecal dilatation, compression, haemorrhagic bowel syndrome, mesenteric torsion, paralytic ileus, incarceration, intussusception, obstruction, strangulation, volvulus, and torsion of the spiral colon were compared. The prevalences of the individual laboratory and ultrasonographic findings varied greatly among the different types of ileus; hypochloraemia ranged from 98.4 to 12.4%, hypokalaemia from 98.4 to 42.4%, azotaemia from 91.9 to 38.3%, acidosis from 47.8 to 3.3%, and haemoconcentration from 71.7 to 23.2%. Ultrasonographically, dilated small intestine varied from 100 to 43.3%, intestinal atony from 98.4 to 0%, free abdominal fluid from 66.7 to 16.0%, abomasal dilatation from 44.4 to 0%, and empty poststenotic small intestine from 34.9 to 0%. The cause of the ileus was detected in only 12.0% of the cattle examined. Ultrasonography is ideally suited for the visualisation of typical signs characteristic of ileus (intestinal dilatation and intestinal atony) but less so for determining the cause. The differences among the variables can be explained primarily by the type of ileus, the duration of illness, and the localisation of the intestinal ileus.

## 1. Introduction

Recently, we compared the clinical findings in 1163 cattle with various types of small and large intestinal ileus [1]. The present report analyses the laboratory and ultrasonographic findings of the same cattle. The explanations and information were derived from the publications previously used for the following: (in alphabetical order) caecal dilatation [2], compression of the small intestine [3], haemorrhagic bowel syndrome [4,5], mesenteric torsion [6], paralytic ileus [7], small intestinal incarceration [8], small intestinal intussusception [9], small intestinal obstruction [10], small intestinal strangulation [11], small intestinal volvulus [12], and torsion of the spiral colon [13]. In addition, the ultrasonographic findings in 30 cows with caecal dilatation were used [14]. Understanding from previous work on ileum impaction [15], internal herniation [16], strangulation of the duodenum by the uterus during late pregnancy [17], and herniation of the gravid uterus through a mesoduodenal defect and concurrent omental hernia [18] were also incorporated into the manuscript.

## 2. Brief Overview of the Laboratory and Ultrasonographic Findings in Cattle with Ileus

### 2.1. Laboratory Findings

Cattle with ileus undergo disruption of the fluid, electrolyte, and acid–base balance [19], depending primarily on the type and location of the ileus and the duration of the illness. The principal blood changes are haemoconcentration, hypochloraemia, hypokalaemia, hyponatraemia, and azotaemia [20], and they are grouped under the terms *abomasal reflux syndrome* and *hypokalaemic metabolic alkalosis*. However, shock and pain may also result in haemoconcentration and azotaemia, and localised ischaemic events, including vascular constriction in a torsed intestinal loop, lead to the production of lactate, causing metabolic acidosis. Abnormalities caused by abomasal reflux syndrome occur when chloride-containing abomasal content is refluxed into the rumen because of a delayed or impaired transit of the ingesta in the abomasum or duodenum. In general, hypochloraemic hypokalaemic metabolic alkalosis is more pronounced when the location of the passage disruption is proximal or when the passage of the ingesta is completely blocked [20]. In a study involving experimental ligation of the proximal, middle, or distal parts of the small intestine, abomasal reflux syndrome was more pronounced with ligation of the duodenum than with ligation of the jejunum or ileum [21]. Hypochloraemic hypokalaemic metabolic alkalosis developed quickly after or compression by adhesions at the duodenal sigmoid flexure [22].

Haemoconcentration and azotaemia are often mild initially and only become severe after about 3 days [23]. Cows with duodenal sigmoid flexure volvulus also had severe hyperbilirubinaemia and a marked increase in the activity of the liver enzymes γ-glutamyltransferase (γ-GT) and glutamate dehydrogenase (GLDH) [24]; these changes occur with mechanical obstruction of the bile duct, resulting in backflow of bile into the liver. Active local peritonitis may be accompanied by leucocytosis with a left shift [25]. Eleven cows with adhesions at the duodenal sigmoid flexure had neutrophilia and increased plasma fibrinogen concentrations [22].

### 2.2. Ultrasonographic Findings

When ileus of the small intestine is suspected, an ultrasonographic examination should evaluate the diameter, motility, and anatomic arrangement of the small intestine, evidence of peritonitis, and the possible cause [26]. The most important variables are the diameter and motility of the small intestine; identification of the cause of ileus via ultrasonography is rarely possible. In cattle with ileus, the small intestine is dilated in at least one area, with a diameter greater than 3.5 cm. The loops become more dilated with ileus of the proximal small intestine [26]. The largest diameter of intestine measured from the 12th intercostal space in cattle varied from 6.5 to 9.9 cm with ileus of the duodenum, from 3.5 to 9.8 cm with ileus of the jejunum, and from 4.4 to 5.5 cm with ileus of the ileum [26]. When interpreting intestinal diameter, it is important to remember that in healthy cows with a full intestinal tract, all parts of the intestine have a similar diameter. By contrast, in animals with ileus, in addition to the extremely dilated loops of intestine proximal to the ileus, empty loops of intestine are usually present distal to the ileus. The intestinal dia-meter in a healthy cow is constantly changing, whereas the intestinal diameter in a cow with ileus remains increased because intestinal motility is markedly reduced or absent [26]. However, intestinal contents often move without intestinal contractions. The flow of intestinal contents is presumably due to passive intestinal movement by respiratory activity and possibly by the movement of adjacent organs such as the rumen or abomasum. Sometimes, hypoechogenic fluid attributable to transudation is visible between the dilated loops of intestine.

The anatomic location of ileus markedly affects the number of dilated loops of intestine seen in cross-sections and longitudinally from either the flank or each intercostal space [26]. When only one or a few loops of intestine, usually markedly dilated, are seen, ileus of the duodenum is most likely [26,27]. More than five loops of small intestine seen in cross-sections in one area usually indicates ileus of the jejunum or ileum.

The contents of the small intestine appear predominantly echogenic and rarely hypoechogenic [26]. Different parts of the intestine in the same animal may be hyperechogenic or hypoechogenic. Intraluminal gas, which is associated with reverberation artifacts, is rarely observed.

The cause of ileus can seldom be determined ultrasonographically [26]. Often, this is because the cause of ileus is further from the abdominal wall than the penetration capacity of the transducer. Diagnosis is also difficult in cows in advanced pregnancy [28]. A common cause of ileus is intussusception, the ultrasonographic appearance of which in a cross-section has been described as *bowel within bowel*, *bull’s eye lesion*, *target pattern*, or as a multiple-layered, onion-ring-type mass with varying echogenicities [26]. Viewed longitudinally, the typical bowel-within-bowel pattern is clearly identifiable and has been described as a “sandwich” configuration. The intussusception was visualised in 5.7% of 123 cattle [9], 60.0% of 5 cattle [29], and 66.7% of 6 cattle [28] and appeared as the typical *bowel-within-bowel* pattern. An intraluminal mass indicates an ileus attributable to small intestinal obstruction except for haemorrhagic bowel syndrome. In cattle with small intestinal obstruction, the actual obstruction was seen as a focal, hyperechogenic intraluminal mass obliterating the intestinal lumen in 3 (3.4%) out of 89 cattle [10], and in cattle with haemorrhagic bowel syndrome, localised hyperechogenic masses consistent with blood clots were seen in the lumen of the small intestine in 4 (33.3%) out of 12 cattle [30] and in 12 (19.0%) out of 63 cattle [5]. In rare cases, compression of the small intestine by abscesses in the region of the liver or compression of the duodenum between the liver and gallbladder can be identified. Compression caused by a multi-chambered abdominal abscess with adhesions involving the jejunum could be seen in another cow [3]. In an 11-month-old Holstein heifer, ultrasonography showed compression of the duodenum by a displaced gallbladder [31]. Ultrasonographic visualisation of the site of compression has also been reported in cows with fat necrosis [32]. In one cow, omental fat necrosis detected near the right abdominal wall, medial to the liver, appeared as hyperechogenic structures that impaired visibility of the intestinal loops [33]. Compression can also be caused by generalised peritonitis with fibrinous adhesions involving the small intestine. In such cases, thickening of the intestinal wall, fibrinous deposits, and accumulation of intra-abdominal fluid are usually seen. In cattle with internal hernia, the cause of the ileus could not be identified [8,16]; the same was true for cattle with constipation involving the ileum [15], strangulation [11], mesenteric torsion [6], and volvulus [12].

Abomasal dilatation can result from retrograde accumulation of ingesta secondary to intestinal ileus. Depending on the degree of fill, the dilated abomasum can be imaged on the right side or even from the ventral abdomen caudal to the ribs. The abomasum is filled with hypoechogenic material, which frequently has a fluid consistency due to hypochloric acid sequestration. This often allows for good visualisation of the abomasal folds, which appear as thin, echogenic, and wavy structures within the ingesta. In contrast to left or right abomasal displacement, gas does not accumulate in the non-displaced, dilated abomasum. Occasionally, ingesta accumulates in the omasum and rumen; the resulting dilatation of the omasum facilitates visualisation of the omasal leaves. Accumulation of ingesta in the rumen also leads to dilatation and extension of the organ close to the right abdominal wall. This can be associated with noticeable compression of the liver.

Incomplete perforation of the intestine usually leads to chronic peritonitis [26]; ultrasonography reveals intra-abdominal fluid with fibrin bands that may have a spiderweb-type appearance among the loops of intestine and organs. Free intra-abdominal gas with its associated reverberation artifacts may be seen if intestinal gas leaks through the perforation [26].

## 3. Materials and Methods

### 3.1. Animals

The present study compares the laboratory and ultrasonographic findings in cattle with different types of ileus. The information was obtained from the following publications (in alphabetical order) and the raw data (hospital records) involving 1163 cattle with ileus at the Department of Farm Animals, University of Zurich, Switzerland, from 1985 to 2019:

- Caecal dilatation, n = 461 [2], for the laboratory variables, and [14], n = 30, for the ultrasonographic variables;
- Haemorrhagic bowel syndrome, n = 63 [4,5], referred to as *HBS* in the paper;
- Mesenteric torsion, n = 61 [6];
- Paralytic ileus, n = 57 [7];
- Small intestinal compression, n = 35 [3], referred to as *compression* in the paper;
- Small intestinal incarceration, n = 85 [8], referred to as *incarceration* in the paper;
- Small intestinal intussusception, n = 126 [9], referred to as *intussusception* in the paper;
- Small intestinal obstruction, n = 110 [10], referred to as *obstruction* in the paper;
- Small intestinal strangulation, n = 60 [11], referred to as *strangulation* in the paper;
- Small intestinal volvulus, n = 47 [12], referred to as *volvulus* in the paper;
- Torsion of the spiral colon, n = 58 [13].

### 3.2. Definitions Used

- *Caecal dilatation:* Dilatation of the caecum, accompanied by displacement, torsion, or retroflexion of the organ and sometimes distension of the spiral colon.
- *Haemorrhagic bowel syndrome:* Segmental necrohaemorrhagic inflammation of the small intestine, in which large intraluminal blood clots and sloughed mucosa result in mechanical obstruction of the jejunum.
- *Mesenteric torsion:* Intestines twisted around the mesenteric root, and depending on the site of the torsion, the ileum, caecum, or ascending colon is involved.
- *Paralytic ileus:* Impaired intestinal motor activity, clinically mimicking mechanical disruption of the ingesta transit in the absence of mechanical obstruction or compression.
- *Small intestinal compression:* Ileus caused by compression of the small intestine by nearby space-occupying lesions or other abdominal organs.
- *Small intestinal incarceration:* Ileus caused by the entrapment of parts of the small intestine through congenital or acquired openings in the mesentery, omentum, mesometrium, or remnants of embryonic structures (internal herniation) or in the abdominal wall (external herniation).
- *Small intestinal intussusception:* Ileus caused by an oral intestinal segment sliding into an aboral segment in a telescope-like fashion, thus blocking passage of ingesta.
- *Small intestinal obstruction:* Ileus caused by intraluminal obstruction of the small intestine by bezoars and other materials.
- *Small intestinal strangulation: Constriction* of the intestine by tissue bands of various origins that extend between two abdominal organs or between an organ and the abdominal wall or are freely floating in the abdominal cavity, causing interruption of the passage of intestinal contents.
- *Small intestinal volvulus:* Simple or multiple rotation(s) of a small intestinal segment around its mesenteric axis.
- *Torsion of the spiral colon:* Twisting of the spiral colon around its mesentery.

### 3.3. Laboratory Analyses

The laboratory analyses were described in numerous publications [6,7,8,9,10,11,12,13]. The analysis was limited to variables that were elevated or decreased relative to the reference interval in more than 40% of the cattle. These included haematocrit, total white blood cell count, the concentrations of total protein, urea, calcium, magnesium, inorganic phosphate, potassium, chloride, and base excess. The blood pH and rumen chloride concentration were included in the analysis, even though the blood pH was decreased in only 15.2% (150/988) and increased in 18.8% (186/988) of the cattle, and the rumen chloride concentration was increased in only 16.3% (157/963) of the cattle; these 2 variables are of major importance in cattle with ileus. Variables not included in the analysis were the concentrations of fibrinogen (>7 g/L in 24.6%), bilirubin (>6.5 μmol/L in 29.9%), γ-GT (>30 U/L in 15.4%), ASAT (>103 U/L in 30.3%), and bicarbonate (<20 mmol/L in 8.7% and >30 mmol/L in 22.7%). The pCO_2_ was not analysed even though it exceeded 45 mmHg in 41.3% of the cattle; instead, blood pH and base excess were evaluated.

### 3.4. Ultrasonographic Examinations

The abdomen was scanned from the right side as described [26]. Briefly, the area from the *tuber coxae* to the 8th intercostal space on the right side was examined using a 3.5 to 6.5 MHz convex transducer after clipping the hair and applying contact gel to the transducer and alcohol to the clipped skin. The appearance of loops of small intestine and their diameter, contents, and motility were assessed. In cattle with abdominal hernia, the region over the hernia was also evaluated ultrasonographically. In addition, the appearance, position, and nature of the contents of the caecum and proximal and *spiral ansa* of the colon and the presence of caecal dilatation were noted.

Characteristic signs of ileus of the small intestine are dilated loops of small intestine with a diameter ≥ 4.0 cm and no or very little intestinal motility. Caecal dilatation can always be imaged from the lateral abdominal wall. The dilated caecum and the proximal loop of the colon are almost always immediately adjacent to the abdominal wall. Only the wall of the dilated caecum and proximal loop of the colon closest to the transducer are seen ultrasonographically because of gas and appear as thick, echogenic, and semi-circular lines. In cases with fluid ingesta rather than gas in the caecum and proximal colon, the lumen appears moderately echogenic. Differentiation of the caecum and proximal loop of the colon may be difficult ultrasonographically unless the ileocaecal fold of the peritoneum between the two can be identified. Abomasal dilatation was diagnosed when the abomasum appeared dilated and filled with hypoechogenic ingesta and fluid. In cattle with abomasal dilatation, the abomasal folds appear as thin, echogenic, and wavy structures. Fibrin appears as irregularly shaped echogenic masses or strands between the intestinal loops.

### 3.5. Statistics

The statistical analysis was conducted using commercially available software (SPSS Statistics 26.0, IBM Corp., 2017). Data are reported as numbers, means, 95% CIs for means, medians, and percentages.

The means of the laboratory variables for the 11 types of ileus were compared using ANOVA, and the prevalences (proportions) of variables outside the reference intervals were determined using the chi-square test. A value of *p* < 0.05 was considered significant. When the *p*-value was <0.05, a post-hoc test (Bonferroni correction) was used to identify groups that differed significantly. Pearson correlation coefficients were calculated for various blood variables. The frequencies of abnormal ultrasonographic findings in the various types of ileus were compared using the chi-square test, and the Bonferroni correction was applied as described for the laboratory variables.

## 4. Laboratory Findings

### 4.1. Overview

**Figure 1:**
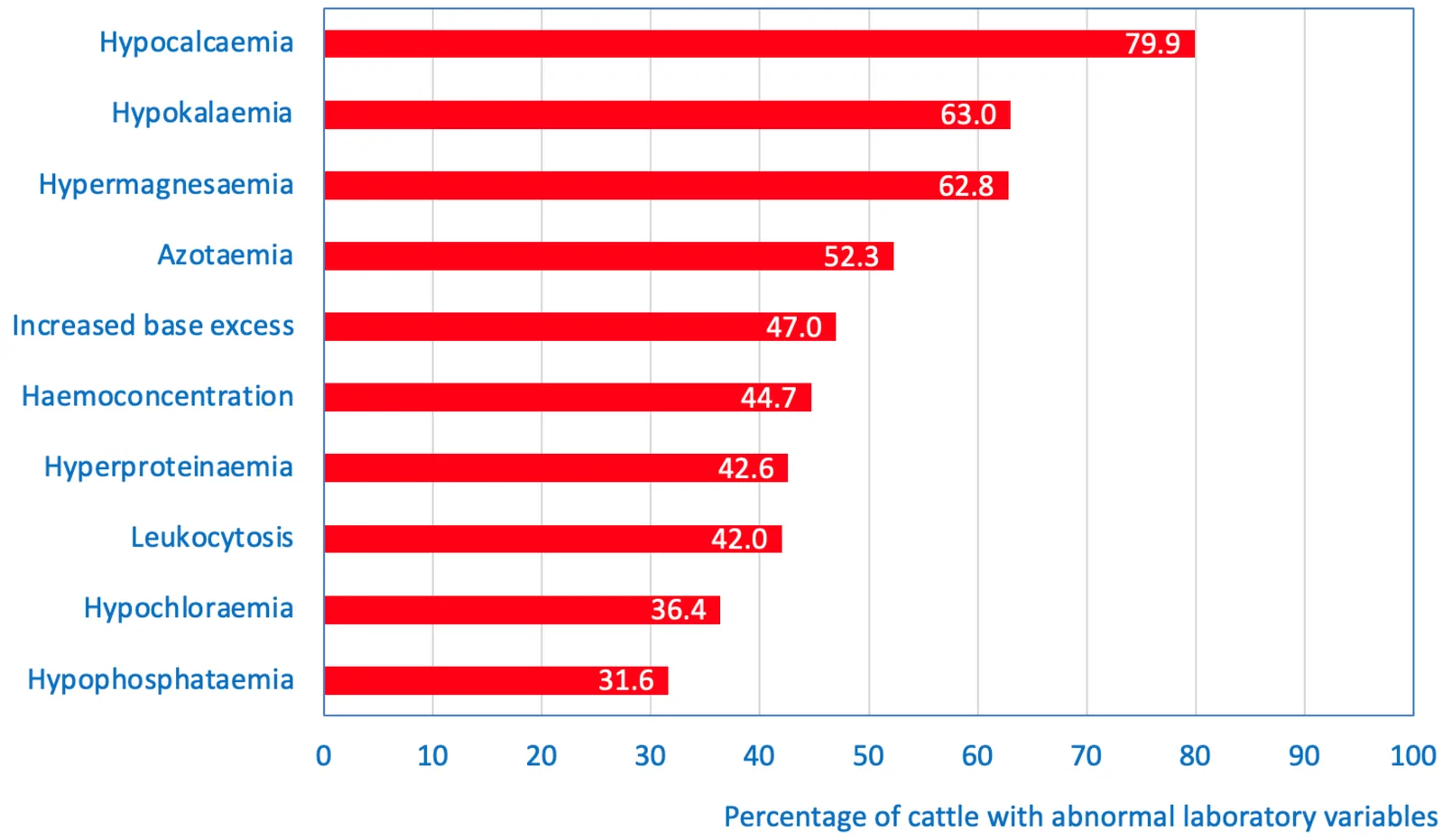
The most common abnormal blood variables in 1163 cattle with caecal dilatation (n = 461), compression (n = 35), HBS (n = 63), incarceration (n = 85), intussusception (n = 126), mesenteric torsion (n = 61), obstruction (n = 110), paralytic ileus (n = 57), strangulation (n = 60), torsion of the spiral colon (n = 58), and volvulus (n = 47).

The means and 95% CIs for the laboratory variables in each type of ileus are shown in Table S1 and the frequencies of values outside of the reference intervals in Table S2. The statistical comparisons of the means using ANOVA and those of the prevalences using the chi-square test and Bonferroni correction are shown in Tables S3–S14. The main abnormalities were hypocalcaemia (79.9%, 604/756, based on a calcium concentration < 2.30 mmol/L), hypokalaemia (63.0%, 715/1135, based on a potassium concentration < 4.00 mmol/L), hypermagnesaemia (62.8%, 471/750, based on a magnesium concentration > 1.00 mmol/L), azotaemia (52.3%, 593/1137, based on a urea concentration > 6.5 mmol/L), increased base excess (47.0%, 464/987, based on a base excess > 2.00 mmol/L), haemoconcentration (44.7%, 510/1141, based on haematocrit > 35%), leukocytosis (42.0%, 476/1132, based on a total white blood cell count > 10,000/μL), hyperproteinaemia (42.6%, 483/1135, based on a total protein concentration > 80 g/L), hypochloraemia (36.4%, 413/1135, based on a chloride concentration < 95 mmol/L), and hypophosphataemia (31.6%, 237/750, based on an inorganic phosphate concentration < 1.30 mmol/L) (Figure 1, Table S2). The blood pH was decreased in 15.2% (<7.35; 150/988) and increased in 18.8% (>7.45; 820/988), and the rumen chloride concentration was increased (>30.0 mmol/L) in 16.2% (157/973) of cases.

### 4.2. Haematocrit and Haemoconcentration

**Figure 2:**
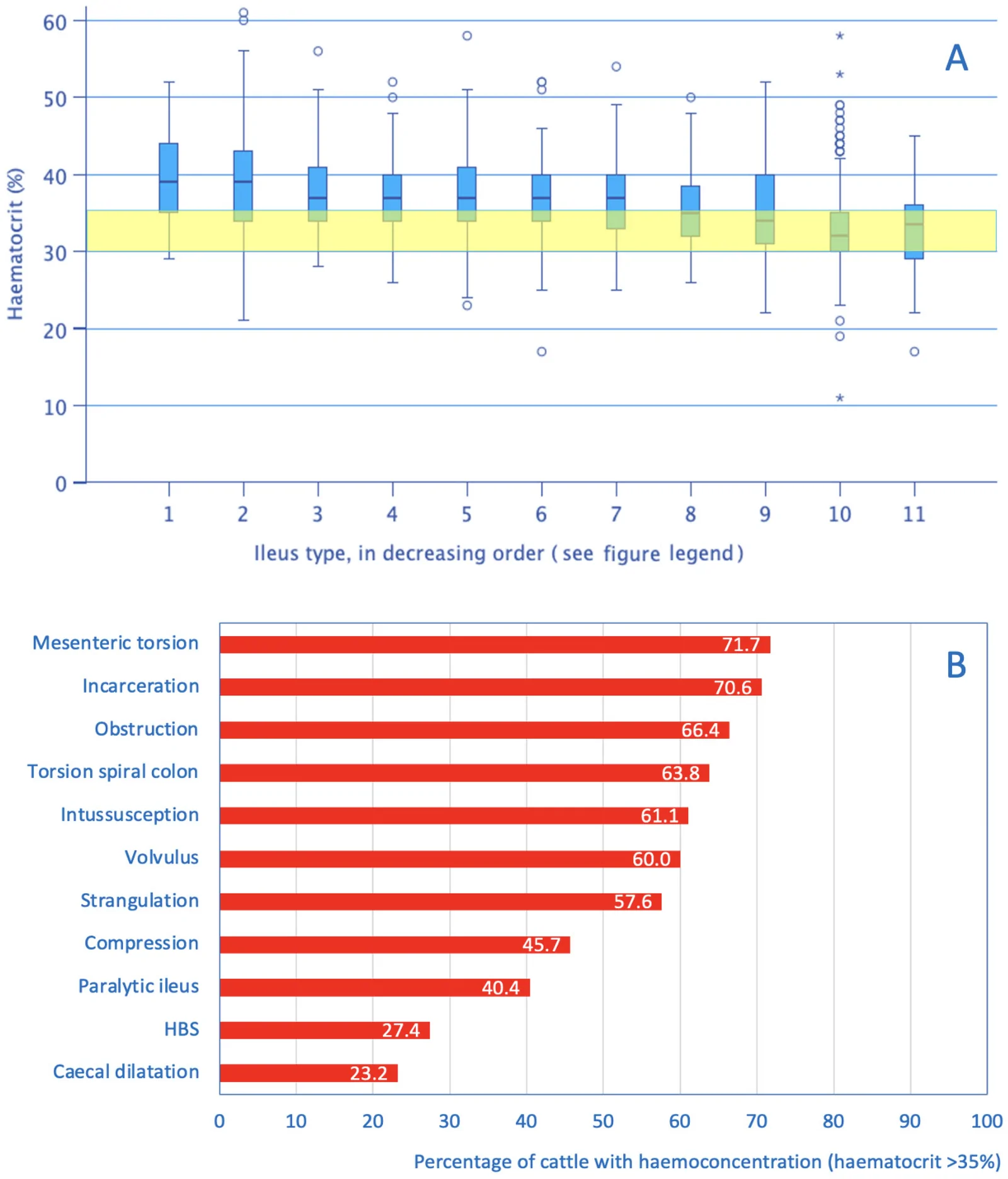
(**A**) Haematocrit of cattle with different types of ileus, in descending order (1 mesenteric torsion, 2 incarceration, 3 torsion of the spiral colon, 4 obstruction, 5 intussusception, 6 volvulus, 7 strangulation, 8 compression, 9 paralytic ileus, 10 caecal dilatation, and 11 HBS). The yellow area marks the reference interval (30 to 35%) for the haematocrit. Boxplot presentation of the numeric variables was completed as described [34]: Within the box, the thick horizontal line shows the median. The top and bottom of the blue box represent the upper and lower quartiles, respectively. The distance between the top of the box and the top of the whisker shows the range of the top 25% of scores. Similarly, the distance between the bottom of the box and the end of the bottom whisker shows the range of the lowest 25% of scores. ° Outlier; * extreme score. (**B**) Prevalence of haemoconcentration (defined as a haematocrit > 35%) in 1141 cattle with different types of ileus.

The median haematocrit was 35%, varying from 39% (mesenteric torsion, #1 in Figure 2A) to 34% (HBS, #11). The haematocrit exceeded the reference interval in cows with mesenteric torsion (#1), incarceration (#2), torsion of the spiral colon (#3), obstruction (#4), intussusception (#5), volvulus (#6), and strangulation (#7) and was within the reference interval (30 to 35%) in the remaining types (#8 to 11). The haematocrit in cows with mesenteric torsion (#1), incarceration (#2), torsion of the spiral colon (#3), obstruction (#4), intussusception (#5), volvulus (#6), and strangulation (#7) was significantly higher than in cows with caecal dilatation (#10) and HBS (#11) (*p* < 0.01; for further details and other significant differences, see Table S3.1).

The prevalence of haemoconcentration varied from 71.7% in cattle with mesenteric torsion to 23.2% in cattle with caecal dilatation (Figure 2B). Haemoconcentration occurred significantly more often in cattle with mesenteric torsion and incarceration than in cattle with paralytic ileus, HBS, or caecal dilatation (*p* < 0.05; for details and other significant differences, see Table S3.2).

### 4.3. Leukocytes and Leukocytosis

**Figure 3:**
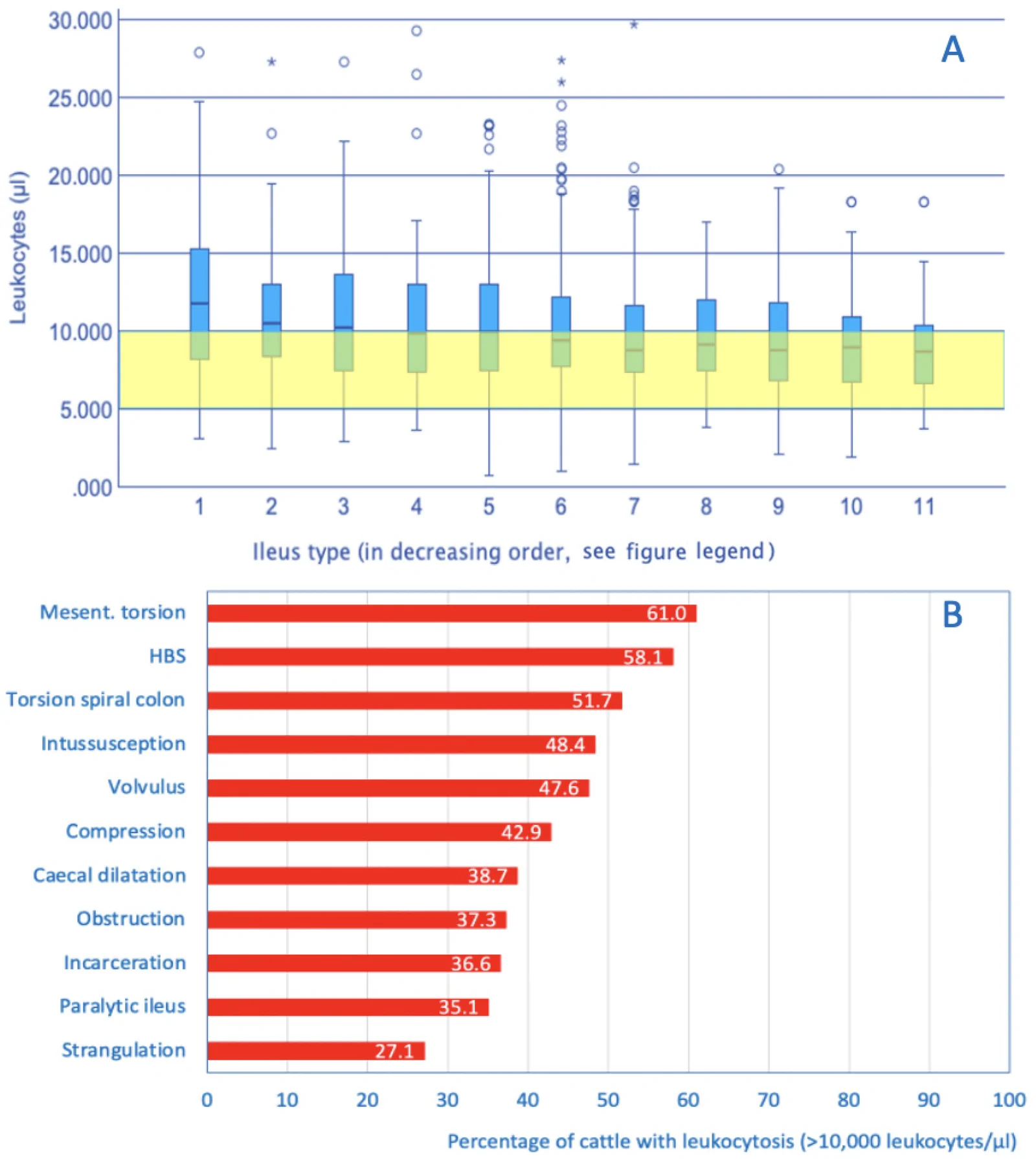
(**A**) Total white blood cell count (in decreasing order) in cattle with different types of ileus. 1 mesenteric torsion, 2 HBS, 3 torsion of the spiral colon, 4 volvulus, 5 intussusception, 6 caecal dilatation, 7 obstruction, 8 compression, 9 incarceration, 10 paralytic ileus, and 11 strangulations. ° outlier; * extreme score. Three extreme scores (32,700; 34,600; 35,100/µL) are not included in the boxplot. The yellow area marks the reference interval (5000 to 10,000 leukocytes/µL) for the total white blood cell count. (**B**) Prevalence of leukocytosis (defined as a total white blood cell count > 10,000/µL) in 1132 cattle with various types of ileus.

The median total leukocyte count was 9500/µL blood and ranged from 11,800/µL (mesenteric torsion, #1 in Figure 3A) to 8700/µL (strangulation, #11). It exceeded the reference interval of 5000 to 10,000 leukocytes/µL in cows with ileus types #1 to 3 (mesenteric torsion, HBS, and torsion of the spiral colon) and was within the reference interval in the remaining types of ileus. The total leukocyte count was significantly higher in cattle with mesenteric torsion (#1) than in cattle with obstruction (#7, *p* <0.05), incarceration (#9, *p* < 0.05), paralytic ileus (#10, *p* < 0.01), and strangulation (#11, *p* < 0.05) (for further details, see Table S4.1).

The prevalence of leukocytosis (defined as a total white blood cell count > 10,000/µL) ranged from 61.0% in cattle with mesenteric torsion to 27.1% in cattle with strangulation (Figure 3B). The prevalence was significantly higher in cattle with mesenteric torsion and HBS than in cattle with strangulation (*p* < 0.05; for further details, see Table S4.2).

### 4.4. Total Protein Concentration and Hyperproteinaemia

**Figure 4:**
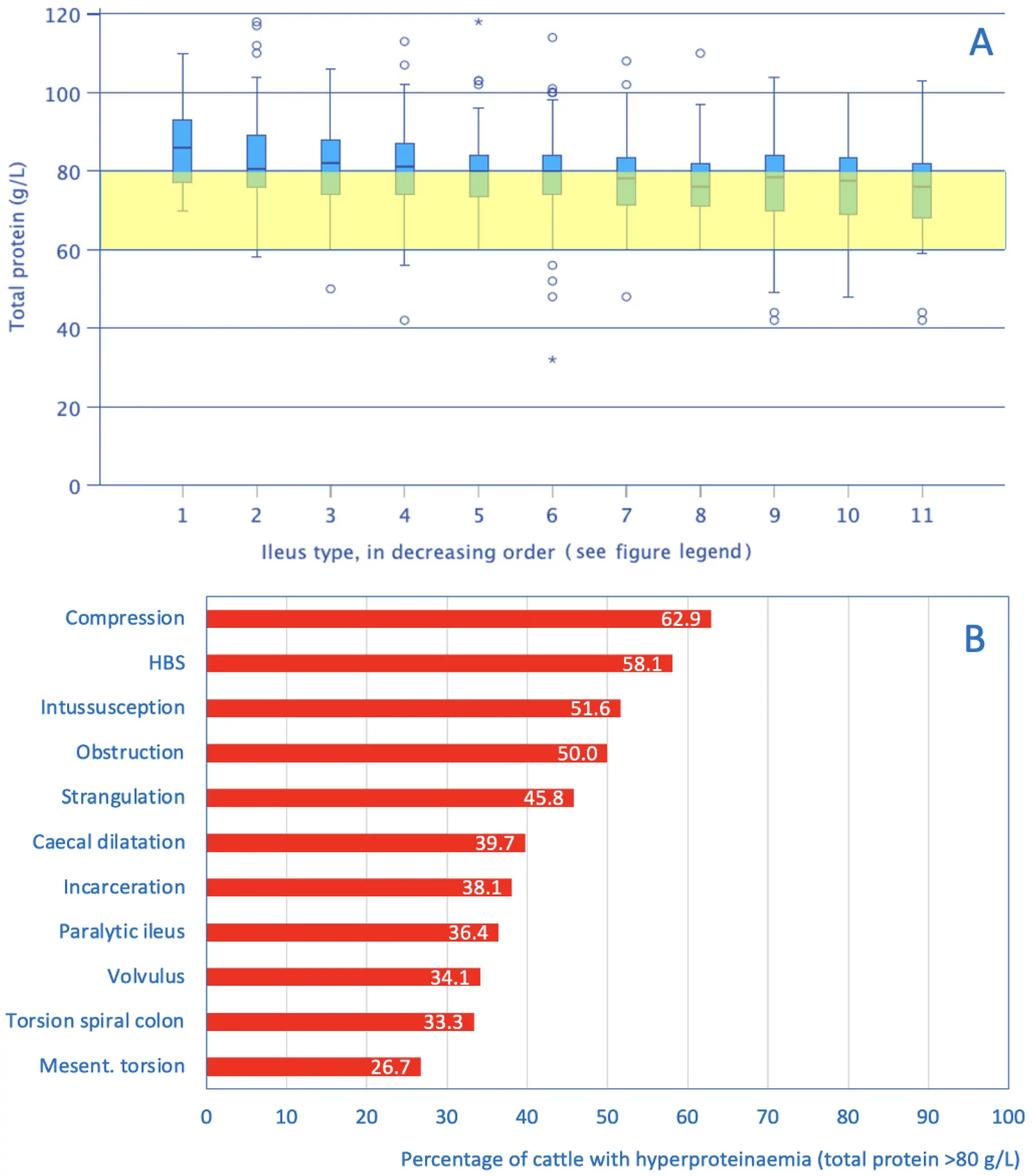
(**A**) Total protein concentration (in decreasing order) in cattle with various types of ileus. 1 compression, 2 obstruction, 3 HBS, 4 intussusception, 5 strangulation, 6 caecal dilatation, 7 paralytic ileus, 8 torsion of the spiral colon, 9 incarceration, 10 volvulus, and 11 mesenteric torsion. ° outlier; * extreme score. The yellow area marks the reference interval (60 to 80 g/L) for total protein concentration. (**B**) Hyperproteinaemia (total protein > 80 g/L) in 1135 cattle with various types of ileus.

The median total protein concentration was 80 g/L and ranged from 86.0 (compression, #1 in Figure 4A) to 76 g/L (mesenteric torsion, #11). The total protein concentration exceeded the reference interval of 60 to 80 g/L in cows with compression (#1), obstruction (#2), HBS (#3), and intussusception (#4) and was within the range in all remaining cows. Cows with compression (#1) had significantly higher total protein concentrations than cows with caecal dilatation (#6, *p* < 0.01), paralytic ileus (#7, *p* < 0.05), torsion of the spiral colon (#8, *p* < 0.01), incarceration (#9, *p* < 0.01), volvulus (#10, *p* < 0.01), and mesenteric torsion (#11, *p* < 0.01) (for further details and other significant differences, see Table S5.1).

The prevalence of hyperproteinaemia ranged from 62.9% (compression) to 26.7% (mesenteric torsion) (Figure 4B). Cattle with compression and HBS had hyperprotein-aemia significantly more often than cattle with mesenteric torsion (*p* < 0.05; for further details, see Table S5.2).

### 4.5. Urea Concentration and Azotaemia

**Figure 5:**
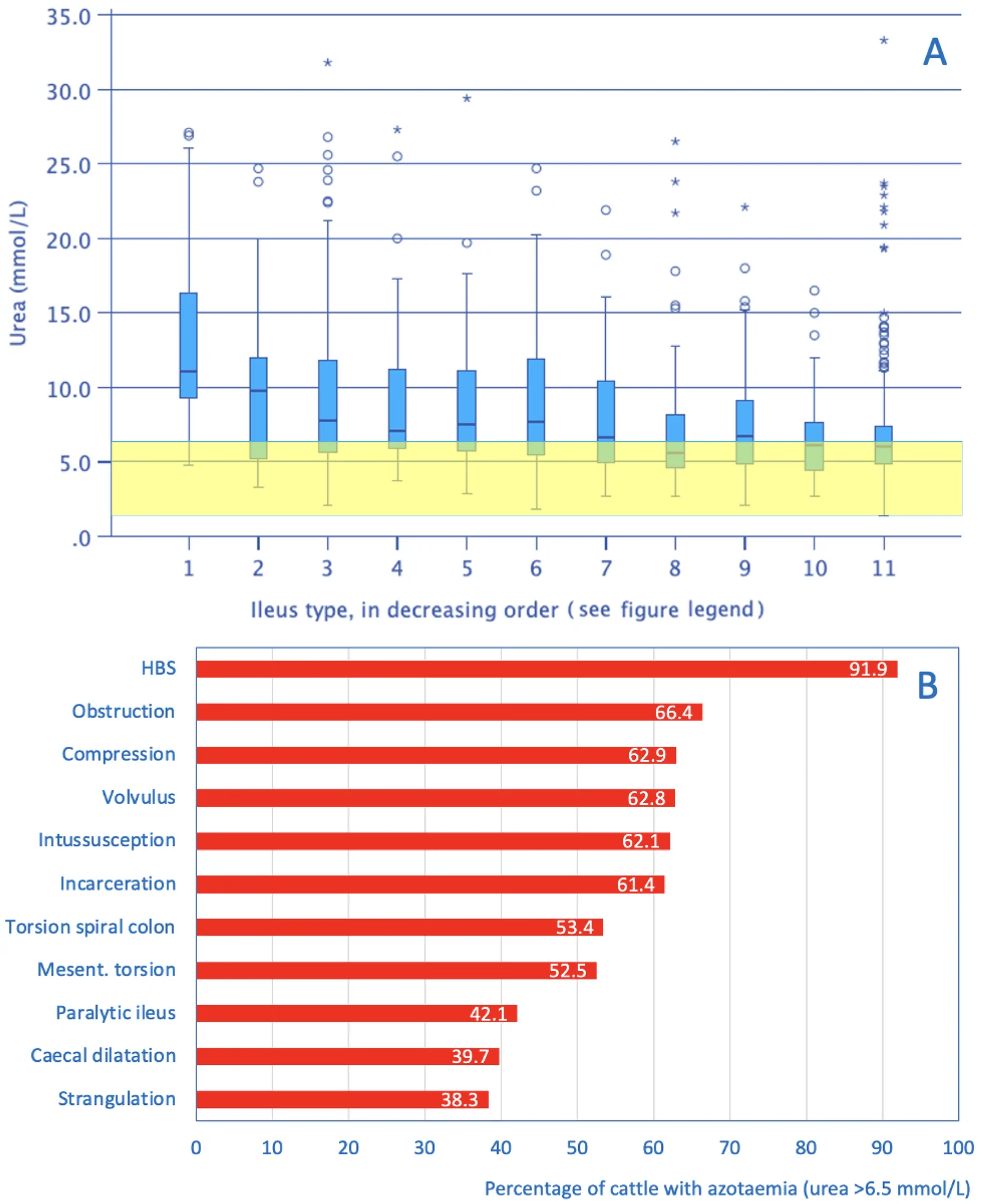
(**A**) Serum urea concentration (in decreasing order) in cattle with various types of ileus. 1 HBS, 2 compression, 3 intussusception, 4 volvulus, 5 incarceration, 6 obstruction, 7 mesenteric torsion, 8 strangulation, 9 torsion of the spiral colon, 10 paralytic ileus, and 11 caecal dilatation. Three extreme scores (35.8, 42.9, and 45.2 mmol/L) are not included in the boxplot. ° outlier; * extreme score. The yellow area indicates the reference interval (1.8 to 6.5 mmol/L) for urea concentration. (**B**) Azotaemia (serum urea > 6.5 mmol/L) in 1137 cattle with various types of ileus.

The median urea concentration was 6.7 mmol/L and ranged from 11.1 (HBS, #1) to 6.0 mmol/L (caecal dilatation, #11) (Figure 5A). It was in the reference interval (1.8 to 6.5 mmol/L) in cows with strangulation (#8), paralytic ileus (#10), and caecal dilatation (#11). It was increased in all the other cows. Except for cows with compression (#2), cows with HBS (#1) had significantly higher urea concentrations than cows with other types of ileus (*p* < 0.01; for further details and other significant differences, see Table S6.1).

The prevalence of azotaemia (defined as serum urea concentration > 6.5 mmol/L) ranged from 91.9% in cattle with HBS to 38.3% in cattle with strangulation (Figure 5B). Azotaemia was significantly more common in cattle with HBS than in cattle with other types of ileus (*p* < 0.05; for further details and other significant differences, see Table S6.2).

### 4.6. Calcium Concentration and Hypocalcaemia

**Figure 6:**
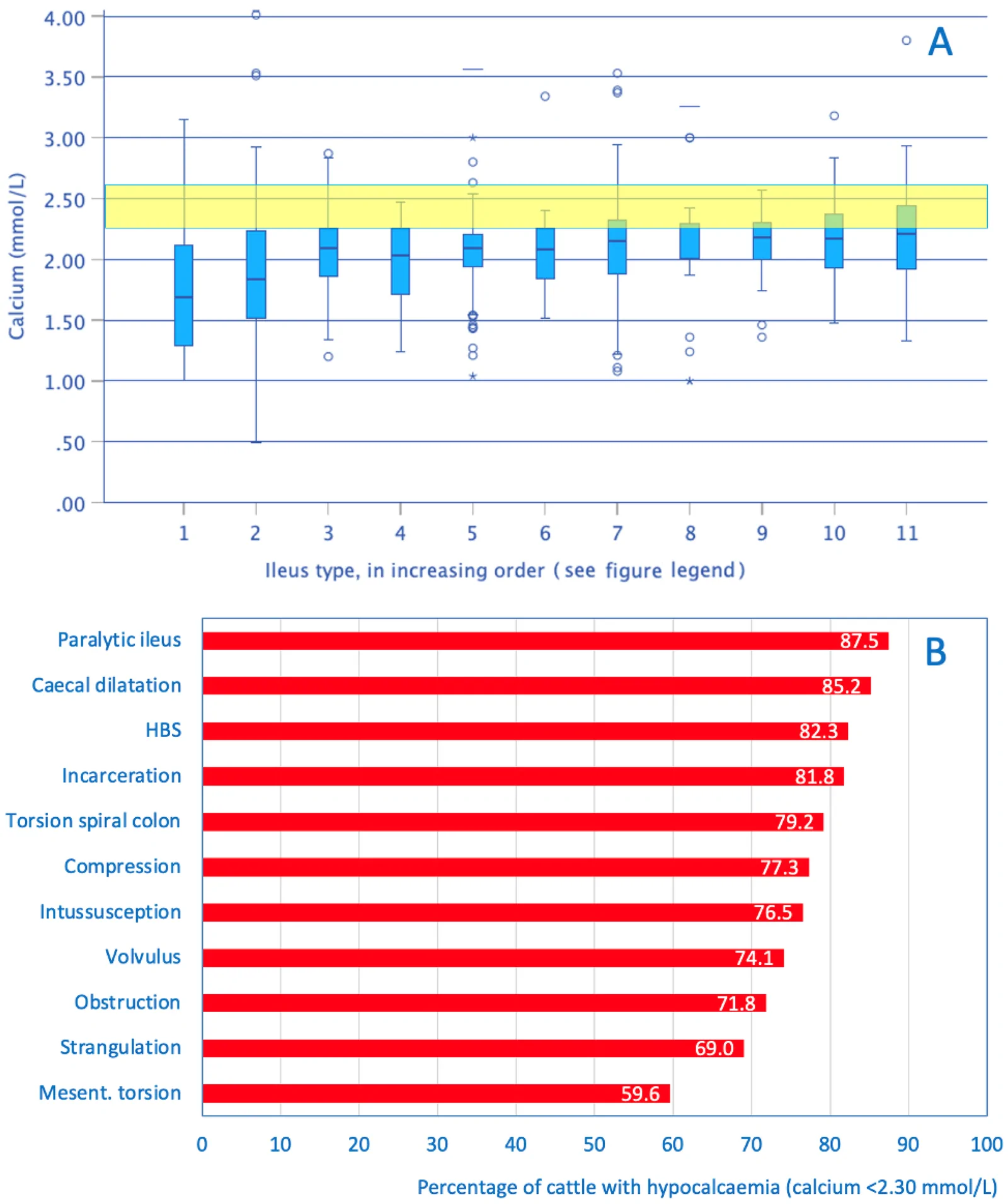
(**A**) Serum calcium concentration (in increasing order) in cattle with various types of ileus. 1 paralytic ileus, 2 HBS, 3 incarceration, 4 volvulus, 5 caecal dilatation, 6 torsion of the spiral colon, 7 obstruction, 8 intussusception, 9 compression, 10 strangulation, and 11 mesenteric torsion. Two outliers (4.01 and 4.07 mmol/L) and two extreme scores (4.34 and 5.75 mmol/L) are not included in the boxplot. ° outlier; * extreme score. The yellow area marks the reference interval (2.30 to 2.60 mmol/L) for blood serum calcium concentration. (**B**) Hypocalcaemia (defined as serum calcium concentration < 2.30 mmol/L) in 756 cattle with various types of ileus.

The median calcium concentration was 2.08 mmol/L and ranged from 1.69 (paralytic ileus, #1) to 2.21 mmol/L (mesenteric torsion, #11) (Figure 6A). The median concentration for all types of ileus was below the reference interval of 2.30 to 2.60 mmol/L. Cows with incarceration (#3, *p* < 0.05), caecal dilatation (#5, *p* < 0.01), obstruction (#7, *p* < 0.01), intussusception (#8, *p* < 0.01), compression (#9, *p* < 0.05), strangulation (#10, *p* < 0.01), and mesenteric torsion (#11, *p* < 0.01) had significantly higher median calcium concentrations than cows with paralytic ileus (#1) (for further details and other significant differences, see Table S7.1).

The prevalence of hypocalcaemia ranged from 87.5% (paralytic ileus) to 59.6% (mesenteric torsion) (Figure 6B). The proportion of hypocalcaemia did not differ among cattle with various types of ileus (for further details, see Table S7.2).

### 4.7. Magnesium Concentration and Hypermagnesaemia

**Figure 7:**
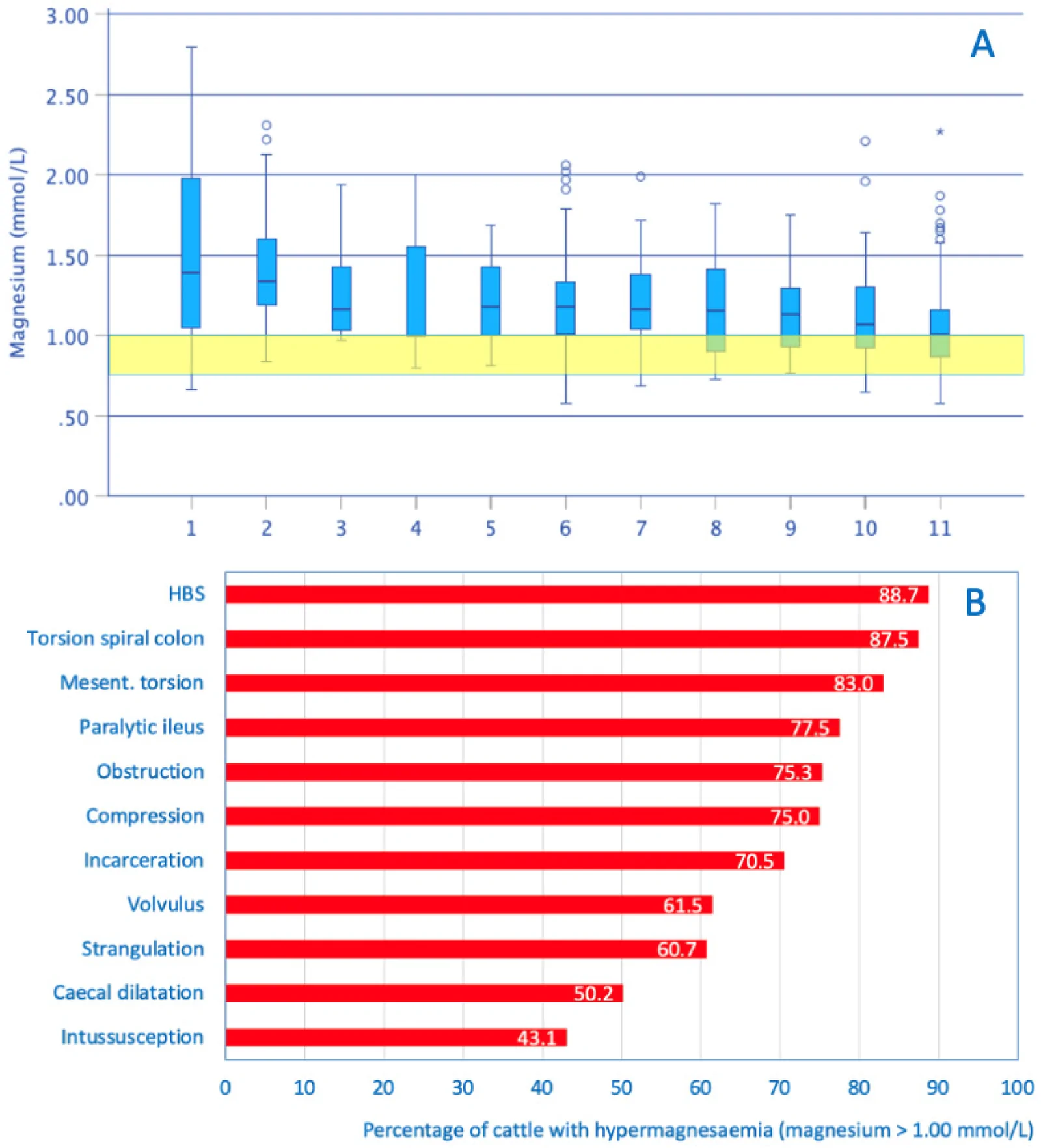
(**A**) Serum magnesium concentration (in decreasing order) in cattle with various types of ileus. 1 mesenteric torsion, 2 HBS, 3 torsion of the spiral colon, 4 intussusception, 5 compression, 6 obstruction, 7 paralytic ileus, 8 volvulus, 9 incarceration, 10 strangulation, and 11 caecal dilatation. One extreme score (3.98 mmol/L) is not included in the boxplot. ° outlier; * extreme score. The yellow area marks the reference interval (0.80 to 1.00 mmol/L) for serum magnesium concentration. (**B**) Hypermagnesaemia (defined as serum magnesium concentration > 1.00 mmol/L) in 750 cattle with various types of ileus.

The median magnesium concentration was 1.10 mmol/L and ranged from 1.39 (mesenteric torsion, #1) to 1.00 mmol/L (intussusception, #4) (Figure 7A). Except for cows with intussusception (#4; 1.00 mmol/L), it exceeded the reference interval (0.80 to 1.00 mmol/L). Cows with mesenteric torsion (#1) had significantly higher median magnesium concentrations than cows with intussusception (#4, *p* < 0.01), compression (#5, *p* < 0.05), obstruction (#6, *p* < 0.01), paralytic ileus (#7, *p* < 0.01), volvulus (#8, *p* < 0.01), incarceration (#9, *p* < 0.01), strangulation (#10, *p* < 0.01), and caecal dilatation (#11, *p* < 0.01). Cattle with HBS (#2) had significantly higher concentrations than cattle with obstruction (#6, *p* < 0.01), paralytic ileus (#7, *p* < 0.05), incarceration (#9, *p* < 0.01), strangulation (#10, *p* < 0.01), and caecal dilatation (#11, *p* < 0.01) (for further details, Table S8.1).

The prevalence of hypermagnesaemia (defined as serum magnesium concentration > 1.00 mmol/L) varied from 88.7% in cattle with HBS to 43.1% in cattle with intussusception (Figure 7B). Hypermagnesaemia occurred significantly more often in cattle with HBS, torsion of the spiral colon, mesenteric torsion, and obstruction than in cattle with caecal dilatation or intussusception (*p* < 0.05; for further details, see Table S8.2).

### 4.8. Inorganic Phosphate and Hypophosphataemia

**Figure 8:**
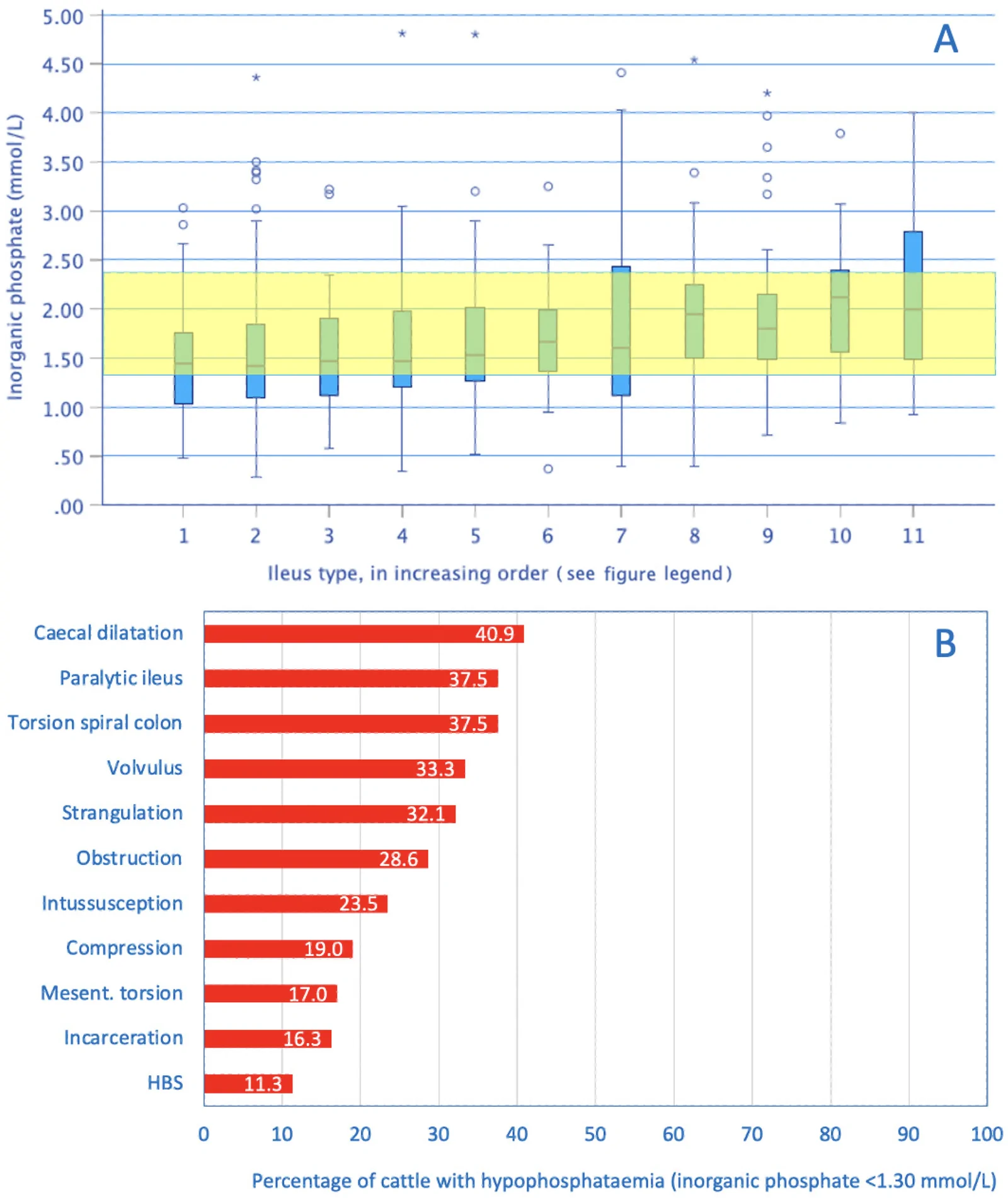
(**A**) Inorganic phosphate concentration (in increasing order) in cattle with various types of ileus. 1 paralytic ileus, 2 caecal dilatation, 3 torsion of the spiral colon, 4 strangulation, 5 obstruction, 6 compression, 7 volvulus, 8 incarceration, 9 mesenteric torsion, 10 HBS, and 11 intussusception. One extreme score (5.4 mmol/L) is not included in the boxplot. ° outlier; * extreme score. The yellow area marks the reference interval (1.30 to 2.40 mmol/L) for serum inorganic phosphate concentration. (**B**) Hypophosphataemia (defined as inorganic phosphate concentration < 1.30 mmol/L) in 750 cattle with various types of ileus.

The median inorganic phosphate concentration was 1.58 mmol//L and ranged from 1.44 (paralytic ileus, #1) to 2.00 mmol/L (intussusception, #11) (Figure 8A). It was within the reference interval (1.30 to 2.40 mmol/L) for all ileus types and was significantly lower (*p* < 0.01) in cows with paralytic ileus (#1) than in cows with HBS (#10) and intussusception (#11). Cattle with caecal dilatation (#2) had significantly lower inorganic phosphate concentrations (*p* < 0.01) than those with incarceration (#8), mesenteric torsion (#9), HBS (#10), and intussusception (#11) (for further details and other significant differences, see Table S9.1).

The prevalence of hypophosphataemia ranged from 40.9% (caecal dilatation) to 11.3% (HBS) (Figure 8B). Hypophosphataemia was significantly more common in cattle with caecal dilatation than in cattle with HBS (*p* < 0.05; for further details, see Table S9.2).

### 4.9. Potassium Concentration and Hypokalaemia

**Figure 9:**
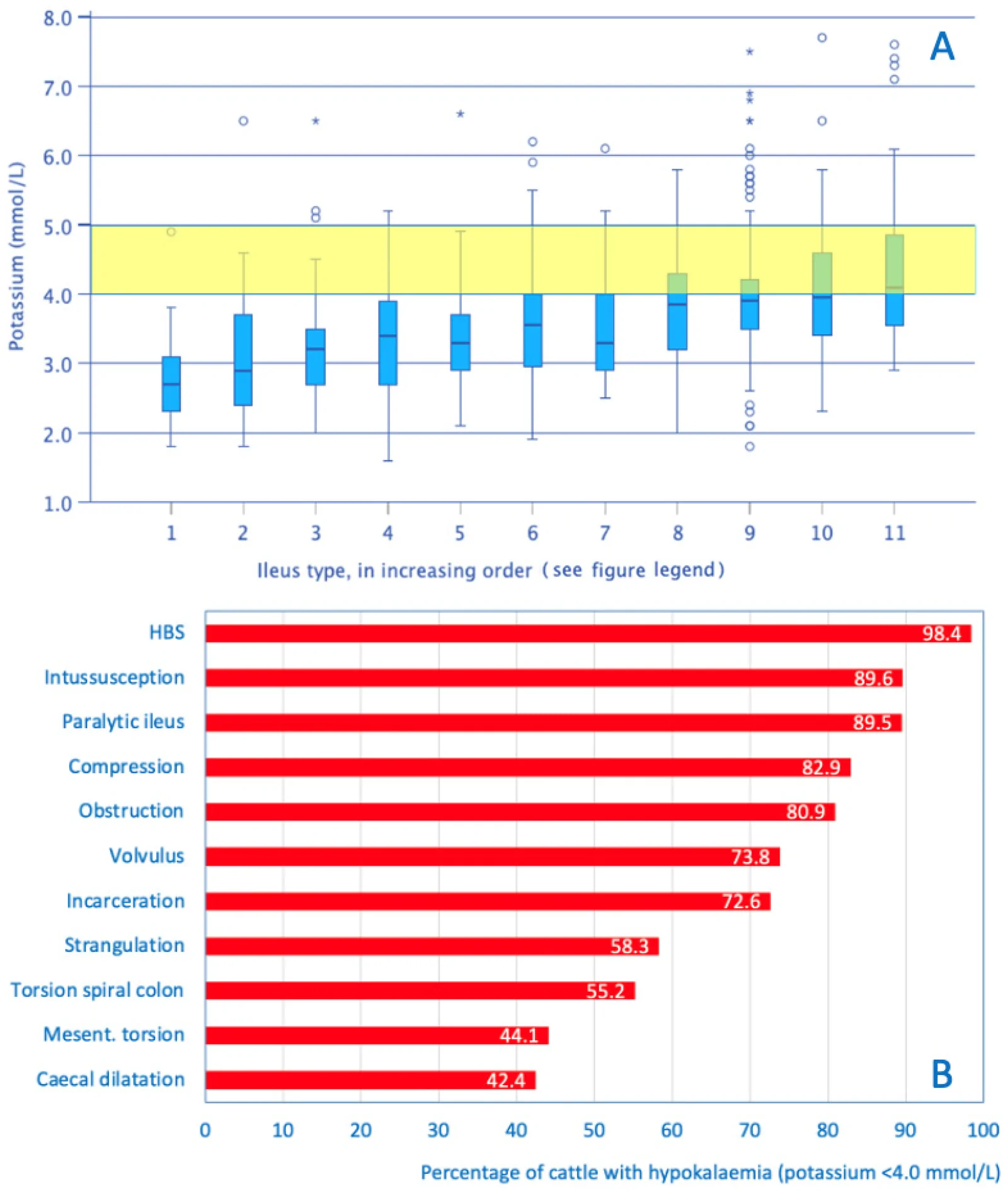
(**A**) Serum potassium concentration (in increasing order) in cattle with various types of ileus. 1 HBS, 2 compression, 3 intussusception, 4 obstruction, 5 paralytic ileus, 6 incarceration, 7 volvulus, 8 strangulation, 9 caecal dilatation, 10 torsion of the spiral colon, and 11 mesenteric torsion. ° outlier; * extreme score. The yellow area marks the reference interval (4.0 to 5.0 mmol/L) for serum potassium concentration. (**B**) Hypokalaemia (defined as serum potassium concentration < 4.00 mmol/L) in 1135 cattle with various types of ileus.

The median potassium concentration was 3.60 mmol/L and ranged from 2.70 (HBS, #1) to 4.10 mmol/L (mesenteric torsion, #11) (Figure 9A). Except for cows with mesenteric torsion (#11), the concentration was below the reference interval (4.00 to 5.00 mmol/L). Cows with HBS (#1) had significantly lower potassium concentrations (*p* < 0.01) than cows with all other ileus types, except for cows with compression (#2) (for further details and other significant differences, see Table S10.1).

The prevalence of hypokalaemia ranged from 98.4% in cattle with HBS to 42.4% in cattle with caecal dilatation (Figure 9B). Cattle with HBS had hypokalaemia significantly more often than cattle with volvulus, incarceration, strangulation, torsion of the spiral colon, mesenteric torsion, or caecal dilatation (*p* < 0.05; for further details and other significant differences, see Table S10.2). Hypokalaemia occurred significantly more often in cattle with intussusception than in cattle with strangulation, torsion of the spiral colon, mesenteric torsion, or caecal dilatation.

### 4.10. Chloride Concentration and Hypochloraemia

**Figure 10:**
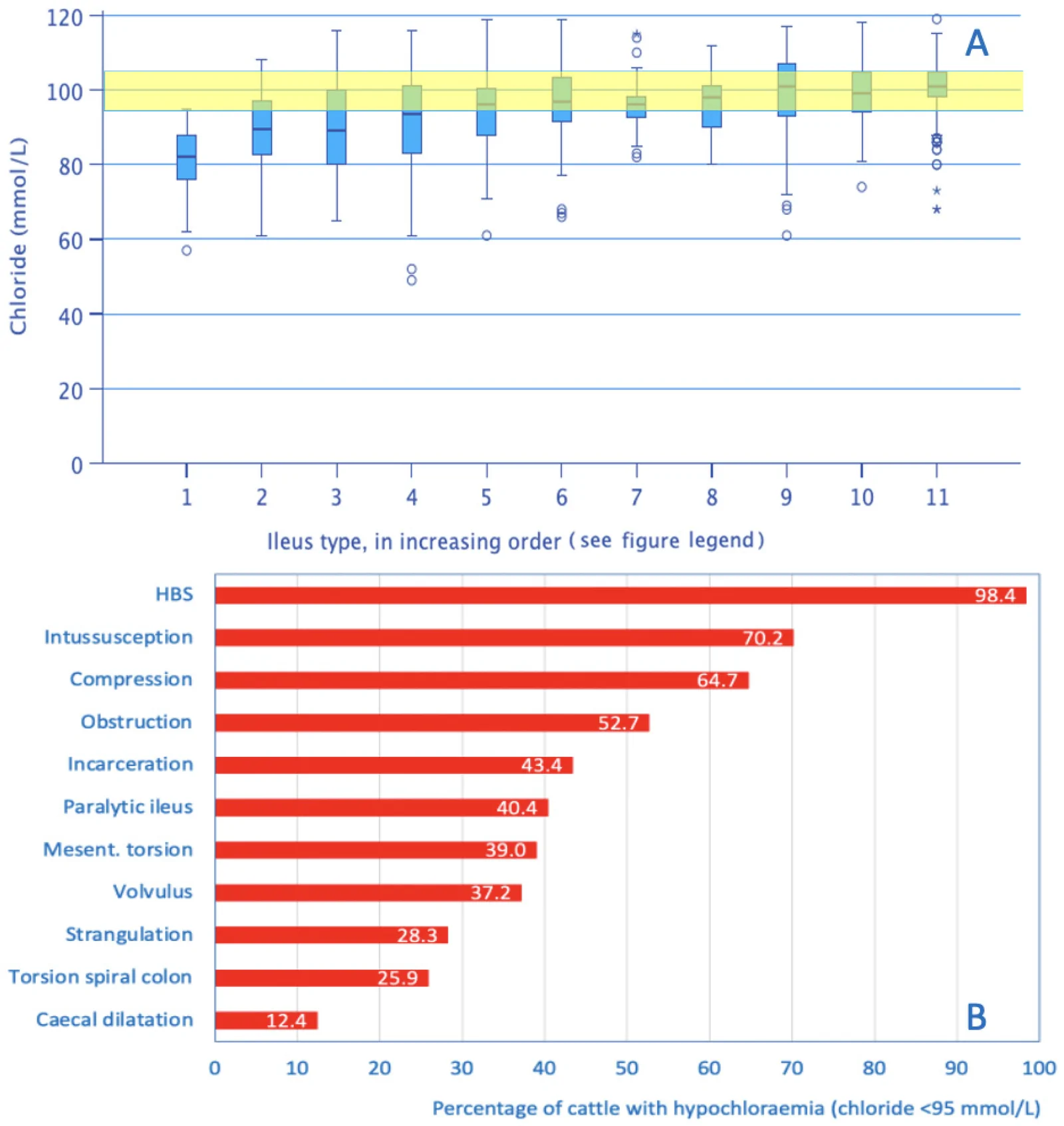
(**A**) Serum chloride concentration (in increasing order) in cattle with various types of ileus. 1 HBS, 2 intussusception, 3 compression, 4 obstruction, 5 incarceration, 6 volvulus, 7 mesenteric torsion, 8 paralytic ileus, 9 strangulation, 10 torsion of the spiral colon, and 11 caecal dilatation. ° outlier; * extreme score. The yellow area marks the reference interval (95 to 105 mmol/L) for serum chloride concentration. (**B**) Hypochloraemia (defined as serum chloride concentration < 95 mmol/L) in 1135 cattle with various types of ileus.

The median chloride concentration was 98 mmol//L and ranged from 82 (HBS, #1) to 101 mmol/L (caecal dilatation, #11) (Figure 10A). It was below the reference interval of 95 to 105 mmol/L in cattle with HBS (#1), intussusception (#2), compression (#3), and obstruction (#14) and within the reference interval in cattle with the remaining ileus types. The chloride concentration was significantly lower in cows with HBS (#1) compared with all other ileus types (for further details and other significant differences, see Table S11.1).

The prevalence of hypochloraemia ranged from 98.4% in cattle with HBS to 12.4% in cattle with caecal dilatation (Figure 10B). Cattle with HBS had hypochloraemia significantly more often (*p* < 0.01) than cattle with all other types of ileus. In cattle with intussusception, hypochloraemia occurred significantly more often than in cattle with incarceration, paralytic ileus, mesenteric torsion, volvulus, strangulation, torsion of the spiral colon, and caecal dilatation (*p* < 0.01; for further details and other significant differences, see Table S11.2).

### 4.11. Blood pH Abnormalities

**Figure 11:**
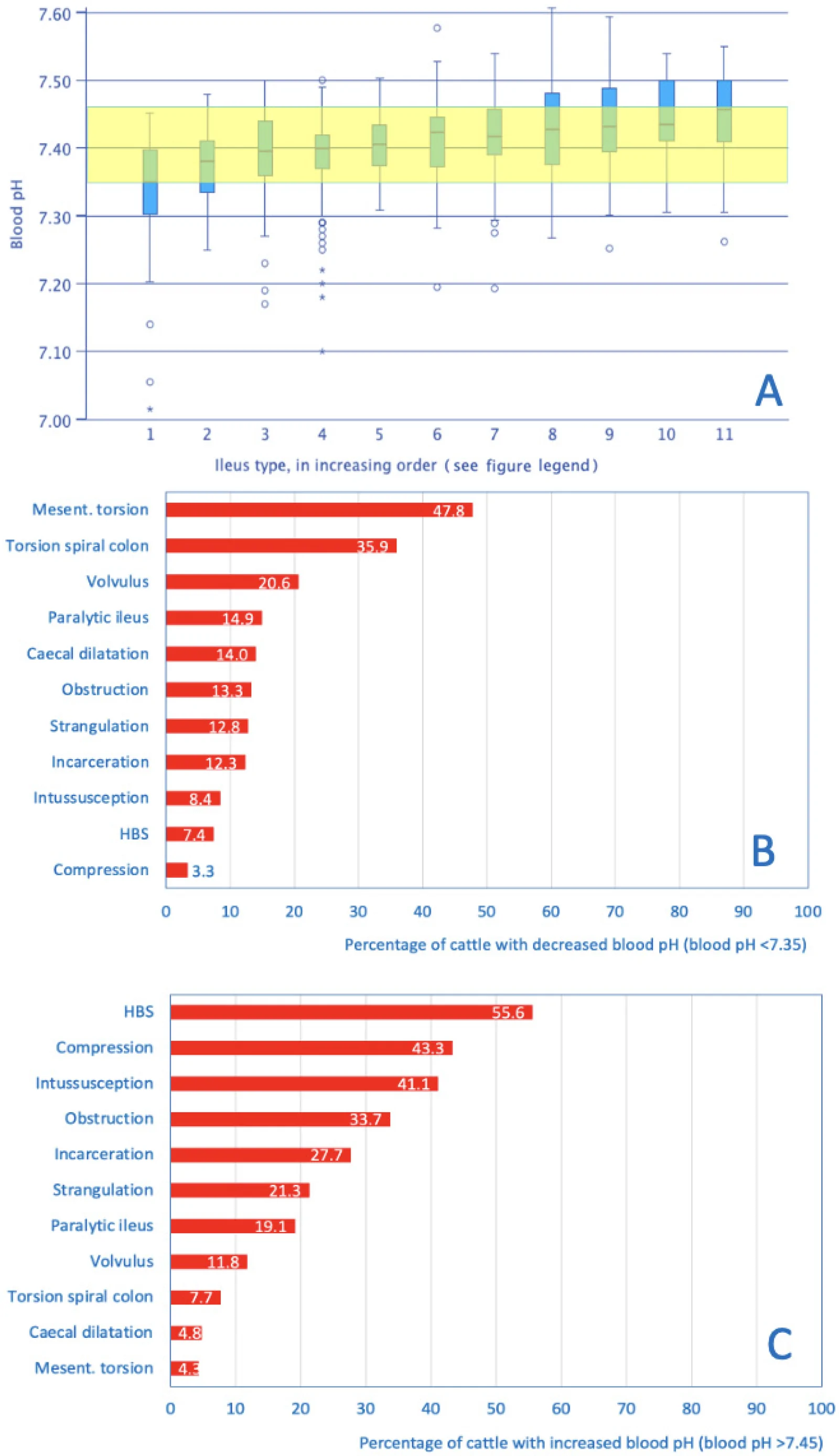
(**A**) Blood pH (in increasing order) in cattle with various types of ileus. 1 mesenteric torsion, 2 torsion of the spiral colon, 3 volvulus, 4 caecal dilatation, 5 paralytic ileus, 6 strangulation, 7 incarceration, 8 obstruction, 9 intussusception, 10 compression, and 11 HBS. Two outliers (7.61 and 7.63) are not included in the boxplot. ° outlier; * extreme score. The yellow area marks the reference interval (7.35 to 7.45) for blood pH. (**B**) Decreased blood pH (defined as blood pH < 7.35 mmol/L) in 988 cattle with various types of ileus. (**C**) Increased blood pH (defined as blood pH > 7.45 mmol/L) in 988 cattle with various types of ileus.

The median blood pH was 7.40 and ranged from 7.35 (mesenteric torsion, #1) to 7.46 (HBS, #11) (Figure 11A). Cattle with mesenteric torsion (#1) had a significantly lower blood pH than all other cattle (*p* < 0.01), except for cattle with torsion of the spiral colon (#2) (for further details and other significant differences, see Table S12.1).

The prevalence of **decreased blood pH**, defined as blood pH < 7.35, ranged from 47.8% in cattle with mesenteric torsion to 3.3% in cattle with compression (Figure 11B). A blood pH < 7.35 was significantly more common (*p* < 0.05) in cattle with mesenteric torsion and torsion of the spiral colon than in cattle with caecal dilatation, intussusception, or HBS (for further details and other significant differences, see Table S12.2).

The prevalence of **increased blood pH**, defined as a blood pH > 7.45, ranged from 55.6% (HBS) to 4.3% (mesenteric torsion) (Figure 11C). The blood pH was increased significantly more often (*p* < 0.05) in cattle with HBS, compression, and intussusception than in cattle with torsion of the spiral colon, caecal dilatation, or mesenteric torsion (for further details and other significant differences, see Table S12.3).

### 4.12. Base Excess

**Figure 12:**
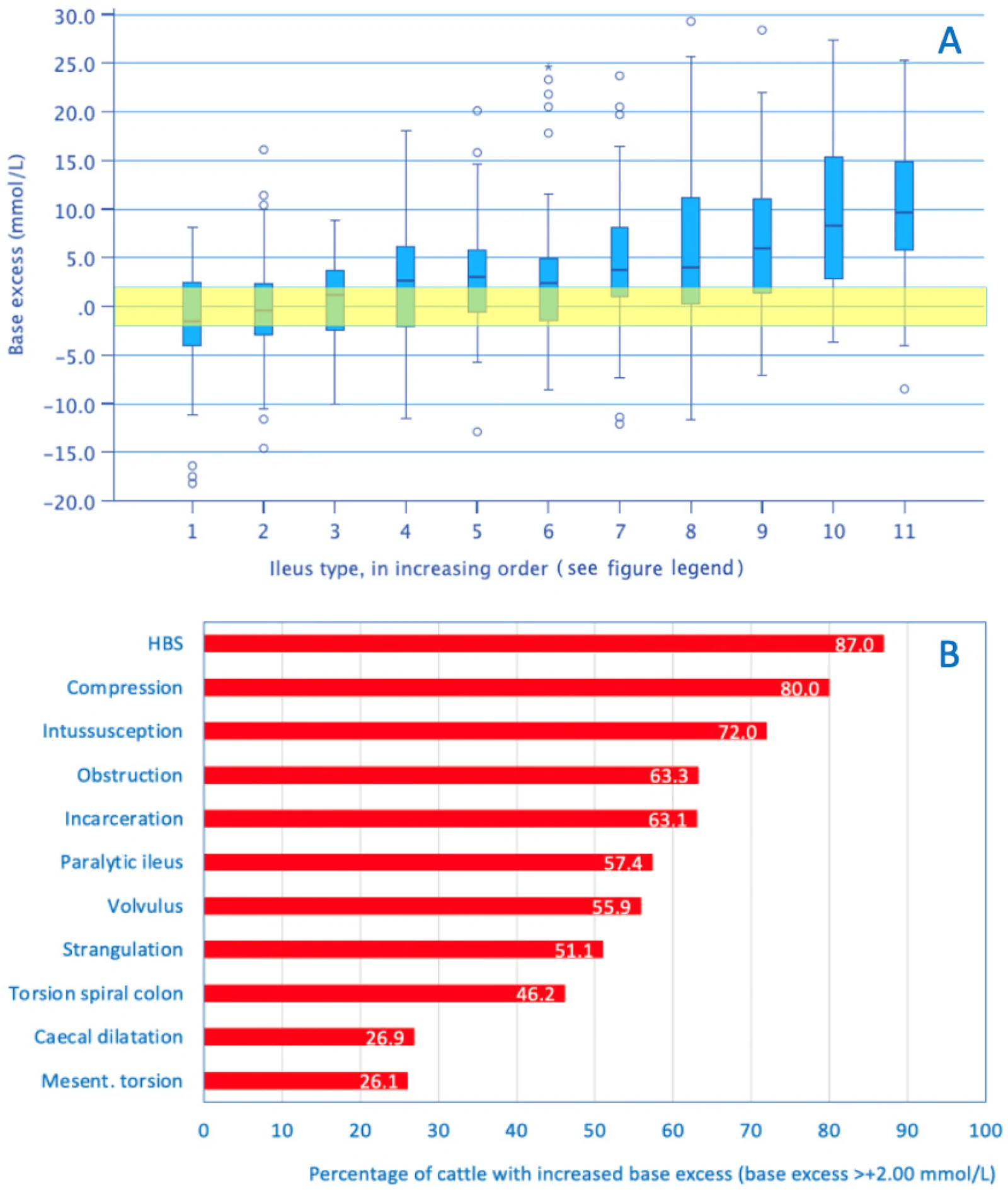
(**A**) Base excess (in increasing order) in cattle with various types of ileus. 1 mesenteric torsion, 2 caecal dilatation, 3 torsion of the spiral colon, 4 volvulus, 5 paralytic ileus, 6 strangulation, 7 incarceration, 8 obstruction, 9 intussusception, 10 compression, and 11 HBS. Three outliers (31.2, 33.6, and 35.7 mmol/L) and one extreme score (36.5 mmol/L) are not included in the boxplot. ° outlier; * extreme score. The yellow area marks the reference interval (−2 to +2 mmol/L) for base excess. (**B**) Increased base excess (defined as base excess > +2.00 mmol/L) in 987 cattle with various types of ileus.

The median base excess was 2.81 mmol/L and ranged from −1.50 (mesenteric torsion, #1) to +9.60 mmol/L (HBS, #11) (Figure 12A). It was in the reference interval (−2 to +2 mmol/L) in cattle with mesenteric torsion (#1), caecal dilatation (#2), and torsion of the spiral colon (#3) and exceeded the reference range in all other ileus types. Cattle with mesenteric torsion (#1) had a significantly lower base excess (*p* < 0.01) than cattle with paralytic ileus (#5), strangulation (#6), incarceration (#7), obstruction (#8), intussusception (#9), compression (#10), and HBS (#11) (for further details and other significant differences, see Table S13.1).

The prevalence of increased base excess varied from 87.0% in cattle with HBS to 26.1% in cattle with mesenteric torsion (Figure 12B). An increase in base excess occurred significantly more often in cattle with HBS than in cattle with paralytic ileus, strangulation, torsion of the spiral colon, caecal dilatation, and mesenteric torsion (*p* < 0.05; for further details and other significant differences, see Table S13.2).

### 4.13. Rumen Chloride Concentration

**Figure 13:**
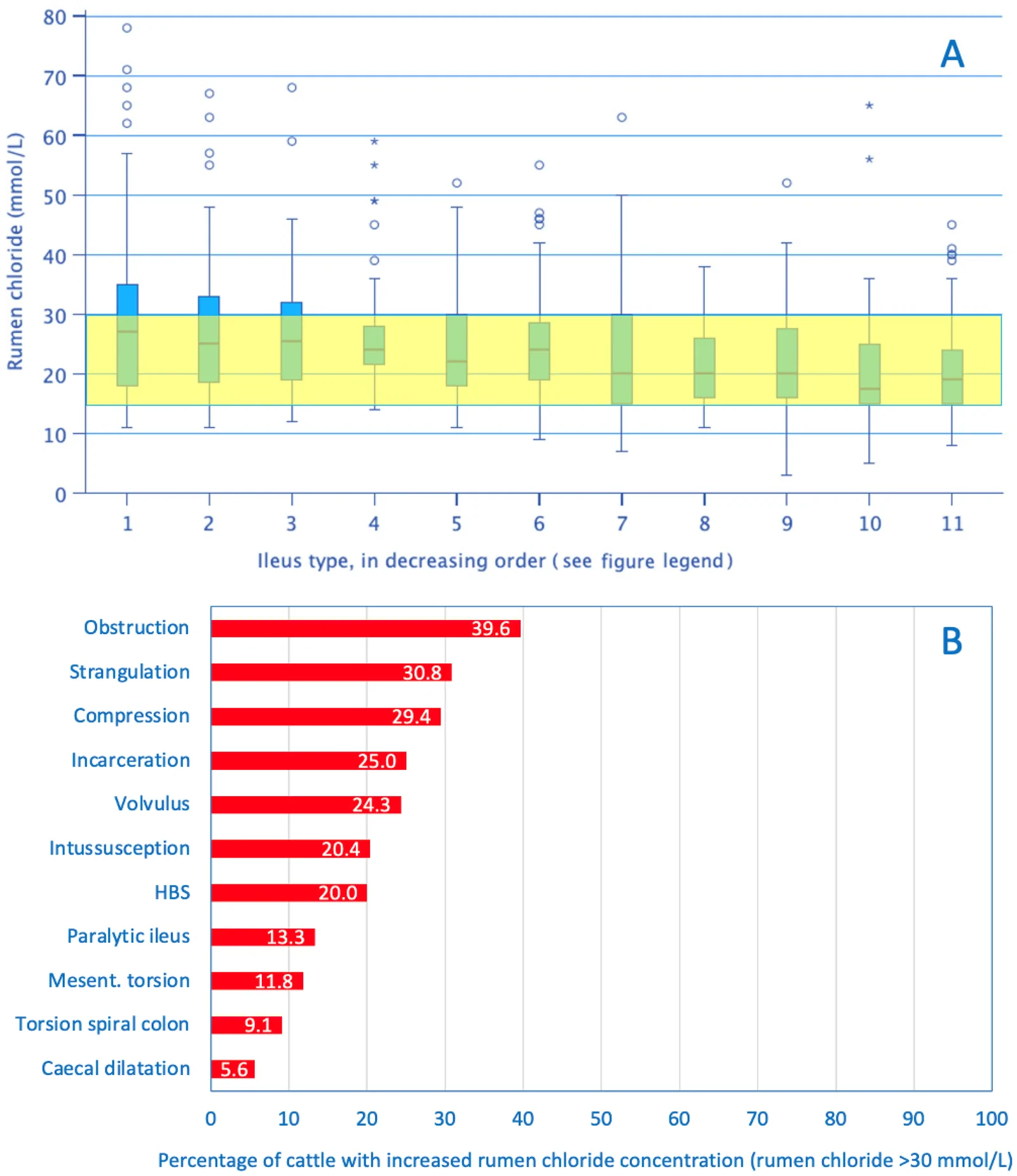
(**A**) Rumen chloride concentration (in decreasing order) in cattle with various types of ileus. 1 obstruction, 2 strangulation, 3 compression, 4 HBS, 5 incarceration, 6 intussusception, 7 volvulus, 8 paralytic ileus, 9 torsion of the spiral colon, 10 mesenteric torsion, and 11 caecal dilatation. Four extreme scores (86, 90, 101, and 185 mmol/L) are not included in the boxplot. ° outlier; * extreme score. The yellow area marks the reference interval (15 to 30 mmol/L) for rumen chloride concentration. (**B**) Increased rumen chloride concentration (defined as rumen chloride > 30 mmol/L) in 972 cattle with various types of ileus.

The median rumen chloride concentration was 21.0 mmol/L and ranged from 27 (obstruction, #1) to 17.5 mmol/L (mesenteric torsion, #11) (Figure 13A). It was in the reference interval (15 to 30 mmol/L) for all ileus types. The rumen chloride concentration was significantly higher in cows with obstruction (#1) than in cows with intussusception (#6, *p* < 0.01), volvulus (#7, *p* < 0.05), paralytic ileus (#8, *p* < 0.01), torsion of the spiral colon (#9, *p* < 0.01), mesenteric torsion (#10, *p* < 0.01), and caecal dilatation (#11, *p* < 0.01; for further details and other significant differences, see Table S14.1).

The prevalence of increased rumen chloride concentration varied from 39.6% (obstruction) to 5.6% (caecal dilatation) (Figure 13B). Cattle with obstruction had increased rumen chloride concentrations significantly more often (*p* < 0.05) than cattle with torsion of the spiral colon and caecal dilatation (for further details and other significant differences, see Table S14.2).

### 4.14. Correlations Among the Laboratory Variables

**Figure 14:**
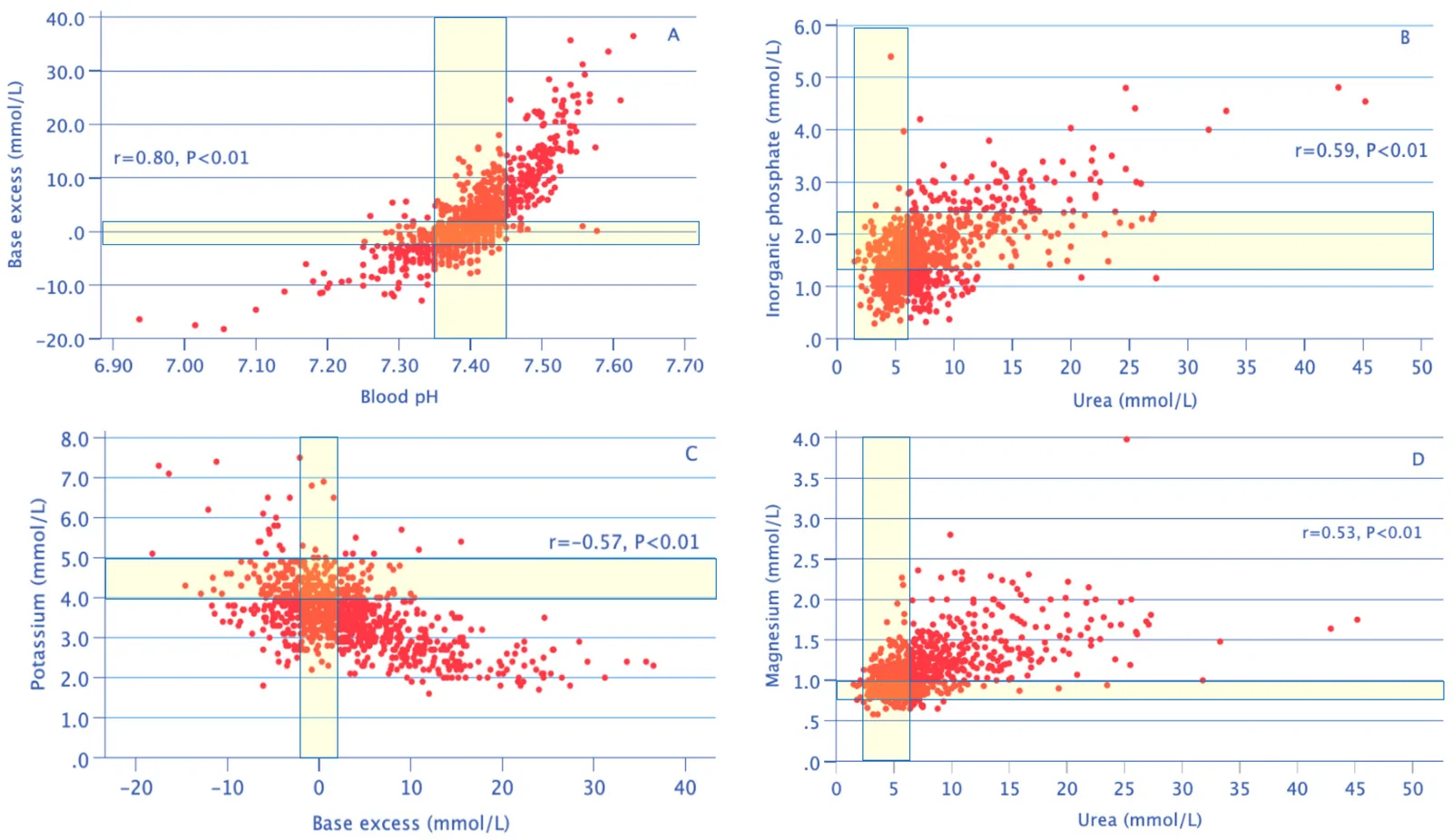
Relationships between blood pH and base excess (**A**), urea and inorganic phosphate (**B**), base excess and potassium (**C**), and urea and magnesium (**D**) in cattle with various types of ileus. The yellow areas mark the reference intervals for the variables on the x- and y-axes. Each red dot represents a cow with the ordinates and abscissas of the depicted variable.

A total of 66 correlations were calculated among the 12 variables; 52 were significant (*p* < 0.05, or *p* < 0.01) and 14 were non-significant (Table S15). Non-significant correlations were observed in nine relationships between the total white blood cell count and all the other variables except for total protein and serum chloride concentrations. The other five non-significant correlations were between calcium and urea, calcium and chloride, potassium and magnesium, potassium and inorganic phosphate, and haematocrit and calcium. However, only 7 of the 52 significant Pearson correlations had coefficients > 0.50, and the remaining 45 coefficients were <0.50. The largest coefficients occurred between blood pH and base excess (r = 0.80, *p* < 0.01, Figure 14A), chloride and base excess (r = −0.66, *p* < 0.01), urea and chloride (r = −0.63, *p* < 0.01), urea and inorganic phosphate (r = 0.59, *p* < 0.01, Figure 14B), base excess and potassium (r = −0.57, *p* < 0.01, Figure 14C), urea and magnesium (r = 0.53, *p* < 0.01, Figure 14D), and blood pH and potassium (r = −0.51, *p* < 0.01).

## 5. Ultrasonographic Findings in Cattle with Ileus

### 5.1. Overview

**Figure 15:**
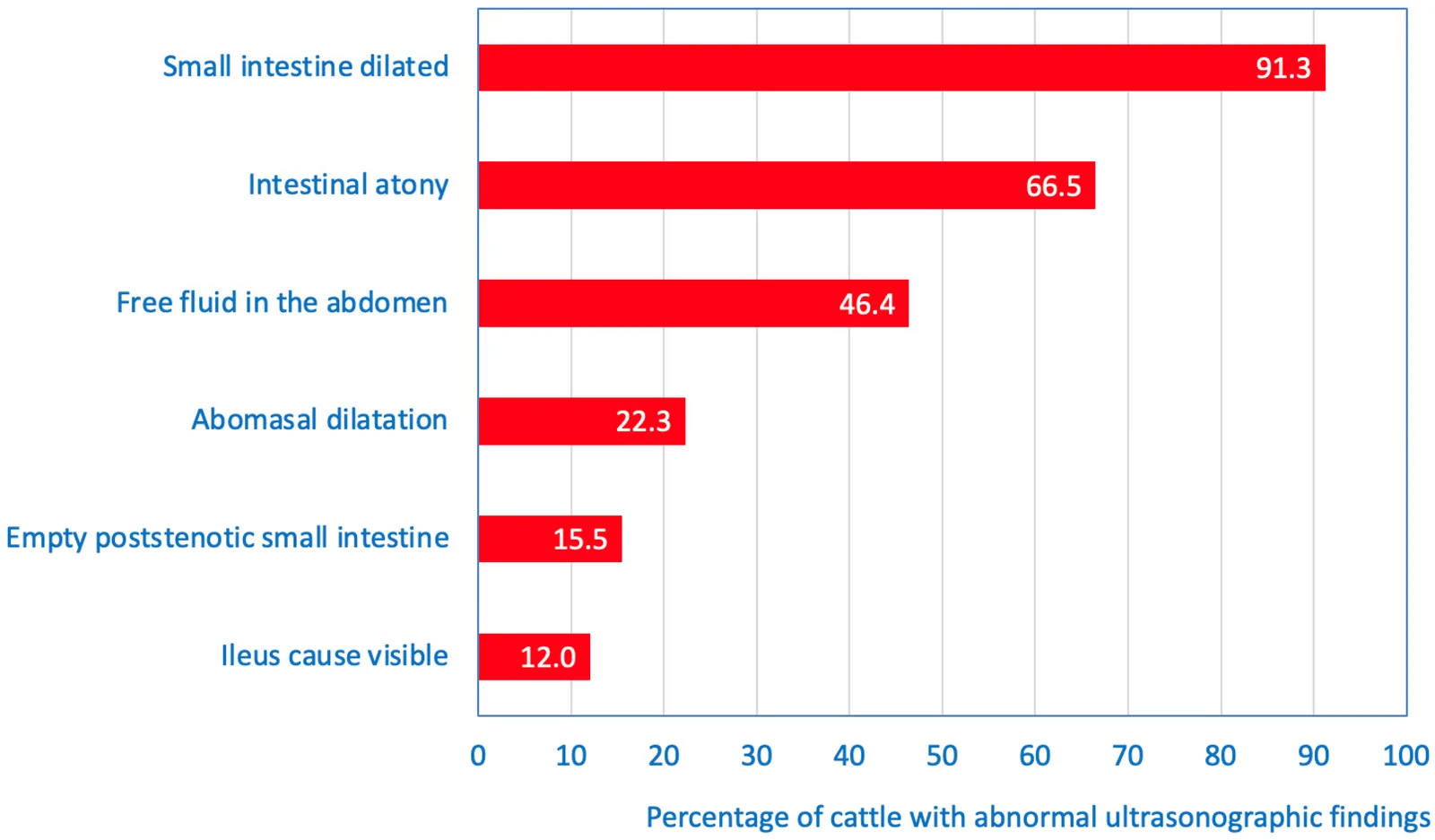
The most common abnormal ultrasonographic findings in 575 cattle with caecal dilatation (n = 30), compression (n = 23), HBS (n = 63), incarceration (n = 68), intussusception (n = 123), mesenteric torsion (n = 37), obstruction (n = 89), paralytic ileus (n = 50), strangulation (n = 43), torsion of the spiral colon (n = 25), and volvulus (n = 31).

The main abnormalities were dilated small intestine (91.3%, 524/574), intestinal atony (66.5%, 357/537), increased free fluid in the abdomen (46.4%, 275/593), abomasal dilatation (22.3%, 130/582), and empty poststenotic small intestine (15.5%, 90/582) (Figure 15, Table S16). The cause of the ileus was visible in 12.0% (70/582) of the examined cattle. In addition, the large intestine was dilated in 77 out of 169 examined cattle.

### 5.2. Dilated Intestine

**Figure 16:**
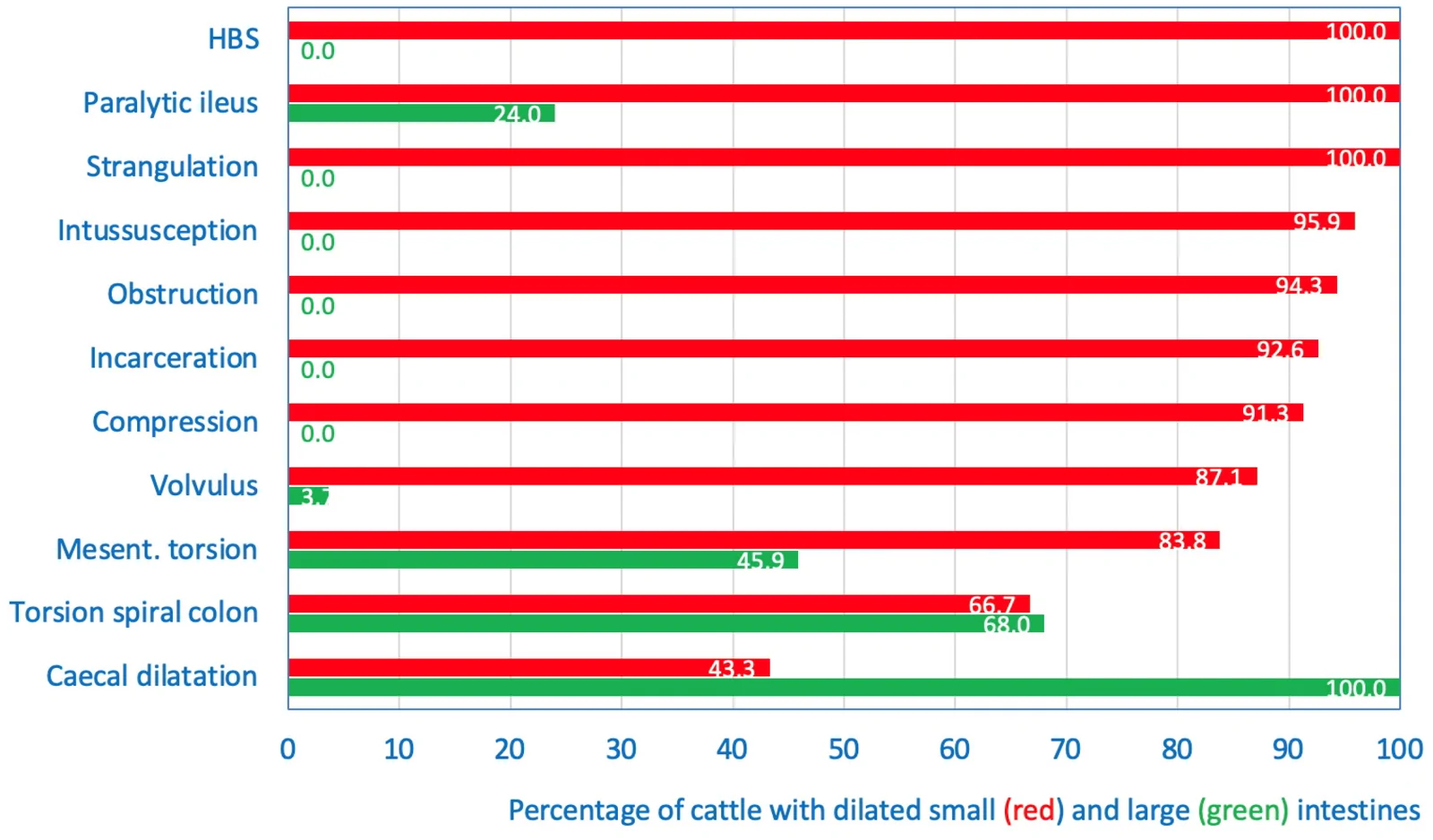
Dilated small and large intestines in 574 cattle with various types of ileus.

A dilated small intestine was recorded in cattle with all types of ileus. The prevalence of dilated small intestine varied from 100% (HBS) to 43.3% (caecal dilatation). By contrast, dilatation of the large intestine only occurred in cattle with caecal dilatation (100%), torsion of the spiral colon (68.0%), mesenteric torsion (45.9%), paralytic ileus (24.0%), and volvulus (3.7%) (Figure 16, Table S16). Only the dilated small intestine was seen in cattle with HBS, strangulation, intussusception, obstruction, incarceration, and compression. In contrast, small and large intestinal dilatation occurred in some cattle with paralytic ileus, volvulus, mesenteric torsion, torsion of the spiral colon, and caecal dilatation. The prevalence of dilated small intestine was significantly greater in cattle with HBS, paralytic ileus, strangulation, intussusception, and obstruction than in those with torsion of the spiral colon and caecal dilatation (*p* < 0.05; for further details and other significant differences, see Table S17).

### 5.3. Intestinal Atony

**Figure 17:**
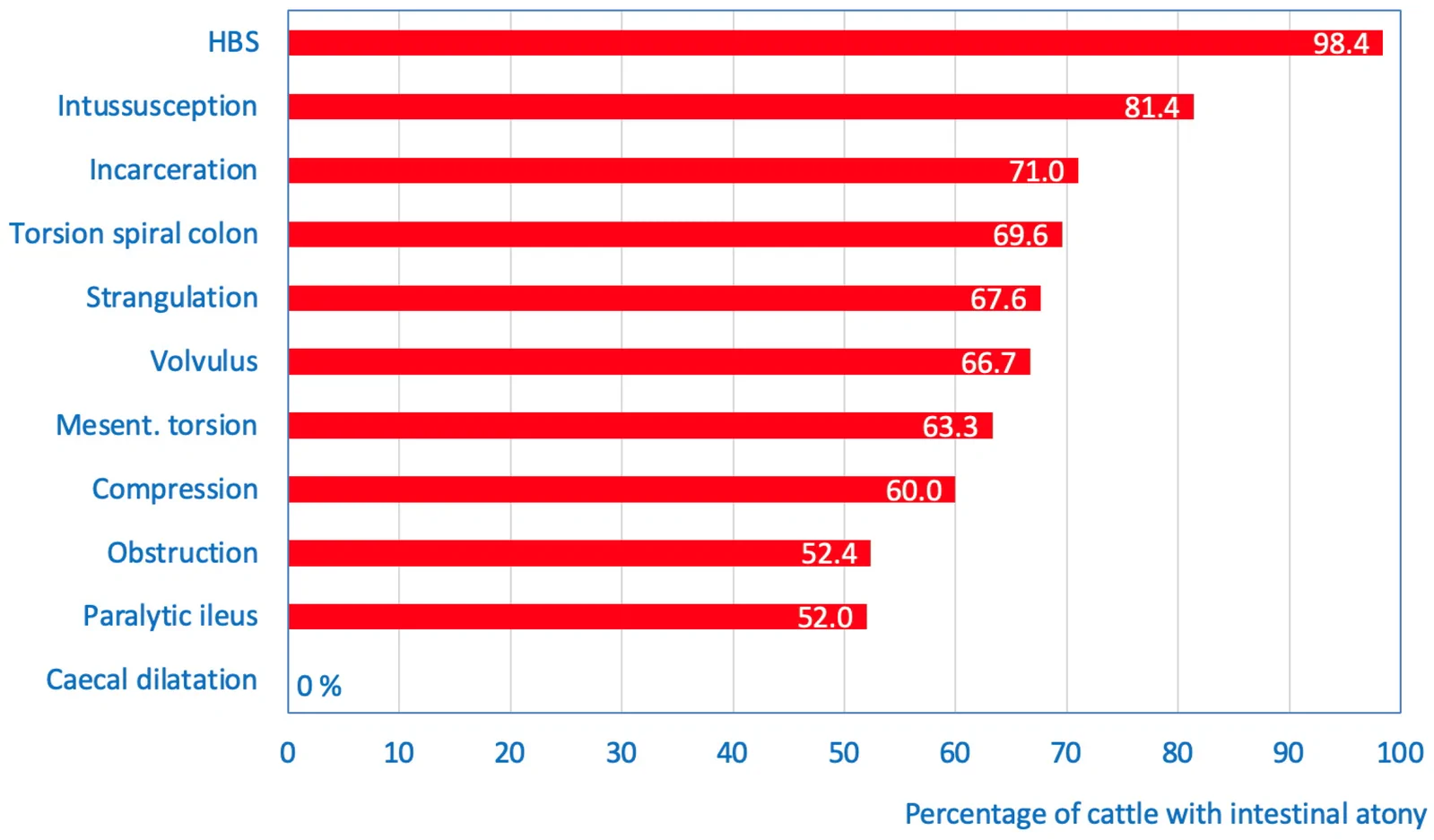
Intestinal atony in 537 cattle with various types of ileus.

Intestinal atony was recorded in 66.5% (357/537) of the cattle and had a prevalence of 98.4% in cattle with HBS to 0% in cattle with caecal dilatation (Figure 17, Table S16). Intestinal atony occurred significantly more often (*p* < 0.05) in cattle with HBS than in cattle with all other types of ileus (except for intussusception) (for further details and other significant differences, see Table S18).

### 5.4. Increased Free Fluid in the Abdomen

**Figure 18:**
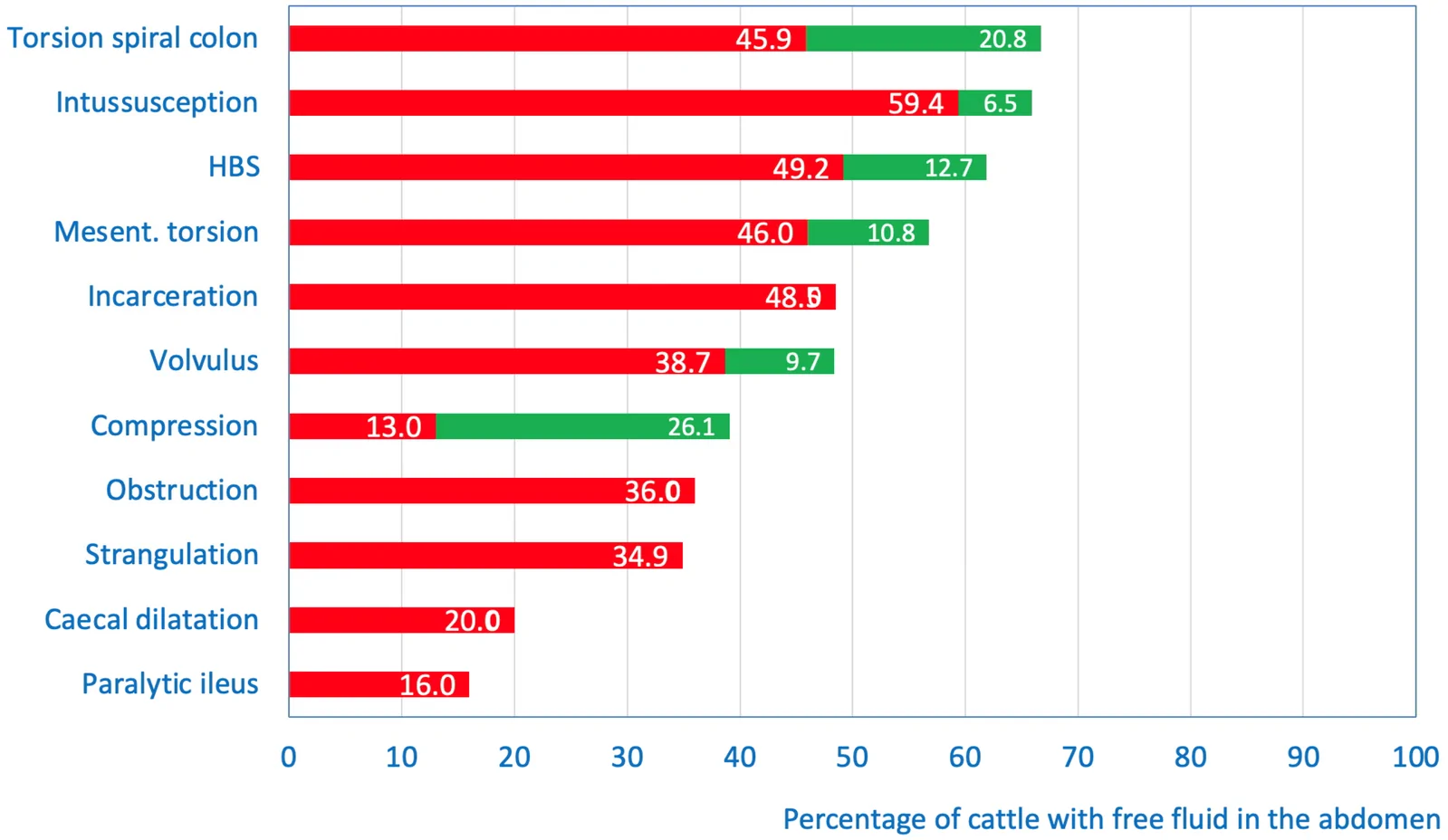
Increased free fluid in the abdomen in 581 cattle with various types of ileus. Red bars = fluid without fibrin; green bars = fluid with fibrin.

Increased free fluid in the abdomen was recorded in 46.4% (275/593) of the cattle and had a prevalence of 66.7% (torsion of the spiral colon) to 16.0% (paralytic ileus) (Figure 18, Table S16). The fluid seen in 275 cattle was free of fibrin (red bars) in 241 cases (87.6%) and contained fibrin (green bars) in the remaining 34 cases (12.4%). Cattle with torsion of the spiral colon had increased free fluid in the abdomen significantly more often (*p* < 0.05) than cattle with caecal dilatation and paralytic ileus (for further details and other significant differences, see Table S19).

### 5.5. Abomasal Dilatation

**Figure 19:**
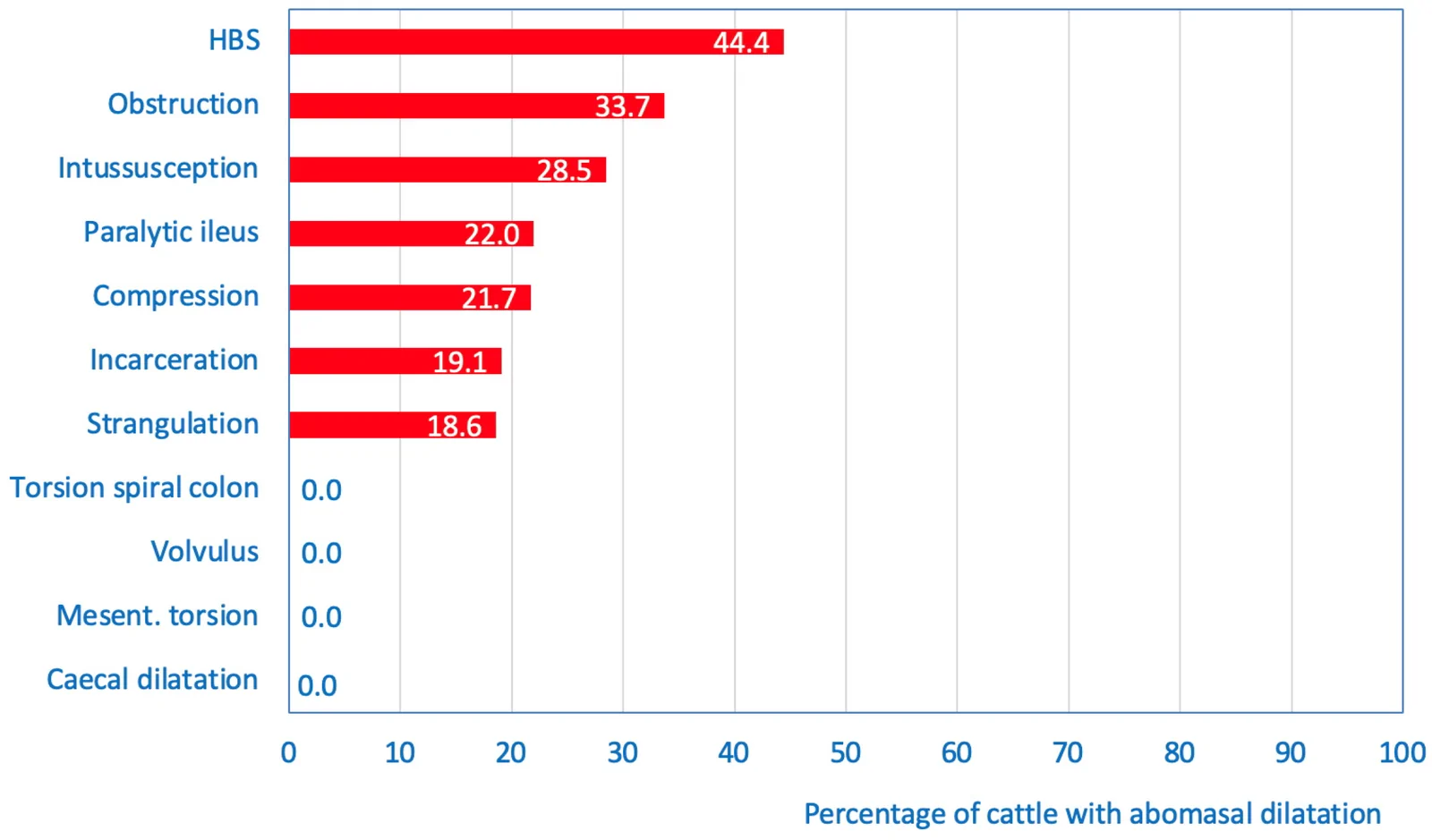
Abomasal dilatation in 582 cattle with various types of ileus.

Abomasal dilatation was recorded in 22.3% (130/582) of the cattle and had a prevalence of 44.4 (HBS) to 0% (torsion of the spiral colon, volvulus, mesenteric torsion, caecal dilatation) (Figure 19, Table S16). Abomasal dilatation was significantly more common (*p* < 0.05) in cattle with HBS and obstruction than in cattle with torsion of the spiral colon, volvulus, mesenteric torsion, and caecal dilatation (for further details and other significant differences, see Table S20).

### 5.6. Empty Poststenotic Small Intestine

**Figure 20:**
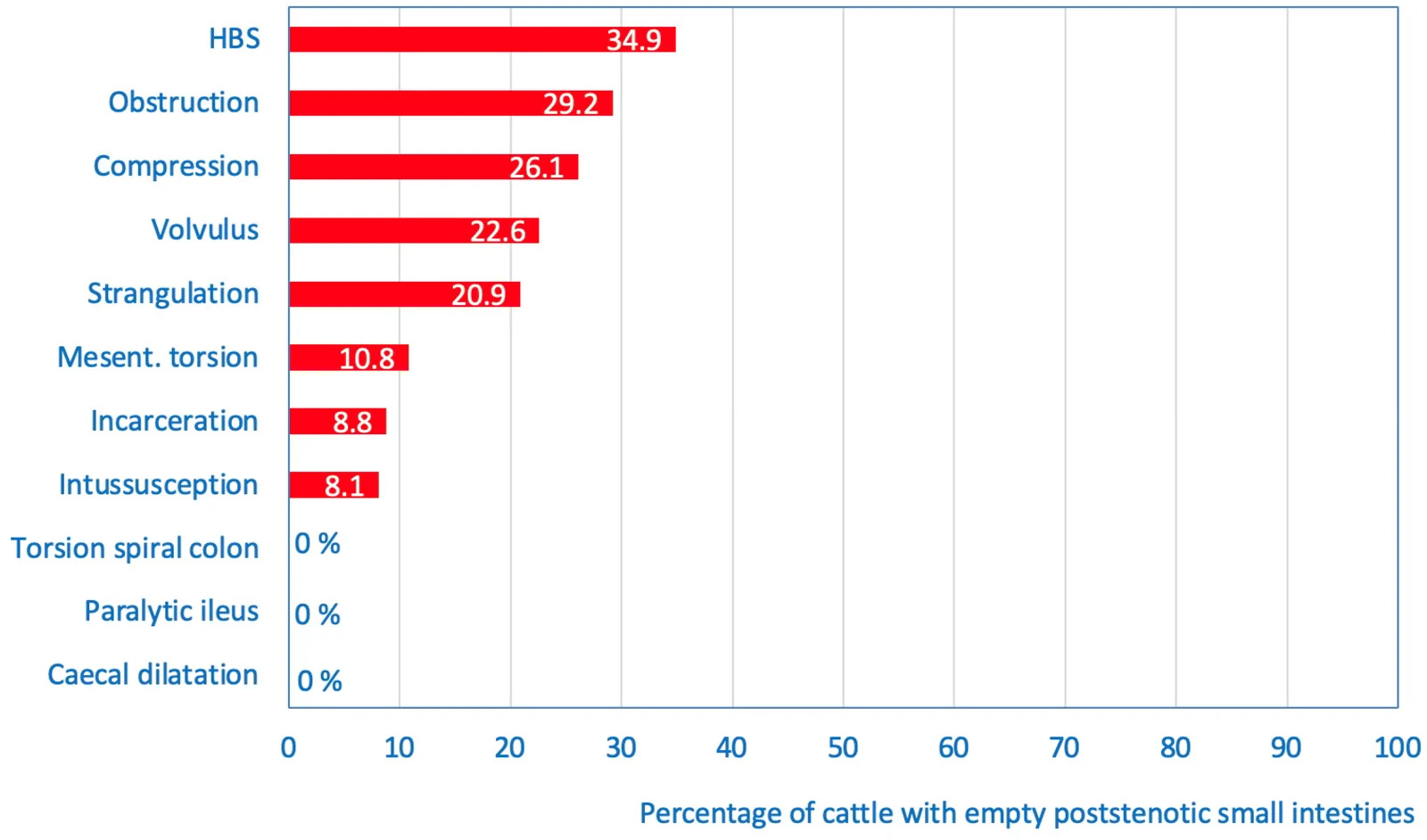
Empty poststenotic small intestine in 582 cattle with various types of ileus.

Empty poststenotic small intestine was recorded in 15.5% (90/582) of the cattle. The prevalence varied from 34.9 (HBS) to 0% (torsion of the spiral colon, paralytic ileus, caecal dilatation) (Figure 20, Table S16). Empty poststenotic small intestinal loops were significantly more common (*p* < 0.05) in cattle with HBS and obstruction than in cattle with intussusception, paralytic ileus, and caecal dilatation (for further details and other significant differences, see Table S21).

### 5.7. Visualisation of the Ileus

The ileus was visible in 12.0% (70/582) of the examined cattle (Table S16). The dilated caecum was visible in all cattle (100%) with caecal dilatation, and the abdominal hernia was verified in 23.6% of cattle with incarceration. A blood clot in the small intestine was identified ultrasonographically in 19.0% of the cattle with HBS, a *bowel-within-bowel* pattern in 5.7% of cattle with intussusception, adhesions between a dilated loop of jejunum and a multi-chambered abscess in 4.3% of cattle with compression, and an obstructive mass in 3.4% of cattle with obstruction.

## 6. Discussion of the Laboratory Findings

### 6.1. Overview

As stated in the analysis of clinical findings in cattle with ileus [1], the same is true for the analysis of blood variables: *an ileus is not an ileus*. Blood values can vary widely among ileus types and even within them. The principal laboratory abnormalities in cattle with ileus are those related to shock (haemoconcentration, praerenal azotaemia, and me-tabolic acidosis) and abomasal reflux syndrome (hypochloraemic hypokalaemic metabolic alkalosis). Other findings important for the therapeutic approach are hypocalcaemia and hypermagnesaemia. The severity of laboratory changes depends mainly on the duration of illness and the anatomic location of the ileus, and thus the degree of reflux. The longer the illness, the more pronounced the changes. It is therefore possible that the time between ileus onset and clinical examination is too short for the electrolyte and blood-gas changes to manifest, which was suggested by other authors [35,36]. The following discussion starts with abomasal reflux syndrome, followed by shock-associated laboratory changes, although the former is probably less significant in ileus than shock.

### 6.2. Abomasal Reflux Syndrome (Hypochloraemic Hypokalaemic Metabolic Alkalosis)

The anatomic location of ileus determines the severity of abomasal reflux syndrome and associated hypochloraemic hypokalaemic metabolic alkalosis; the further cranial the ileus, the more severe the changes [21]. Obstruction of the proximal small intestine results in more rapid and severe changes than distal lesions. This was shown experimentally by blocking the passage of ingesta in the cranial, middle, and caudal parts of the small intestine [21] but also clinically in cattle with duodenal sigmoid flexure volvulus [24] and in spontaneously occurring duodenal, jejunal, and ileal obstruction [10]. The changes are more rapid and severe with duodenal obstruction than with jejunal and ileal obstruction; thus, the increase in rumen chloride concentration depends on the location of the ileus. Hypochloraemia results from impaired chloride ion resorption by the small intestine. This, in turn, leads to decreased secretion of bicarbonate into the small intestine and an increase in blood bicarbonate concentration, resulting in compensated metabolic alkalosis (increased base excess and normal blood pH). Initial compensatory mechanisms for alkalosis include increased renal secretion of bicarbonate, potassium, sodium, and water, alveolar hypoventilation with increased partial pressure of carbon dioxide (pCO_2_) (Figure 15), and a shift of hydrogen ions from the intracellular to the extracellular space.

Hypokalaemia results from renal potassium excretion as well as the movement of potassium from the blood into the intracellular space to compensate for the intracellular loss of hydrogen ions. Hypokalaemia is further exacerbated by a lack of forage intake [37], accompanied by relatively high milk yield in some cows [38]. The increased renal excretion of water causes haemoconcentration and praerenal azotaemia (increased serum urea concentration). Once these compensatory mechanisms are exhausted, the blood pH increases. In summary, the changes described result in hypochloraemic hypokalaemic me-tabolic alkalosis accompanied by dehydration and praerenal azotaemia.

Alkalosis was not a prominent feature in this study except in cattle with HBS, which had a prevalence of 55.6% (blood pH > 7.45). Cows with compression and intussusception were more often alkalotic (43.3 and 41.1%) than cows with left displacement of the abomasum (LDA, 36.0%, 445/1224, ref. [39]. Alkalosis was a rare finding in cows with torsion of the spiral colon (7.7%), caecal dilatation (4.8%), and mesenteric torsion (4.3%). A possible explanation for the high prevalence of alkalosis in cattle with HBS is that these cows tended to have had a longer duration of illness at the time of examination than cows with other ileus types. Intestinal blockage by an intraluminal blood clot, and thus ileus, does not occur at the start of HBS nor in all affected cows. Rather, necrohaemorrhagic jejunitis initially causes intraluminal blood clots and sloughed mucosa that subsequently lead to mechanical bowel obstruction.

### 6.3. Haemoconcentration

Haemoconcentration (haematocrit > 35%) occurred in 44.7% of the cattle and had the highest prevalence in cows with mesenteric torsion, incarceration, obstruction, and torsion of the spiral colon. In our experience, haemoconcentration was primarily an expression of hypovolaemic shock caused by intraluminal fluid sequestration, impaired intestinal fluid absorption, electrolyte imbalances, and pain. Haemoconcentration occurred in only 27.4% of cows with HBS, possibly because intramucosal haematomata are characteristic of this disease [40].

### 6.4. Urea Concentration and Praerenal Azotaemia

In all likelihood, the increased urea concentration was attributable to praerenal azotaemia. Measurement of creatinine and urine specific gravity would have been required to validate this statement; however, urinalysis was not carried out, and creatinine concentration was measured only in cases with increased urea concentration. Whether azotaemia was related to shock or reflux in individual cases was not known. Praerenal azotaemia was the rarest in cows with caecal dilatation and strangulation. Compared with small intestinal ileus, caecal dilatation is a less serious disease; cows with strangulation, mesenteric torsion, and torsion of the spiral colon often had rapid progression of clinical signs and were referred before the manifestation of praerenal azotaemia.

### 6.5. Hypocalcaemia

It is noteworthy that hypocalcaemia, hypermagnesaemia, and hypokalaemia were the most common blood changes in this study. The median calcium concentration was below the reference interval for all 11 ileus types, and cows with paralytic ileus and caecal dilatation had the highest prevalence of hypocalcaemia. Hypocalcaemia may be attributable to anorexia, gastrointestinal stasis and ischaemia-related decrease in gastrointestinal absorption, and possibly milk production [41,42]. It is not known whether hypocalcaemia is a cause or a result of ileus; however, hypocalcaemia is mentioned explicitly as the cause of paralytic ileus [43,44]. Calcium deficiency may cause caecal dilatation, most likely with other factors. On the other hand, intestinal obstruction leads to increased myoelectrical activity of the intestine, resulting in increased calcium consumption [45,46]. Thus, it is conceivable that hypocalcaemia results from caecal dilatation. In cattle with mesenteric torsion, hypocalcaemia occurred less frequently (59.6%), possibly because of the fulminant course of the disease, leaving less time for the development of hypocalcaemia.

### 6.6. Hypermagnesaemia

Hypermagnesaemia is not mentioned as a cardinal finding of ileus in textbooks [19,44,47]. Hypermagnesaemia (magnesium > 1.00 mmol/L) is a common finding in cattle with ileus. Except for cows with intussusception, the median magnesium concentration exceeded the reference interval for all ileus types. Hypermagnesaemia occurred most frequently in cows with HBS, torsion of the spiral colon, and mesenteric torsion. The cause of hypermagnesaemia is thought to be reduced glomerular filtration because of dehydration [48]. This is supported by the observation that serum magnesium and urea concentrations were significantly correlated (r = 0.53).

### 6.7. Decreased Blood pH

The mean prevalence of acidosis based on a blood pH ≤ 7.35 was similar (15.2%) to the prevalence of alkalosis (18.8%). It varied widely from 47.8 to 3.3%. Acidosis was most common in cows with mesenteric torsion and torsion of the spiral colon. An explanation for the occurrence of acidosis is the increase in L-lactate resulting from hypovolaemia, shock, and possibly ischaemia of the intestinal wall with subsequent necrosis [19,42,49]. Unfortunately, L-lactate was measured later in the study and not analysed due to low numbers.

### 6.8. Rumen Chloride

Reflux of abomasal contents into the rumen increases rumen chloride concentration; this was seen in only 16.2% of cases, but large variations occurred among ileus types. It was most common in cows with obstruction and least common in those with caecal dilatation. For comparison, the rumen chloride concentration was increased in 78% of cows with LDA [39], but the threshold was set at 25 mmol/L rather than at 30 mmol/L in the present study. It can be concluded that abomasal reflux is not a major feature in cattle with ileus, unlike cattle with LDA. However, an increase in rumen chloride in a cow with ileus points to a proximal location in the duodenum or jejunum.

### 6.9. Correlations Between the Laboratory Variables

Even though 52 out of 66 correlations between laboratory variables were significant, only 7 had a correlation coefficient r > 0.50 and thus were clinically relevant. The correlation coefficient of 0.80 between blood pH and base excess is systemic because, by definition, the two variables are closely correlated. Compensation mechanisms (see above) are the reason why the coefficient is not higher than 0.80. These mechanisms aim to maintain blood pH within the normal range as long as possible when the base excess increases. The corresponding values can be seen in the vertical yellow area above the intersection with the horizontal yellow area in Figure 14A: the blood pH is between 7.35 and 7.45, and the base excess is higher than +2.0 mmol/L; this means that the alkalosis is compensated. Only after the compensatory mechanisms are exhausted does the blood pH increase, leading to decompensated metabolic alkalosis. The corresponding values appear in the upper right quadrant in Figure 14A: both blood pH and base excess are elevated.

The correlations between urea and inorganic phosphate concentrations and between urea and magnesium concentrations indicate reduced excretion of both electrolytes as azotaemia increases. The negative correlation between base excess and potassium resulted from the intracellular movement of potassium as base excess increased (see above, abomasal reflux syndrome and compensation mechanisms).

### 6.10. Boxplots and Prevalences

Boxplots show all parameters required for the visual scientific assessment of a dataset, including the maximum and minimum values, the upper and lower quartiles, and the median. They also show the distribution, spread, data skewness, and outliers. In addition to boxplots, we presented the frequencies of abnormal laboratory and ultrasonographic findings for each type of ileus. The latter are often more helpful to the clinician when assessing a cow with ileus because they directly illustrate how often a variable is abnormal with different types of ileus. For instance, when a dilated caecum is palpated transrectally in a cow with haemoconcentration, simple caecal dilatation is unlikely because only 23.3% of these cows had an elevated haematocrit. Torsion of the spiral colon would be more likely because caecal dilatation is accompanied by haemoconcentration in 63.8% of these cows. Similar examples exist for other variables; 91.9% of cows with HBS had azotaemia, and therefore, it is unlikely that a cow with a normal urea concentration has HBS. In contrast, hypocalcaemia does not indicate the type of ileus because the proportions of hypocalcaemia did not differ among cattle with different ileus types.

Ideally, the diagnostic sensitivity and specificity, the positive and negative predictive values, and the positive likelihood ratio (LR+) should be provided, as in the studies of the clinical [50] and laboratory [51] findings in cows with traumatic reticuloperitonitis and with different forms of abomasal ulcers. However, this would require a healthy control group, which was not available for the cows with ileus. Without this, the frequency of abnormal variables helps the clinician determine whether the laboratory findings fit the clinical tentative diagnosis.

## 7. Discussion of the Ultrasonographic Findings

### 7.1. Dilated Small and Large Intestines

A dilated small intestine was the most important ultrasonographic finding in ileus, with a mean prevalence of 91.3% in all cattle. It occurred in 87.1 to 100% of all small intestinal ileus types. The proportion of dilated small intestine was smaller only when the large intestine was included (with mesenteric torsion) or directly involved in the ileus (torsion of the spiral colon or caecal dilatation). In addition to the small intestine, the large intestine was also dilated on ultrasonograms of 24.0 to 100% of cattle with paralytic ileus. Torsion of the entire intestinal tract in cows with mesenteric torsion resulted in concurrent dilatation of the small and large intestines. Dilated small intestine in cattle with torsion of the spiral colon and caecal dilatation can be explained by the backflow of ingesta into the small intestine. Paralytic ileus may affect the small and large intestines and, therefore, both may be dilated in affected cattle.

### 7.2. Intestinal Atony

Intestinal atony had a mean prevalence of 66.5% and was the second most common ultrasonographic finding. Except for caecal dilatation, its prevalence ranged from 98.4 to 52.9%; caecal dilatation was the only ileus type not accompanied by small intestinal atony. When associated with small intestinal atony, caecal dilatation is, in all likelihood, combined with a much more severe illness involving the caecum. Examples include torsion of the spiral colon or mesenteric torsion because small intestinal atony is not a typical feature of caecal dilatation. Clinical findings of severe illness should prompt an immediate laparotomy. When the large intestine can be palpated transrectally, impaired small intestinal motility is therefore important for the differentiation of caecal dilatation and torsion of the spiral colon or mesenteric torsion.

### 7.3. Increased Free Fluid in the Abdomen

An increase in free fluid in the abdomen was detected in almost half (46.4%) of the cattle. In most cases, the fluid was hypoechogenic because of transudation from constricted blood vessels. Cattle with mild types of ileus, including caecal dilatation and paralytic ileus, had significantly lower prevalences (20.0 and 16.0%) than cattle with constricted blood vessels from torsion of the spiral colon (66.7%). Fibrin in the fluid indicated that the intestinal wall had probably been damaged, allowing transudation to occur, or that peritonitis was present. Cattle with obstruction, strangulation, caecal dilatation, and paralytic ileus had no fibrin in the abdominal fluid.

### 7.4. Abomasal Dilatation

Abomasal dilatation occurs when the ileus results in backflow of ingesta into the abomasum. The dilated abomasum contains hypoechogenic ingesta, and the abomasal folds are often seen as echogenic, sickle-shaped structures. Abomasal dilatation was rare, occurring in 22.3% of cattle. The highest prevalence was in cattle with HBS but was not observed in cattle with volvulus, mesenteric torsion, torsion of the spiral colon, and caecal dilatation; the fulminant course of these latter ileus types did not allow sufficient time for backflow to occur. Furthermore, spiral colon blockage or caecal dilatation is a substantial distance from the abomasum, requiring considerable time for dilatation to occur.

### 7.5. Empty Poststenotic Small Intestine

An empty poststenotic small intestine was identified in only 15.5% of the cattle. It was most common in cattle with HBS (34.9%) and obstruction (29.2%), both of which are associated with bowel obstruction at a minimum of one site. The prevalence was much smaller for intussusception (8.1%) and incarceration (8.8%) because complete blockage does not occur initially, allowing some ingesta to pass through. Empty poststenotic small intestine was not seen in cows with paralytic ileus, torsion of the spiral colon, and caecal dilatation. In cattle with paralytic ileus, the entire small intestine is usually affected. By definition, the small intestine is not involved in cattle with torsion of the spiral colon and caecal dilatation. This means that these latter conditions, as well as paralytic ileus, can likely be excluded diagnostically when an empty poststenotic small intestine is detected.

### 7.6. Visualisation of the Ileus

The actual cause of the ileus could only be visualised in 12.0% (70/582) of all cattle. This statement is limited because 30 of these cows had caecal dilatation, and 17 had an external herniation; those illnesses can usually be diagnosed by external examination (herniation) or transrectally (caecal dilatation). Ultrasonography was essential for the exact diagnosis in only 23 of the 70 cattle, viz., in 12 with HBS, 7 with intussusception, 3 with obstruction, and 1 with compression.

### 7.7. Limitations

One limitation was that the examinations occurred over 30 years. Throughout this time, the senior author conducted or supervised the sonographic examinations, but the co-authors also supervised many examinations in later years. The same was true for examinations by interns without supervision at night and on weekends; the records were scrutinised, and the cows were re-examined when deemed necessary. Inclusion in the present analysis occurred only when all criteria were fulfilled, and the majority of variables were examined. The senior author reassessed every case.

Another limiting aspect was the technical advances in ultrasound equipment over time, particularly improvements in resolution. The depth of penetration changed little and remained at about 15 to 20 cm. Intestinal dilatation, decreased or absent intestinal motility, strands of fibrin, fluid between intestinal loops, and other findings could be visualised with older machines just as well as with modern machines. However, older scans were of poorer quality than those obtained with modern machines and not as suitable for documentation in publications. More important than the type of machine is that for 30 years the same individual (UB) performed the sonographic examinations, or they were carried out under his or the co-authors’ supervision. Obviously, the interpretation of sonographic findings improved with experience, and it is possible that in later years, details were recognised that may have been missed earlier.

## 8. Conclusions

The laboratory variables are of limited diagnostic value in cows with ileus but are primarily important for the optimal therapeutic correction of fluid, electrolyte, and acid–base abnormalities. In many cases, ultrasonography is well suited for confirming or excluding a tentative diagnosis of ileus. The most important finding is a dilated small intestine, detected in 91.3% of all cattle; however, the cause of ileus could only be visualised in 12.0%. To enhance the diagnostic capabilities of ultrasonography, we recommend that cattle suspected of having ileus be examined transrectally and externally from the ventral abdominal wall. We are convinced that this approach will improve diagnosis and clinical characterisation. Thorough training is crucial for proficient recognition and correct interpretation of abnormal findings.

## Data Availability

The datasets used and analysed for this study are available from the corresponding author on reasonable request.

## Supplementary Materials

The following supporting information can be downloaded at: https://www.mdpi.com/article/10.3390/ani16182850/s1, Table S1: Means and 95% CI of laboratory variables in cows with different types of ileus; Table S2: Prevalence of abnormal laboratory variables in cattle with various types of ileus; Table S3: Haematocrit; Table S3.1: Differences in the mean haematocrit in cows with various types of ileus; Table S3.2: Prevalence of haemoconcentration in cows with various types of ileus; Table S4: Leukocytes; Table S4.1: Differences in the mean total leukocyte count in cows with various types of ileus; Table S4.2: Prevalence of leukocytosis in cows with various types of ileus; Table S5: Total protein concentration; Table S5.1: Differences in the mean total protein concentration in cows with various types of ileus; Table S5.2: Prevalence of hyperproteinaemia in cows with various types of ileus; Table S6: Urea concentration; Table S6.1: Differences in the mean urea concentration in cows with various types of ileus; Table S6.2: Prevalence of azotaemia in cows with various types of ileus; Table S7: Calcium concentration; Table S7.1: Differences in the mean calcium concentration; Table S7.2: Prevalence of hypocalcaemia in cows with various types of ileus; Table S8: Magnesium concentration; Table S8.1: Differences in the mean magnesium concentration; Table S8.2: Prevalence of hypomagnesaemia in cows with various types of ileus; Table S9: Inorganic phosphate concentration; Table S9.1: Differences in the mean inorganic phosphate concentration; Table S9.2: Prevalence of hypophosphataemia in cows with various types of ileus; Table S10: Potassium concentration; Table S10.1: Differences in the mean potassium concentration; Table S10.2: Prevalence of hypokalaemia in cows with various types of ileus; Table S11: Chloride concentration; Table S11.1: Differences in the mean chloride concentration; Table S11.2: Prevalence of hypochloraemia in cows with various types of ileus; Table S12: Blood pH; Table S12.1: Differences in the mean blood pH; Table S12.2: Prevalence of blood pH < 7.35 in cows with various types of ileus; Table S12.3: Prevalence of increased blood pH in cows with various types of ileus; Table S13: Base excess; Table S13.1: Differences in the mean base excess in cows with various types of ileus; Table S13.2: Prevalence of increased base excess in cows with various types of ileus; Table S14: Rumen chloride concentration; Table S14.1: Differences in the mean rumen chloride concentration; Table S14.2: Prevalence of increased rumen chloride concentration in cows with various types of ileus; Table S15: Correlations between the laboratory variables; Table S16: Ultrasonographic findings in cattle with various types of small and large intestinal ileus; Table S17: Dilated small intestine in 574 cattle with various types of ileus; Table S18: Intestinal atony in 537 cattle with various types of ileus; Table S19: Free fluid in the abdomen in 581 cattle with various types of ileus; Table S20: Abomasal dilatation in 582 cattle with various types of ileus; Table S21: Empty poststenotic small intestine in 582 cattle with various types of ileus
