# Supplementary material for: Analysis and Comparison of the Laboratory and Ultrasonographic Findings in Cattle with Various Types of Small and Large Intestinal Ileus
Source: Animals (Basel). 2026 Sep 10;16(18):2850. doi: 10.3390/ani16182850 (PMC13603740; doi:10.3390/ani16182850)
Supplement: Supplementary file 1 [file animals-16-02850-s001.zip › 6C Proofs 3 Supplementary Tables animals-4479533 Supplementary Tables for author proof 2.pdf]

Supplementary Tables

Table S1: Means and 95% CI of laboratory variables in cows with different types of ileus.

| Type of ileus (number of cattle) | Haematocrit (%) | Leukocytes (/µl) | Total protein (g/L) | Urea (mmol/L) | Calcium (mmol/L) | Magnesium (mmol/L) |
|---|---|---|---|---|---|---|
| Caecal dilatation (461) | 33, 32-33, 444 | 10,297; 9,913-10,682, 444 | 79, 78-79, 443 | 6.6, 6.3-6.9, 446 | 2.07, 2.05-2.10, 332 | 1.04, 1.01-1.06, 331 |
| Compression (35) | 36, 34-38, 35 | 9,745; 8,590-10,900, 35 | 86, 83-89, 35 | 10.3, 8.3-12.3, 35 | 2.13, 1.99-2.26, 22 | 1.22, 1.09-1.35, 20 |
| HBS (63) | 32, 31-34, 62 | 10,980; 9,902-12,058, 62 | 82, 79-84, 62 | 13.1, 11.6-14.5, 62 | 1.93, 1.76-2.10, 62 | 1.43, 1.31-1.54, 62 |
| Incarceration (85) | 39, 38-41, 85 | 9,617; 8,614-10,169, 82 | 77, 74-79, 84 | 9.1, 7.8-10.4, 83 | 2.06, 1.95-2.17, 44, | 1.14, 1.07-1.21, 44 |
| Intussusception (126) | 37, 37-38, 126 | 10,519; 9,702-11,336, 124 | 81, 79-83, 126 | 9.7, 8.6-10.7, 124 | 2.10, 1.99-2.22, 51 | 1.24, 1.13-1.35, 51 |
| Mesenteric torsion (61) | 39, 38-41, 60 | 12,120; 10,800-13439, 59 | 75, 73-78, 60 | 8.1, 7.0-9.1, 59 | 2.25, 2.05-2.46, 47 | 1.52, 1.36-1.67, 47 |
| Obstruction (110) | 38, 36-39, 110 | 9,785; 8,996-10574, 110 | 82, 80-84, 110 | 8.9, 8.1-9.8, 110 | 2.10, 1.99-2.20, 78 | 1.22, 1.14-1.29, 77 |
| Paralytic ileus (57) | 36, 34-37, 57 | 9,100; 8,110-10,089, 57 | 78, 75-81, 55 | 6.7, 5.9-7.5, 57 | 1.75, 1.58-1.93, 40 | 1.20, 1.11-1.29, 40 |
| Strangulation (60) | 37, 35-38, 59 | 9,003; 8,195-9,811, 59 | 80, 78-83, 59 | 7.8, 6.1-9.6, 60 | 2.17, 2.03-2.31, 29 | 1.13, 1.00-1.27, 28 |
| Torsion of the spiral col. (58) | 38, 36-40, 58 | 10,939; 9,647-12,232, 58 | 77, 75-80, 57 | 7.8, 6.8-8.9, 58 | 2.08, 1.93-2.24, 24 | 1.26, 1.14-1.37, 24 |
| Volvulus (47) | 37, 35-39, 45 | 10,909; 9,195-12,623, 42 | 76, 72-80, 44 | 9.4, 7.7-11.2, 43 | 2.07, 1.85-2.28, 27 | 1.19, 1.06-1.33, 26 |
| Mean (total) | 35, 35-36, 1141 | 10,266; 10,013 -10,518, 1132 | 79, 79-80, 1135 | 8.1, 7.8-8.4, 1137 | 2.07, 2.03-2.10, 756 | 1.17, 1.14-1.19, 750 |

Continuation of Table S1.

| Type of ileus (number of cattle) | Inorganic phosphate (mmol/L) | Potassium (mmol/L) | Chloride (mmol/L) | pH | Base excess (mmol/L) | Rumen chloride (mmol/L) |
|---|---|---|---|---|---|---|
| Caecal dilatation (461) | 1.51, 1.44-1.57, 330 | 3.89, 3.83-3.96, 443 | 101, 100-101, 445 | 7.39, 7.38-7.39, 421 | -0.2, -0.6 to 0.16, 420 | 20, 19-20, 396 |
| Compression (35) | 1.69, 1.40-1.97, 21 | 3.11, 2.78-3.44, 35 | 89, 85-94, 34 | 7.45, 7.42- 7.47, 30 | 9.5, 6.2-12.9, 30 | 28, 24-32, 34 |
| HBS (63) | 2.03, 1.89-2.18, 62 | 2.74, 2.59-2.89, 62 | 81, 78-83, 62 | 7.45, 7.43-7.47, 54 | 10.1, 8.1-12.1, 54 | 27, 25-30, 55 |
| Incarceration (85) | 1.96, 1.74-2.18, 43 | 3.57, 3.38-3.76, 84 | 94, 92-96, 83 | 7.42, 7.40-7.44, 65 | 5.3, 3.3-7.3, 65 | 26, 23-28, 72 |
| Intussusception (126) | 2.09, 1.87-2.30, 51 | 3.19, 3.08-3.31, 125 | 88, 87-90, 124 | 7.44. 7.42-7.45, 107 | 6.8, 5.4-8.2, 107 | 25, 23-26, 103 |
| Mesenteric torsion (61) | 1.97, 1.69-2.24, 47 | 4.32, 4.03-4.61, 59 | 96, 94-98, 59 | 7.33, 7.30-7.36, 46 | -2.0, -3.8 to -0.3, 46 | 21, 17-25, 34 |
| Obstruction (110) | 1.68, 1.53-1.84, 77 | 3.34, 3.19-3.49, 110 | 92, 89-94, 110 | 7.43, 7.42-7.44, 98 | 6.4, 4.6-8.1, 98 | 31, 27-36, 91 |
| Paralytic ileus (57) | 1.50, 1.30-1.69, 40 | 3.34, 3.16-3.53, 57 | 96, 94-98, 57 | 7.41, 7.39-7.42, 47 | 3.4, 1.7-5.2, 47 | 22, 20-24, 45 |
| Strangulation (60) | 1.66, 1.31-2.00, 28 | 3.77, 3.55-3.99, 60 | 98, 95-101, 60 | 7.41, 7.39-7.43, 47 | 3.6, 1.3-5.9, 47 | 30, 25-35, 52 |
| Torsion of the spiral col. (58) | 1.58, 1.29-1.87, 24 | 4.06, 3.83-4.30, 58 | 99, 97-102, 58 | 7.37, 7.35-7.39, 39 | 0.5, -1.0 to 2.0, 39 | 22, 19-24, 44 |
| Volvulus (47) | 1.87, 1.48-2.26, 27 | 3.57, 3.33-3.82, 42 | 96, 92-99, 43 | 7.38, 7.35-7.41, 34 | 2.5, 0.8-4.9, 34 | 23, 19-27, 37 |
| Mean (total) | 1.69, 1.64-1.74, 750 | 3.64, 3.59-3.67, 1135 | 96, 95-96, 1135 | 7.40, 7.39-7.41, 988 | 2.8, 2.4-3.3, 987 | 23, 23-24, 963 |

Table S2: Prevalence of abnormal laboratory variables in cattle with various types of ileus

| Type of ileus (no. of cattle) | Haematocrit >35% | Leukocytes >10,000/µl | Total protein >80 g/L | Urea >6.5 mmol/L | Calcium <2.30 mmol/L | Magnesium >1.00 mmol/L | Inorg. phosphate <1.30 mmol/L |
|---|---|---|---|---|---|---|---|
| Caecal dilatation (461) | 23.2% (103/444) | 38.7% (172/444) | 39.7% (176/443) | 39.7% (177/446) | 85.2% (283/332) | 50.2% (166/331) | 40.9% (135/330) |
| Compression (35) | 45.7% (16/35) | 42.9% (15/35) | 62.9% (22/35) | 62.9% (22/35) | 77.3% (17/22) | 75.0% (15/20) | 19.0% (4/21) |
| HBS (63) | 27.4% (17/62) | 58.1% (36/62) | 58.1% (36/62) | 91.9% (57/62) | 82.3% (51/62) | 88.7% (55/62) | 11.3% (7/62) |
| Incarceration (85) | 70.6% (60/85) | 36.6% (30/82) | 38.1% (32/84) | 61.4% (51/83) | 81.8% (36/44) | 70.5% (31/44) | 16.3% (7/36) |
| Intussusception (126) | 61.1% (77/126) | 48.4% (60/124) | 51.6% (65/126) | 62.1% (77/124) | 76.5% (39/51) | 43.1% (22/51) | 23.5% (12/51) |
| Mesenteric torsion (61) | 71.7% (43/60) | 61.0% (36/59) | 26.7% (16/60) | 52.5% (31/59) | 59.6% (28/47) | 83.0% (39/47) | 17.0% (8/47) |
| Obstruction (110) | 66.4% (73/110) | 37.3% (41/110) | 50.0% (55/110) | 66.4% (73/110) | 71.8% (56/78) | 75.3% (58/77) | 28.6% (22/77) |
| Paralytic ileus (57) | 40.4% (23/57) | 35.1% (20/57) | 36.4% (20/55) | 42.1% (24/57) | 87.5% (35/40) | 77.5% (31/40) | 37.5% (15/40) |
| Strangulation (60) | 57.6% (34/59) | 27.1% (16/59) | 45.8% (27/59) | 38.3% (23/60) | 69.0% (20/29) | 60.7% (17/28) | 32.1% (9/28) |
| Torsion of the spiral colon (58) | 63.8% (37/58) | 51.7% (30/58) | 33.3% (19/57) | 53.4% (31/58) | 79.2% (19/24) | 87.5% (21/24) | 37.5% (9/24) |
| Volvulus (47) | 60.0% (27/45) | 47.6% (20/42) | 34.1% (15/44) | 62.8% (27/43) | 74.1% (20/27) | 61.5% (16/26) | 33.3% (9/27) |
| Mean (total) | 44.7% (510/1141) | 42.0% (476/1132)) | 42.6% (483/1135) | 52.3% (593/1137) | 79.9% (604/756) | 62.8% (471/750) | 31.6% (237/750) |

Continuation of Table S2.

| Type of ileus (number of cattle) | Potassium <4.0 mmol/L | Chloride <95 mmol/L | pH≤7.35 | pH >7.45 | Base excess <-2.0 mmol/L | Base excess >2.0 mmol/L | Rumen chloride> 30 mmol/L |
|---|---|---|---|---|---|---|---|
| Caecal dilatation (461) | 14.0% (188/443) | 12.4% (55/445) | 14.0% (59/421) | 4.8% (20/421) | 30.5% (128/420) | 26.9% (113/420) | 5.6% (22/396) |
| Compression (35) | 82.9% (29/35) | 64.7% (22/34) | 3.3% (1/30) | 43.3% (13/30) | 10.0% (3/30) | 80.0% (23/30) | 29.4% (10/34) |
| HBS (63) | 98.4% (61/62) | 98.4% (61/62) | 7.4% (4/54) | 55.6% (30/54) | 7.4% (4/54) | 87.0% (47/54) | 20.0% (11/55) |
| Incarceration (85) | 72.6% (61/84) | 43.4% (36/83) | 12.3% (8/65) | 27.7% (18/65) | 9.2% (6/65) | 63.1% (41/65) | 25.0% (18/72) |
| Intussusception (126) | 89.6% (112/125) | 70.2% (87/124) | 8.4% (9/107) | 41.1% (44/107) | 7.5% (8/107) | 72.0% (77/107) | 20.4% (21/103) |
| Mesenteric torsion (61) | 44.1% (26/59) | 39.0% (23/59) | 47.8% (22/46) | 4.3% (2/46) | 45.7% (21/46) | 26.1% (12/46) | 11.8% (4/34) |
| Obstruction (110) | 80.9% (89/110) | 52.7% (58/110) | 13.3% (13/98) | 33.7% (33/98) | 11.2% (11/98) | 63.3% (62/98) | 39.6% (36/91) |
| Paralytic ileus (57) | 89.5% (51/57) | 40.4% (23/57) | 14.9% (7/47) | 19.1% (9/47) | 12.8% (6/47) | 57.4% (27/47) | 13.3% (6/45) |
| Strangulation (60) | 58.3% (35/60) | 28.3% (17/60) | 12.8% (6/47) | 21.3% (10/47) | 19.1% (9/47) | 51.1% (24/47) | 30.8% (16/52) |
| Torsion of the spiral colon(58) | 50.0% (29/58) | 25.9% (15/58) | 35.9% (14/39) | 7.7% (3/39) | 25.6% (10/39) | 46.2% (18/39) | 9.1% (4/44) |
| Volvulus (47) | 73.8% (31/42) | 37.2% (16/43) | 20.6% (7/34) | 11.8% (4/34) | 26.5% (9/34) | 55.9% (19/34) | 19.6% (9/46) |
| Mean (total) | 63.0% (715/1135) | 36.4% (413/1135) | 15.2% (150/988) | 18.8% (186/988) | 21.8% (215/987) | 47.0% (464/987) | 16.3% (157/963) |

Table S3: Haematocrit

Table S3.1: Differences in the mean haematocrit in cows with various types of ileus

| Type of ileus | Caecal dilat. | Compression | HBS | Incarceration | Intussuscept. | Mesenter. tors. | Obstruction | Paralytic ileus | Strangulation | Tors. spiral colon | Volvulus |
|---|---|---|---|---|---|---|---|---|---|---|---|
| Caecal dilatation | NA | - | - | ** | ** | ** | ** | * | ** | ** | ** |
| Compression | - | NA | - | - | - | - | - | - | - | - | - |
| HBS | - | - | NA | ** | ** | ** | ** | - | ** | ** | ** |
| Incarceration | ** | - | ** | NA | - | - | - | * | - | - | - |
| Intussusception | - | - | ** | - | NA | - | - | - | - | - | - |
| Mesent. torsion. | ** | - | ** | - | - | NA | - | * | - | - | - |
| Obstruction | ** | - | ** | - | - | - | NA | - | - | - | - |
| Paralytic ileus | * | - | - | * | - | * | - | NA | - | - | - |
| Strangulation | ** | - | ** | - | - | - | - | - | NA | - | - |
| Tors. spir. colon | ** | - | ** | - | - | - | - | - | - | NA | - |
| Volvulus | ** | - | ** | - | - | - | - | * | ** | - | NA |

* P<0.05, ** P<0.01, - P>0.05, ANOVA and Bonferroni post-hoc test, NA Not applicable.

# Table S3.2: Prevalence of haemoconcentration in cows with various types of ileus (frequency distributions, chi-square, and Bonferroni post-hoc tests)

| | | | Mesenteric torsion | Incarceration | Obstruction | Torsion of the spiral colon | Intussus-ception | Volvulus | Strangulation | Compression | Paralytic ileus | HBS | Caecal dilatation | Total |
|---|---|---|---|---|---|---|---|---|---|---|---|---|---|---|
| Haemoconcentration | Haemoconcentration (Haematocrit >35%) | Count | 43$_{a}$ | 60$_{a}$ | 73$_{a, b}$ | 37$_{a, b}$ | 77$_{a, b}$ | 27$_{a, b}$ | 34$_{a, b}$ | 16$_{a, b, c}$ | 23$_{b, c}$ | 17$_{c}$ | 103$_{c}$ | 510 |
| | | % within | 71.7% | 70.6% | 66.4% | 63.8% | 61.1% | 60.0% | 57.6% | 45.7% | 40.4% | 27.4% | 23.2% | 44.7% |
| | No haemoconcentration (Haematocrit ≤35%) | Count | 17$_{a}$ | 25$_{a}$ | 37$_{a, b}$ | 21$_{a, b}$ | 49$_{a, b}$ | 18$_{a, b}$ | 25$_{a, b}$ | 19$_{a, b, c}$ | 34$_{b, c}$ | 45$_{c}$ | 341$_{c}$ | 631 |
| | | % within | 28.3% | 29.4% | 33.6% | 36.2% | 38.9% | 40.0% | 42.4% | 54.3% | 59.6% | 72.6% | 76.8% | 55.3% |
| Total | | Count | 60 | 85 | 110 | 58 | 126 | 45 | 59 | 35 | 57 | 62 | 444 | 1141 |
| | | % within | 100.0% | 100.0% | 100.0% | 100.0% | 100.0% | 100.0% | 100.0% | 100.0% | 100.0% | 100.0% | 100.0% | 100.0% |

Each subscript letter denotes a subset of categories whose column proportions do not differ significantly from each other at the .05 level.

**Chi-Square Tests**

| | Value | df | Asymptotic Significance (2-sided) |
|---|---|---|---|
| Pearson Chi-Square | 183.098[a] | 10 | <.001 |
| Likelihood Ratio | 189.609 | 10 | <.001 |
| N of Valid Cases | 1141 | | |

[illegible]

Table S4: Leukocytes

Table S4.1: Differences in the mean total leukocyte count in cows with various types of ileus

| Type of ileus | Caecal dilat. | Compression | HBS | Incarceration | Intussuscept. | Mesenter. tors. | Obstruction | Paralytic ileus | Strangulation | Tors. spiral colon | Volvulus |
|---|---|---|---|---|---|---|---|---|---|---|---|
| Caecal dilat.. | NA | - | - | - | - | - | - | - | - | - | - |
| Compression | - | NA | - | - | - | - | - | - | - | - | - |
| HBS | - | - | NA | - | - | - | - | - | - | - | - |
| Incarceration | - | - | - | NA | - | * | - | - | - | - | - |
| Intussuscept. | - | - | - | - | NA | - | - | - | - | - | - |
| Mes. torsion | - | - | - | * | - | NA | * | ** | ** | - | - |
| Obstruction | - | - | - | - | - | * | NA | - | - | - | - |
| Paralytic ileus | - | - | - | - | - | ** | - | NA | - | - | - |
| Strangulation | - | - | - | - | - | ** | - | - | NA | - | - |
| Tors. spir. col. | - | - | - | - | - | - | - | - | - | NA | - |
| Volvulus | - | - | - | - | - | - | - | - | - | - | NA |

* P<0.05, ** P<0.01, - P>0.05, ANOVA and Bonferroni post-hoc test, NA Not applicable.

# Table S4.2: Prevalence of leukocytosis in cows with various types of ileus (frequency distributions, chi-square, and Bonferroni post-hoc tests)

| | | | Mesenteric torsion | HBS | Torsion of the spiral colon | Intussus-ception | Volvulus | Compression | Caecal dilatation | Obstruction | Incarceration | Paralytic ileus | Strangulation | Total |
|---|---|---|---|---|---|---|---|---|---|---|---|---|---|---|
| Leukocytosis | Leukocytosis (Leukocytes>10'000/µl) | Count | $36_{a}$ | $36_{a}$ | $30_{a, b}$ | $60_{a, b}$ | $20_{a, b}$ | $15_{a, b}$ | $172_{a, b}$ | $41_{a, b}$ | $30_{a, b}$ | $20_{a, b}$ | $16_{b}$ | 476 |
| | | % within | 61.0% | 58.1% | 51.7% | 48.4% | 47.6% | 42.9% | 38.7% | 37.3% | 36.6% | 35.1% | 27.1% | 42.0% |
| | Leukocytosis (Leukocytes ≤10'000/µl) | Count | $23_{a}$ | $26_{a}$ | $28_{a, b}$ | $64_{a, b}$ | $22_{a, b}$ | $20_{a, b}$ | $272_{a, b}$ | $69_{a, b}$ | $52_{a, b}$ | $37_{a, b}$ | $43_{b}$ | 656 |
| | | % within | 39.0% | 41.9% | 48.3% | 51.6% | 52.4% | 57.1% | 61.3% | 62.7% | 63.4% | 64.9% | 72.9% | 58.0% |
| Total | | Count | 59 | 62 | 58 | 124 | 42 | 35 | 444 | 110 | 82 | 57 | 59 | 1132 |
| | | % within | 100.0% | 100.0% | 100.0% | 100.0% | 100.0% | 100.0% | 100.0% | 100.0% | 100.0% | 100.0% | 100.0% | 100.0% |

Each subscript letter denotes a subset of categories whose column proportions do not differ significantly from each other at the .05 level.

**Chi-Square Tests**

| | Value | df | Asymptotic Significance (2-sided) |
|---|---|---|---|
| Pearson Chi-Square | 30.615[a] | 10 | <.001 |
| Likelihood Ratio | 30.649 | 10 | <.001 |
| N of Valid Cases | 1132 | | |

[illegible]

Table S5: Total protein concentration

Table S5.1: Differences in the mean total protein concentration in cows with various types of ileus

| Type of ileus | Caecal dilat. | Compression | HBS | Incarceration | Intussuscept. | Mesent. tors. | Obstruction | Paralytic ileus | Strangulation | Tors. spiral colon | Volvulus |
|---|---|---|---|---|---|---|---|---|---|---|---|
| Caecal dilatat. | NA | ** | - | - | - | - | * | - | - | - | - |
| Compression | ** | NA | - | ** | - | ** | - | * | - | ** | ** |
| HBS | - | - | NA | - | - | * | - | - | - | - | - |
| Incarceration | - | ** | - | NA | - | - | ** | - | - | - | - |
| Intussusception | - | - | - | - | NA | * | - | - | - | - | - |
| Mes. torsion | - | ** | * | . | * | NA | ** | - | - | - | - |
| Obstruction | * | - | - | ** | - | ** | NA | - | - | - | * |
| Paralytic ileus | - | * | - | - | - | - | - | NA | - | - | - |
| Strangulation | - | - | - | - | - | - | - | - | NA | - | - |
| Tors. spir. c. | - | ** | - | - | - | - | - | - | - | NA | - |
| Volvulus | - | ** | - | - | - | - | * | - | - | - | NA |

* P<0.05, ** P<0.01, - P>0.05, ANOVA and Bonferroni post-hoc test, NA Not applicable.

# Table S5.2: Prevalence of hyperproteinaemia in cows with various types of ileus (frequency distributions, chi-square, and Bonferroni post-hoc tests)

| | | | Compression | HBS | Intussus-ception | Obstruction | Strangulation | Caecal dilatation | Incarceration | Paralytic ileus | Volvulus | Torsion of the spiral colon | Mesenteric torsion | Total |
|---|---|---|---|---|---|---|---|---|---|---|---|---|---|---|
| Hyperproteinaemia | Hyperproteinaemia (Total proten >80g/L) | Count | $22_{a}$ | $36_{a}$ | $65_{a, b}$ | $55_{a, b}$ | $27_{a, b}$ | $176_{a, b}$ | $32_{a, b}$ | $20_{a, b}$ | $15_{a, b}$ | $19_{a, b}$ | $16_{b}$ | 483 |
| | | % within | 62.9% | 58.1% | 51.6% | 50.0% | 45.8% | 39.7% | 38.1% | 36.4% | 34.1% | 33.3% | 26.7% | 42.6% |
| | No hyperproteinaemia (Total protein ≤80 g/L) | Count | $13_{a}$ | $26_{a}$ | $61_{a, b}$ | $55_{a, b}$ | $32_{a, b}$ | $267_{a, b}$ | $52_{a, b}$ | $35_{a, b}$ | $29_{a, b}$ | $38_{a, b}$ | $44_{b}$ | 652 |
| | | % within | 37.1% | 41.9% | 48.4% | 50.0% | 54.2% | 60.3% | 61.9% | 63.6% | 65.9% | 66.7% | 73.3% | 57.4% |
| Total | | Count | 35 | 62 | 126 | 110 | 59 | 443 | 84 | 55 | 44 | 57 | 60 | 1135 |
| | | % within | 100.0% | 100.0% | 100.0% | 100.0% | 100.0% | 100.0% | 100.0% | 100.0% | 100.0% | 100.0% | 100.0% | 100.0% |

Each subscript letter denotes a subset of categories whose column proportions do not differ significantly from each other at the .05 level.

**Chi-Square Tests**

| | Value | df | Asymptotic Significance (2-sided) |
|---|---|---|---|
| Pearson Chi-Square | 31.411[a] | 10 | <.001 |
| Likelihood Ratio | 31.609 | 10 | <.001 |
| N of Valid Cases | 1135 | | |

Table S6: Urea concentration

Table S6.1: Differences in the mean urea concentration in cows with various types of ileus

| Type of ileus | Caec. dilatation | Compression | HBS | Incarceration | Intussuscept. | Mesent. tors. | Obstruction | Paralytic ileus | Strangulation | Tors. spiral colon | Volvulus |
|---|---|---|---|---|---|---|---|---|---|---|---|
| Caecal dilat. | NA | ** | ** | ** | ** | - | ** | - | - | - | ** |
| Compression | ** | NA | - | - | - | - | - | * | - | - | - |
| HBS | ** | - | NA | ** | ** | ** | ** | ** | ** | ** | ** |
| Incarceration | ** | - | ** | NA | - | - | - | - | - | - | - |
| Intussusception | ** | - | ** | - | NA | - | - | ** | - | - | - |
| Mes. torsion | - | - | ** | - | - | NA | - | - | - | - | - |
| Obstruction | ** | - | ** | - | - | - | NA | - | - | - | - |
| Paralytic ileus | - | * | ** | - | ** | - | - | NA | - | - | - |
| Strangulation | - | - | ** | - | - | - | - | - | NA | - | - |
| Tors. spir. c. | - | - | ** | - | - | - | - | - | - | NA | - |
| Volvulus | ** | - | ** | - | - | - | - | - | - | - | NA |

* P<0.05, ** P<0.01,-P>0.05, ANOVA and Bonferroni post-hoc test, NA Not applicable.

## Table S6.2: Prevalence of azotaemia in cows with various types of ileus (frequency distributions, chi-square, and Bonferroni post-hoc tests)

| | | | HBS | Obstruction | Compression | Volvulus | Intussus-ception | Incarceration | Torsion of the spiral colon | Mesenteric torsion | Paralytic ileus | Caecal dilatation | Strangulation | Total |
|---|---|---|---|---|---|---|---|---|---|---|---|---|---|---|
| Azotaemia | Azotaemia (Urea >6.5 mmol/L) | Count | $57_{a}$ | $73_{b}$ | $22_{b, c, d, e, f}$ | $27_{b, c, d, e, f}$ | $77_{b, e, f}$ | $51_{b, d, f}$ | $31_{b, c, d, e, f}$ | $31_{b, c, d, e, f}$ | $24_{b, c, d, e, f}$ | $177_{c}$ | $23_{c, d, e, f}$ | 593 |
| | | % within | 91.9% | 66.4% | 62.9% | 62.8% | 62.1% | 61.4% | 53.4% | 52.5% | 42.1% | 39.7% | 38.3% | 52.2% |
| | No azotaemia (Urea≤ 6.5 mmol/L) | Count | $5_{a}$ | $37_{b}$ | $13_{b, c, d, e, f}$ | $16_{b, c, d, e, f}$ | $47_{b, e, f}$ | $32_{b, d, f}$ | $27_{b, c, d, e, f}$ | $28_{b, c, d, e, f}$ | $33_{b, c, d, e, f}$ | $269_{c}$ | $37_{c, d, e, f}$ | 544 |
| | | % within | 8.1% | 33.6% | 37.1% | 37.2% | 37.9% | 38.6% | 46.6% | 47.5% | 57.9% | 60.3% | 61.7% | 47.8% |
| Total | | Count | 62 | 110 | 35 | 43 | 124 | 83 | 58 | 59 | 57 | 446 | 60 | 1137 |
| | | % within | 100.0% | 100.0% | 100.0% | 100.0% | 100.0% | 100.0% | 100.0% | 100.0% | 100.0% | 100.0% | 100.0% | 100.0% |

Each subscript letter denotes a subset of categories whose column proportions do not differ significantly from each other at the .05 level.

**Chi-Square Tests**

| | Value | df | Asymptotic Significance (2-sided) |
|---|---|---|---|
| Pearson Chi-Square | 94.288[a] | 10 | <.001 |
| Likelihood Ratio | 102.249 | 10 | <.001 |
| N of Valid Cases | 1137 | | |

Table S7: Calcium concentration

S7.1: Differences in the mean calcium concentration

| Type of ileus | Caec. dilatation | Compression | HBS | Incarceration | Intussuscept. | Mesent. tors. | Obstruction | Paralytic ileus | Strangulation | Tors. spiral colon | Volvulus |
|---|---|---|---|---|---|---|---|---|---|---|---|
| Caecal dilat. | NA | - | - | - | - | - | - | ** | - | - | - |
| Compression | - | NA | - | - | - | - | - | * | - | - | - |
| HBS | - | - | NA | - | - | ** | - | - | - | - | - |
| Incarceration | - | - | - | NA | - | - | - | * | - | - | - |
| Intussuscept. | - | - | - | - | NA | - | - | ** | - | - | - |
| Mes. torsion | - | - | ** | - | - | NA | - | ** | - | - | - |
| Obstruction | - | - | - | - | - | - | NA | ** | - | - | - |
| Paralytic ileus | ** | * | - | * | ** | ** | ** | NA | ** | - | - |
| Strangulation | - | - | - | - | - | - | - | ** | NA | - | - |
| Tors. spir. c. | - | - | - | - | - | - | - | - | - | NA | - |
| Volvulus | - | - | - | - | - | - | - | - | - | - | NA |

* P<0.05, ** P<0.01, - P>0.05, ANOVA and Bonferroni post-hoc test, NA Not applicable.

## Table S7.2: Prevalence of hypocalcaemia in cows with various types of ileus (frequency distributions, chi-square, and Bonferroni post-hoc tests)

| | | | Paralytic ileus | Caecal dilatation | HBS | Incarceration | Torsion of the spiral colon | Compression | Intussus-ception | Volvulus | Obstruction | Strangulation | Mesenteric torsion | Total |
|---|---|---|---|---|---|---|---|---|---|---|---|---|---|---|
| Hypocalcaemia | Hypocalcaemia (Calcium <2.3 mmol/L) | Count | 35$_{a, b}$ | 283$_{b}$ | 51$_{a, b}$ | 36$_{a, b}$ | 19$_{a, b}$ | 17$_{a, b}$ | 39$_{a, b}$ | 20$_{a, b}$ | 56$_{a, b}$ | 20$_{a, b}$ | 28$_{a}$ | 604 |
| | | % within | 87.5% | 85.2% | 82.3% | 81.8% | 79.2% | 77.3% | 76.5% | 74.1% | 71.8% | 69.0% | 59.6% | 79.9% |
| | No hypocalcaemia (Calcium ≥2.3mmol/L) | Count | 5$_{a, b}$ | 49$_{b}$ | 11$_{a, b}$ | 8$_{a, b}$ | 5$_{a, b}$ | 5$_{a, b}$ | 12$_{a, b}$ | 7$_{a, b}$ | 22$_{a, b}$ | 9$_{a, b}$ | 19$_{a}$ | 152 |
| | | % within | 12.5% | 14.8% | 17.7% | 18.2% | 20.8% | 22.7% | 23.5% | 25.9% | 28.2% | 31.0% | 40.4% | 20.1% |
| Total | | Count | 40 | 332 | 62 | 44 | 24 | 22 | 51 | 27 | 78 | 29 | 47 | 756 |
| | | % within | 100.0% | 100.0% | 100.0% | 100.0% | 100.0% | 100.0% | 100.0% | 100.0% | 100.0% | 100.0% | 100.0% | 100.0% |

Each subscript letter denotes a subset of categories whose column proportions do not differ significantly from each other at the .05 level.

**Chi-Square Tests**

| | Value | df | Asymptotic Significance (2-sided) |
|---|---|---|---|
| Pearson Chi-Square | 26.132[a] | 10 | .004 |
| Likelihood Ratio | 24.256 | 10 | .007 |
| N of Valid Cases | 756 | | |

Table S8: Magnesium concentration

Table S8.1: Differences in the mean magnesium concentration

| Type of ileus | Caec. dilatation | Compression | HBS | Incarceration | Intussuscept. | Mesent. tors. | Obstruction | Paralytic ileus | Strangulation | Tors. spiral colon | Volvulus |
|---|---|---|---|---|---|---|---|---|---|---|---|
| Caecal dilat. | NA | - | ** | - | ** | ** | ** | - | - | - | - |
| Compression | - | NA | - | - | - | * | - | - | - | - | - |
| HBS | ** | - | NA | ** | - | - | ** | * | ** | - | - |
| Incarceration | - | - | ** | NA | - | ** | - | - | - | - | - |
| Intussuscept. | ** | - | - | - | NA | ** | - | - | - | - | - |
| Mes. torsion | ** | * | - | ** | ** | NA | ** | ** | ** | - | ** |
| Obstruction | ** | - | ** | - | - | ** | NA | - | - | - | - |
| Paralytic ileus | - | - | * | - | - | ** | - | NA | - | - | - |
| Strangulation | - | - | - | - | - | ** | - | - | NA | - | - |
| Tors. spir. c. | - | - | ** | - | - | - | - | - | - | NA | - |
| Volvulus | - | - | - | - | - | ** | - | - | - | - | NA |

* P<0.05, ** P<0.01, - P>0.05, ANOVA and Bonferroni post-hoc test, NA Not applicable.

# Table S8.2: Prevalence of hypomagnesaemia in cows with various types of ileus (frequency distributions, chi-square, and Bonferroni post-hoc tests)

| | | | HBS | Torsion of the spiral colon | Mesenteric torsion | Paralytic ileus | Obstruction | Compression | Incarceration | Volvulus | Strangulation | Caecal dilatation | Intussus-ception | Total |
|---|---|---|---|---|---|---|---|---|---|---|---|---|---|---|
| Hypermagnesaemia | Hypermagnesaemia (Magnesium >1.00 mmol/L) | Count | $55_{a}$ | $21_{a}$ | $39_{a}$ | $31_{a, b}$ | $58_{a}$ | $15_{a, b}$ | $31_{a, b}$ | $16_{a, b}$ | $17_{a, b}$ | $166_{b}$ | $22_{b}$ | 471 |
| | | % within | 88.7% | 87.5% | 83.0% | 77.5% | 75.3% | 75.0% | 70.5% | 61.5% | 60.7% | 50.2% | 43.1% | 62.8% |
| | No hypermagnesaemia (Magnesium ≤1.00 mmol/L) | Count | $7_{a}$ | $3_{a}$ | $8_{a}$ | $9_{a, b}$ | $19_{a}$ | $5_{a, b}$ | $13_{a, b}$ | $10_{a, b}$ | $11_{a, b}$ | $165_{b}$ | $29_{b}$ | 279 |
| | | % within | 11.3% | 12.5% | 17.0% | 22.5% | 24.7% | 25.0% | 29.5% | 38.5% | 39.3% | 49.8% | 56.9% | 37.2% |
| Total | | Count | 62 | 24 | 47 | 40 | 77 | 20 | 44 | 26 | 28 | 331 | 51 | 750 |
| | | % within | 100.0% | 100.0% | 100.0% | 100.0% | 100.0% | 100.0% | 100.0% | 100.0% | 100.0% | 100.0% | 100.0% | 100.0% |

Each subscript letter denotes a subset of categories whose column proportions do not differ significantly from each other at the .05 level.

**Chi-Square Tests**

| | Value | df | Asymptotic Significance (2-sided) |
|---|---|---|---|
| Pearson Chi-Square | 74.703[a] | 10 | <.001 |
| Likelihood Ratio | 79.962 | 10 | <.001 |
| N of Valid Cases | 750 | | |

Table S9: Inorganic phosphate concentration

Table S9.1: Differences in the mean inorganic phosphate concentration

| Type of ileus | Caec. dilatation | Compression | HBS | Incarceration | Intussuscept. | Mesent. tors. | Obstruction | Paralytic ileus | Strangulation | Tors. spiral colon | Volvulus |
|---|---|---|---|---|---|---|---|---|---|---|---|
| Caecal dilat. | NA | - | ** | ** | ** | ** | - | - | - | - | - |
| Compression | - | NA | - | - | - | - | - | - | - | - | - |
| HBS | ** | - | NA | - | - | - | - | ** | - | - | - |
| Incarceration | ** | - | - | NA | - | - | - | - | - | - | - |
| Intussuscept. | ** | - | - | - | NA | - | - | ** | - | - | - |
| Mes. torsion | ** | - | - | - | - | NA | - | - | - | - | - |
| Obstruction | - | - | - | - | - | - | NA | - | - | - | - |
| Paralytic ileus | - | - | ** | - | ** | - | - | NA | - | - | - |
| Strangulation | - | - | - | - | - | - | - | - | NA | - | - |
| Tors. spir. c. | - | - | - | - | - | - | - | - | - | NA | - |
| Volvulus | - | - | - | - | - | - | - | - | - | - | NA |

* P<0.05, ** P<0.01, - P>0.05, ANOVA and Bonferroni post-hoc test, NA Not applicable.

## Table S9.2: Prevalence of hypophosphataemia in cows with various types of ileus (frequency distributions, chi-square, and Bonferroni post-hoc tests)

| | | | Caecal dilatation | Paralytic ileus | Torsion of the spiral colon | Volvulus | Strangulation | Obstruction | Intussus-ception | Compression | Mesenteric torsion | Incarceration | HBS | Total |
|---|---|---|---|---|---|---|---|---|---|---|---|---|---|---|
| Hypophosphataemia | Hypophosphataemia (Inorg. phosphate <1.30 mmol/L) | Count | $135_{a}$ | $15_{a, b}$ | $9_{a, b}$ | $9_{a, b}$ | $9_{a, b}$ | $22_{a, b}$ | $12_{a, b}$ | $4_{a, b}$ | $8_{a, b}$ | $7_{a, b}$ | $7_{b}$ | 237 |
| | | % within | 40.9% | 37.5% | 37.5% | 33.3% | 32.1% | 28.6% | 23.5% | 19.0% | 17.0% | 16.3% | 11.3% | 31.6% |
| | No hypophosphataemia (Inorg. phosphate ≥1.30 mmol/L) | Count | $195_{a}$ | $25_{a, b}$ | $15_{a, b}$ | $18_{a, b}$ | $19_{a, b}$ | $55_{a, b}$ | $39_{a, b}$ | $17_{a, b}$ | $39_{a, b}$ | $36_{a, b}$ | $55_{b}$ | 513 |
| | | % within | 59.1% | 62.5% | 62.5% | 66.7% | 67.9% | 71.4% | 76.5% | 81.0% | 83.0% | 83.7% | 88.7% | 68.4% |
| Total | | Count | 330 | 40 | 24 | 27 | 28 | 77 | 51 | 21 | 47 | 43 | 62 | 750 |
| | | % within | 100.0% | 100.0% | 100.0% | 100.0% | 100.0% | 100.0% | 100.0% | 100.0% | 100.0% | 100.0% | 100.0% | 100.0% |

Each subscript letter denotes a subset of categories whose column proportions do not differ significantly from each other at the .05 level.

**Chi-Square Tests**

| | Value | df | Asymptotic Significance (2-sided) |
|---|---|---|---|
| Pearson Chi-Square | 38.821[a] | 10 | <.001 |
| Likelihood Ratio | 41.961 | 10 | <.001 |
| N of Valid Cases | 750 | | |

Table S10: Potassium concentration

Table S10.1: Differences in the mean potassium concentration

| Type of ileus | Caec. dilatation | Compression | HBS | Incarceration | Intussuscept. | Mesent. tors. | Obstruction | Paralytic ileus | Strangulation | Tors. spiral colon | Volvulus |
|---|---|---|---|---|---|---|---|---|---|---|---|
| Caecal dilat. | NA | ** | ** | - | ** | ** | ** | ** | - | - | - |
| Compression | ** | NA | - | - | - | ** | - | - | ** | ** | - |
| HBS | ** | - | NA | ** | ** | ** | ** | ** | ** | ** | ** |
| Incarceration | * | - | ** | NA | * | ** | - | - | - | ** | - |
| Intussuscept. | ** | - | ** | * | NA | ** | - | - | ** | ** | - |
| Mes. torsion | ** | ** | ** | ** | ** | NA | ** | ** | ** | - | ** |
| Obstruction | ** | - | ** | - | - | ** | NA | - | * | ** | - |
| Paralytic ileus | ** | - | ** | - | - | ** | - | NA | - | ** | - |
| Strangulation | - | ** | ** | - | ** | ** | * | - | NA | - | - |
| Tors. spir. c. | - | ** | ** | * | ** | - | ** | ** | - | NA | - |
| Volvulus | - | - | ** | - | - | ** | - | - | - | - | NA |

* P<0.05, ** P<0.01, - P>0.05, ANOVA and Bonferroni post-hoc test, NA Not applicable.

# Table S10.2: Prevalence of hypokalaemia in cows with various types of ileus (frequency distributions, chi-square, and Bonferroni post-hoc tests)

| | | | HBS | Intussus-ception | Paralytic ileus | Compression | Obstruction | Volvulus | Incarceration | Strangulation | Torsion of the spiral colon | Mesenteric torsion | Caecal dilatation | Total |
|---|---|---|---|---|---|---|---|---|---|---|---|---|---|---|
| Hypokalaemia | Hypokalaemia (Potassium <4.0 mmol/L) | Count | $61_{a}$ | $112_{a, b}$ | $51_{a, b}$ | $29_{a, b, c, d}$ | $89_{a, b, d}$ | $31_{b, c, d, e}$ | $61_{b, c, d}$ | $35_{c, d, e, f}$ | $32_{c, e, f}$ | $26_{e, f}$ | $188_{f}$ | 715 |
| | | % within | 98.4% | 89.6% | 89.5% | 82.9% | 80.9% | 73.8% | 72.6% | 58.3% | 55.2% | 44.1% | 42.4% | 63.0% |
| | No hypokalaemia (Potassium ≥4.0 mmol/L) | Count | $1_{a}$ | $13_{a, b}$ | $6_{a, b}$ | $6_{a, b, c, d}$ | $21_{a, b, d}$ | $11_{b, c, d, e}$ | $23_{b, c, d}$ | $25_{c, d, e, f}$ | $26_{c, e, f}$ | $33_{e, f}$ | $255_{f}$ | 420 |
| | | % within | 1.6% | 10.4% | 10.5% | 17.1% | 19.1% | 26.2% | 27.4% | 41.7% | 44.8% | 55.9% | 57.6% | 37.0% |
| Total | | Count | 62 | 125 | 57 | 35 | 110 | 42 | 84 | 60 | 58 | 59 | 443 | 1135 |
| | | % within | 100.0% | 100.0% | 100.0% | 100.0% | 100.0% | 100.0% | 100.0% | 100.0% | 100.0% | 100.0% | 100.0% | 100.0% |

Each subscript letter denotes a subset of categories whose column proportions do not differ significantly from each other at the .05 level.

**Chi-Square Tests**

| | Value | df | Asymptotic Significance (2-sided) |
|---|---|---|---|
| Pearson Chi-Square | 206.383[a] | 10 | <.001 |
| Likelihood Ratio | 231.387 | 10 | <.001 |
| N of Valid Cases | 1135 | | |

[illegible]

Table S11: Chloride concentration

Table S11.1: Differences in the mean chloride concentration

| Type of ileus | Caec. dilatation | Compression | HBS | Incarceration | Intussuscept. | Mesent. tors. | Obstruction | Paralytic ileus | Strangulation | Tors. spiral colon | Volvulus |
|---|---|---|---|---|---|---|---|---|---|---|---|
| Caecal dilat. | NA | ** | ** | ** | ** | ** | ** | * | - | - | * |
| Compression | ** | NA | ** | - | - | * | - | * | ** | ** | - |
| HBS | ** | ** | NA | ** | ** | ** | ** | ** | ** | ** | ** |
| Incarceration | ** | - | ** | NA | ** | - | - | - | - | - | - |
| Intussuscept. | ** | - | ** | ** | NA | ** | - | ** | ** | ** | ** |
| Mes. torsion | ** | * | ** | - | ** | NA | - | - | - | - | - |
| Obstruction | ** | - | ** | - | - | - | NA | - | ** | ** | - |
| Paralytic ileus | * | * | ** | - | ** | - | - | NA | - | - | - |
| Strangulation | - | ** | ** | - | ** | - | ** | - | NA | - | - |
| Tors. spir. c. | - | ** | ** | - | ** | - | ** | - | - | NA | - |
| Volvulus | * | - | ** | - | ** | - | - | - | - | - | NA |

* P<0.05, ** P<0.01, - P>0.05, ANOVA and Bonferroni post-hoc test, NA Not applicable.

Table S11.2: Prevalence of hypochloraemia in cows with various types of ileus (frequency distributions, chi-square, and Bonferroni post-hoc tests)

| | | | HBS | Intussus-ception | Compression | Obstruction | Incarceration | Paralytic ileus | Mesenteric torsion | Volvulus | Strangulation | Torsion of the spiral colon | Caecal dilatation | Total |
|---|---|---|---|---|---|---|---|---|---|---|---|---|---|---|
| Hypochloraemia | Hypochloraemia (Serum chloride <95 mmol/L) | Count | $61_{a}$ | $87_{b}$ | $22_{b, c}$ | $58_{b, c, d}$ | $36_{c, d, e}$ | $23_{c, d, e}$ | $23_{c, d, e}$ | $16_{c, d, e}$ | $17_{d, e}$ | $15_{e, f}$ | $55_{f}$ | 413 |
| | | % within | 98.4% | 70.2% | 64.7% | 52.7% | 43.4% | 40.4% | 39.0% | 37.2% | 28.3% | 25.9% | 12.4% | 36.4% |
| | No hypochloraemia (Serum chloride ≥95 mmol/L) | Count | $1_{a}$ | $37_{b}$ | $12_{b, c}$ | $52_{b, c, d}$ | $47_{c, d, e}$ | $34_{c, d, e}$ | $36_{c, d, e}$ | $27_{c, d, e}$ | $43_{d, e}$ | $43_{e, f}$ | $390_{f}$ | 722 |
| | | % within | 1.6% | 29.8% | 35.3% | 47.3% | 56.6% | 59.6% | 61.0% | 62.8% | 71.7% | 74.1% | 87.6% | 63.6% |
| Total | | Count | 62 | 124 | 34 | 110 | 83 | 57 | 59 | 43 | 60 | 58 | 445 | 1135 |
| | | % within | 100.0% | 100.0% | 100.0% | 100.0% | 100.0% | 100.0% | 100.0% | 100.0% | 100.0% | 100.0% | 100.0% | 100.0% |

Each subscript letter denotes a subset of categories whose column proportions do not differ significantly from each other at the .05 level.

**Chi-Square Tests**

| | Value | df | Asymptotic Significance (2-sided) |
|---|---|---|---|
| Pearson Chi-Square | 306.307[a] | 10 | <.001 |
| Likelihood Ratio | 333.671 | 10 | <.001 |
| N of Valid Cases | 1135 | | |

Table S12: Blood pH

Table S12.1: Differences in the mean blood pH

| Type of ileus | Caec. dilatation | Compression | HBS | Incarceration | Intussuscept. | Mesent. tors. | Obstruction | Paralytic ileus | Strangulation | Tors. spiral colon | Volvulus |
|---|---|---|---|---|---|---|---|---|---|---|---|
| Caecal dilat. | NA | ** | ** | * | ** | ** | ** | - | - | - | - |
| Compression | ** | NA | - | - | - | ** | - | - | - | ** | ** |
| HBS | ** | - | NA | - | - | ** | - | * | - | ** | ** |
| Incarceration | * | - | - | NA | - | ** | - | - | - | * | - |
| Intussuscept. | ** | - | - | - | NA | ** | - | - | - | ** | ** |
| Mes. torsion | ** | ** | ** | ** | ** | NA | ** | ** | ** | - | ** |
| Obstruction | ** | - | - | - | - | ** | NA | - | - | ** | * |
| Paralytic ileus | - | - | * | - | - | ** | - | NA | - | - | - |
| Strangulation | - | - | - | - | - | ** | - | - | NA | - | - |
| Tors. spir. c. | - | ** | ** | ** | - | - | ** | - | - | NA | - |
| Volvulus | - | ** | ** | - | ** | ** | * | - | - | - | NA |

* P<0.05, ** P<0.01, - P>0.05, ANOVA and Bonferroni post-hoc test, NA Not applicable.

## Table S12.2: Prevalence of decreased blood pH in cows with various types of ileus (frequency distributions, chi-square, and Bonferroni post-hoc tests)

| | | | Mesenteric torsion | Torsion of the spiral colon | Volvulus | Paralytic ileus | Caecal dilatation | Obstruction | Strangulation | Incarceration | Intussus-ception | HBS | Compression | Total |
|---|---|---|---|---|---|---|---|---|---|---|---|---|---|---|
| Blood pH | Blood pH <7.35 | Count | 22$_{a}$ | 14$_{a, b}$ | 7$_{a, b, c}$ | 7$_{b, c}$ | 59$_{c}$ | 13$_{b, c}$ | 6$_{b, c}$ | 8$_{b, c}$ | 9$_{c}$ | 4$_{c}$ | 1$_{b, c}$ | 150 |
| | | % within | 47.8% | 35.9% | 20.6% | 14.9% | 14.0% | 13.3% | 12.8% | 12.3% | 8.4% | 7.4% | 3.3% | 15.2% |
| | Blood pH ≥7.35 | Count | 24$_{a}$ | 25$_{a, b}$ | 27$_{a, b, c}$ | 40$_{b, c}$ | 362$_{c}$ | 85$_{b, c}$ | 41$_{b, c}$ | 57$_{b, c}$ | 98$_{c}$ | 50$_{c}$ | 29$_{b, c}$ | 838 |
| | | % within | 52.2% | 64.1% | 79.4% | 85.1% | 86.0% | 86.7% | 87.2% | 87.7% | 91.6% | 92.6% | 96.7% | 84.8% |
| Total | | Count | 46 | 39 | 34 | 47 | 421 | 98 | 47 | 65 | 107 | 54 | 30 | 988 |
| | | % within | 100.0% | 100.0% | 100.0% | 100.0% | 100.0% | 100.0% | 100.0% | 100.0% | 100.0% | 100.0% | 100.0% | 100.0% |

Each subscript letter denotes a subset of categories whose column proportions do not differ significantly from each other at the .05 level.

**Chi-Square Tests**

| | Value | df | Asymptotic Significance (2-sided) |
|---|---|---|---|
| Pearson Chi-Square | 62.808[a] | 10 | <.001 |
| Likelihood Ratio | 51.383 | 10 | <.001 |
| N of Valid Cases | 988 | | |

## Table S12.3: Prevalence of increased blood pH in cows with various types of ileus (frequency distributions, chi-square, and Bonferroni post-hoc tests)

| | | | HBS | Compression | Intussus-ception | Obstruction | Incarceration | Strangulation | Paralytic ileus | Volvulus | Torsion of the spiral colon | Caecal dilatation | Mesenteric torsion | Total |
|---|---|---|---|---|---|---|---|---|---|---|---|---|---|---|
| Alkalosis | Alkalosis (pH >7.45) | Count | 30$_{a}$ | 13$_{a, b}$ | 44$_{a, b}$ | 33$_{a, b, c}$ | 18$_{a, b, c, d}$ | 10$_{b, c, d}$ | 9$_{b, c, d}$ | 4$_{b, c, d, e}$ | 3$_{c, d, e}$ | 20$_{e}$ | 2$_{d, e}$ | 186 |
| | | % within | 55.6% | 43.3% | 41.1% | 33.7% | 27.7% | 21.3% | 19.1% | 11.8% | 7.7% | 4.8% | 4.3% | 18.8% |
| | No alkalosis (pH≤7.45) | Count | 24$_{a}$ | 17$_{a, b}$ | 63$_{a, b}$ | 65$_{a, b, c}$ | 47$_{a, b, c, d}$ | 37$_{b, c, d}$ | 38$_{b, c, d}$ | 30$_{b, c, d, e}$ | 36$_{c, d, e}$ | 401$_{e}$ | 44$_{d, e}$ | 802 |
| | | % within | 44.4% | 56.7% | 58.9% | 66.3% | 72.3% | 78.7% | 80.9% | 88.2% | 92.3% | 95.2% | 95.7% | 81.2% |
| Total | | Count | 54 | 30 | 107 | 98 | 65 | 47 | 47 | 34 | 39 | 421 | 46 | 988 |
| | | % within | 100.0% | 100.0% | 100.0% | 100.0% | 100.0% | 100.0% | 100.0% | 100.0% | 100.0% | 100.0% | 100.0% | 100.0% |

Each subscript letter denotes a subset of categories whose column proportions do not differ significantly from each other at the .05 level.

**Chi-Square Tests**

| | Value | df | Asymptotic Significance (2-sided) |
|---|---|---|---|
| Pearson Chi-Square | 177.098[a] | 10 | <.001 |
| Likelihood Ratio | 175.956 | 10 | <.001 |
| N of Valid Cases | 988 | | |

Table S13: Base excess

Table S13.1: Differences in the mean base excess in cows with various types of ileus

| Type of ileus | Caec. dilatation | Compression | HBS | Incarceration | Intussuscept. | Mesent. tors. | Obstruction | Paralytic ileus | Strangulation | Tors. spiral colon | Volvulus |
|---|---|---|---|---|---|---|---|---|---|---|---|
| Caecal dilat. | NA | ** | ** | ** | ** | - | ** | ** | ** | - | - |
| Compression | ** | NA | - | - | - | ** | - | ** | ** | ** | ** |
| HBS | ** | - | NA | ** | - | ** | * | ** | ** | ** | ** |
| Incarceration | ** | - | ** | NA | - | ** | - | - | - | ** | - |
| Intussusception | ** | - | - | - | NA | ** | - | - | - | ** | * |
| Mes. torsion | - | ** | ** | ** | ** | NA | ** | ** | ** | - | - |
| Obstruction | ** | - | * | - | - | ** | NA | - | - | ** | - |
| Paralytic ileus | ** | ** | ** | - | - | ** | - | NA | - | - | - |
| Strangulation | ** | ** | ** | - | - | ** | - | - | NA | - | - |
| Tors. spir. c. | - | ** | ** | ** | ** | - | ** | - | - | NA | - |
| Volvulus | - | ** | ** | - | * | - | - | - | - | - | NA |

* P<0.05, ** P<0.01, - P>0.05, ANOVA and Bonferroni post-hoc test, NA Not applicable.

## Table S13.2: Prevalence of increased base excess in cows with various types of ileus (frequency distributions, chi-square, and Bonferroni post-hoc tests)

| | | | HBS | Compression | Intussus-ception | Obstruction | Incarceration | Paralytic ileus | Volvulus | Strangulation | Torsion of the spiral colon | Caecal dilatation | Mesenteric torsion | Total |
|---|---|---|---|---|---|---|---|---|---|---|---|---|---|---|
| Increased base excess | Base excess increased (Base excess >+2.00 mmol/L) | Count | $47_{a}$ | $24_{a, b}$ | $77_{a, b}$ | $62_{a, b}$ | $41_{a, b}$ | $27_{b, c}$ | $19_{a, b, c}$ | $24_{b, c}$ | $18_{b, c, d}$ | $113_{d}$ | $12_{c, d}$ | 464 |
| | | % within | 87.0% | 80.0% | 72.0% | 63.3% | 63.1% | 57.4% | 55.9% | 51.1% | 46.2% | 26.9% | 26.1% | 47.0% |
| | Base excess not increased (Base excess≤+2.00 mmol/L) | Count | $7_{a}$ | $6_{a, b}$ | $30_{a, b}$ | $36_{a, b}$ | $24_{a, b}$ | $20_{b, c}$ | $15_{a, b, c}$ | $23_{b, c}$ | $21_{b, c, d}$ | $307_{d}$ | $34_{c, d}$ | 523 |
| | | % within | 13.0% | 20.0% | 28.0% | 36.7% | 36.9% | 42.6% | 44.1% | 48.9% | 53.8% | 73.1% | 73.9% | 53.0% |
| Total | | Count | 54 | 30 | 107 | 98 | 65 | 47 | 34 | 47 | 39 | 420 | 46 | 987 |
| | | % within | 100.0% | 100.0% | 100.0% | 100.0% | 100.0% | 100.0% | 100.0% | 100.0% | 100.0% | 100.0% | 100.0% | 100.0% |

Each subscript letter denotes a subset of categories whose column proportions do not differ significantly from each other at the .05 level.

**Chi-Square Tests**

| | Value | df | Asymptotic Significance (2-sided) |
|---|---|---|---|
| Pearson Chi-Square | 171.401[a] | 10 | <.001 |
| Likelihood Ratio | 179.927 | 10 | <.001 |
| N of Valid Cases | 987 | | |

Table S14: Rumen chloride concentration

Table S14.1: Differences in the mean rumen chloride concentration

| Type of ileus | Caec. dilatation | Compression | HBS | Incarceration | Intussuscept. | Mesent. tors. | Obstruction | Paralytic ileus | Strangulation | Tors. spiral colon | Volvulus |
|---|---|---|---|---|---|---|---|---|---|---|---|
| Caecal dilat. | NA | ** | ** | ** | ** | - | ** | - | ** | - | - |
| Compression | ** | NA | - | - | - | - | - | - | - | - | - |
| HBS | ** | - | NA | - | - | - | - | - | - | - | - |
| Incarceration | ** | - | - | NA | - | - | - | - | - | - | - |
| Intussuscept. | ** | - | - | - | NA | - | ** | - | - | - | - |
| Mes. torsion | - | - | - | - | - | NA | ** | - | * | - | - |
| Obstruction | ** | - | - | - | ** | ** | NA | ** | - | ** | * |
| Paralytic ileus | - | - | - | - | - | - | ** | NA | - | - | - |
| Strangulation | ** | - | - | - | - | * | - | * | NA | * | - |
| Tors. spir. c. | - | - | - | - | - | - | ** | - | * | NA | - |
| Volvulus | - | - | - | - | - | - | * | - | - | - | NA |

* P<0.05, ** P<0.01, - P>0.05, ANOVA and Bonferroni post-hoc test, NA Not applicable.

Table S14.2: Prevalence of increased rumen chloride concentration in cows with various types of ileus (frequency distributions, chi-square, and Bonferroni post-hoc tests)

| | | | Obstruction | Strangulation | Compression | Incarceration | Intussus-ception | HBS | Volvulus | Paralytic ileus | Mesenteric torsion | Torsion of the spiral colon | Caecal dilatation | Total |
|---|---|---|---|---|---|---|---|---|---|---|---|---|---|---|
| Rumen chloride increased | Rumen chloride increased (>30 mmol/L) | Count | $36_{a}$ | $16_{a, b}$ | $10_{a, b}$ | $18_{a, b}$ | $21_{a, b}$ | $11_{a, b}$ | $9_{a, b}$ | $6_{a, b, c}$ | $4_{a, b, c}$ | $4_{b, c}$ | $22_{c}$ | 157 |
| | | % within | 39.6% | 30.8% | 29.4% | 25.0% | 20.4% | 20.0% | 19.6% | 13.3% | 11.8% | 9.1% | 5.6% | 16.2% |
| | Rumen chloride not increased (≤30 mmol/L) | Count | $55_{a}$ | $36_{a, b}$ | $24_{a, b}$ | $54_{a, b}$ | $82_{a, b}$ | $44_{a, b}$ | $37_{a, b}$ | $39_{a, b, c}$ | $30_{a, b, c}$ | $40_{b, c}$ | $374_{c}$ | 815 |
| | | % within | 60.4% | 69.2% | 70.6% | 75.0% | 79.6% | 80.0% | 80.4% | 86.7% | 88.2% | 90.9% | 94.4% | 83.8% |
| Total | | Count | 91 | 52 | 34 | 72 | 103 | 55 | 46 | 45 | 34 | 44 | 396 | 972 |
| | | % within | 100.0% | 100.0% | 100.0% | 100.0% | 100.0% | 100.0% | 100.0% | 100.0% | 100.0% | 100.0% | 100.0% | 100.0% |

Each subscript letter denotes a subset of categories whose column proportions do not differ significantly from each other at the .05 level.

**Chi-Square Tests**

| | Value | df | Asymptotic Significance (2-sided) |
|---|---|---|---|
| Pearson Chi-Square | 91.158[a] | 10 | <.001 |
| Likelihood Ratio | 89.676 | 10 | <.001 |
| N of Valid Cases | 972 | | |

## Table S15: Correlations between the laboratory variables

| | | Haematocrit | Total protein | Leukocytes | Urea | Calcium | Magnesium | Phosphate | Potassium | Chloride | pH | BE | Rumen chloride |
|---|---|---|---|---|---|---|---|---|---|---|---|---|---|
| Haematocrit | Pearson Correlation | 1.00 | .19** | .04 | .35** | .02 | .35** | .35** | −.06* | −.28** | −.10** | .06* | .23** |
| | Sig. (1−tailed) | | <.001 | .101 | <.001 | .326 | <.001 | <.001 | .026 | <.001 | <.001 | .030 | <.001 |
| | N | 1141 | 1134 | 1131 | 1133 | 752 | 746 | 746 | 1131 | 1131 | 985 | 984 | 960 |
| Total protein | Pearson Correlation | .19** | 1.00 | .06* | .28** | .10** | .24** | .13** | −.06* | −.29** | .08** | .15** | .23** |
| | Sig. (1−tailed) | <.001 | | .019 | <.001 | .003 | <.001 | <.001 | .024 | <.001 | .007 | <.001 | <.001 |
| | N | 1134 | 1135 | 1127 | 1128 | 750 | 744 | 744 | 1126 | 1125 | 981 | 980 | 958 |
| Leukocytes | Pearson Correlation | .04 | .06* | 1.00 | .03 | .06* | .01 | .00 | −.01 | −.07* | −.01 | .04 | −.02 |
| | Sig. (1−tailed) | .101 | .019 | | .126 | .050 | .379 | .455 | .359 | .012 | .428 | .094 | .299 |
| | N | 1131 | 1127 | 1132 | 1130 | 752 | 746 | 746 | 1128 | 1127 | 977 | 976 | 955 |
| Urea | Pearson Correlation | .35** | .28** | .03 | 1.00 | .07* | .53** | .59** | −.21** | −.63** | .11** | .33** | .36** |
| | Sig. (1−tailed) | <.001 | <.001 | .126 | | .035 | <.001 | <.001 | <.001 | <.001 | <.001 | <.001 | <.001 |
| | N | 1133 | 1128 | 1130 | 1137 | 756 | 750 | 750 | 1133 | 1133 | 982 | 981 | 960 |
| Calcium | Pearson Correlation | .02 | .10** | .06* | .07* | 1.00 | .12** | .09** | .13** | −.02 | −.09** | −.09** | .10** |
| | Sig. (1−tailed) | .326 | .003 | .050 | .035 | | <.001 | .007 | <.001 | .288 | .007 | .007 | .008 |
| | N | 752 | 750 | 752 | 756 | 756 | 749 | 750 | 753 | 755 | 691 | 691 | 621 |
| Magnesium | Pearson Correlation | .35** | .24** | .01 | .53** | .12** | 1.00 | .37** | −.04 | −.46** | −.08* | .15** | .19** |
| | Sig. (1−tailed) | <.001 | <.001 | .379 | <.001 | <.001 | | <.001 | .142 | <.001 | .014 | <.001 | <.001 |
| | N | 746 | 744 | 746 | 750 | 749 | 750 | 748 | 747 | 749 | 686 | 686 | 615 |
| Phosphate | Pearson Correlation | .35** | .13** | .00 | .59** | .09** | .37** | 1.00 | .04 | −.45** | −.10** | .11** | .31** |
| | Sig. (1−tailed) | <.001 | <.001 | .455 | <.001 | .007 | <.001 | | .155 | <.001 | .006 | .001 | <.001 |
| | N | 746 | 744 | 746 | 750 | 750 | 748 | 750 | 747 | 749 | 686 | 686 | 615 |
| Potassium | Pearson Correlation | −.06* | −.06* | −.01 | −.21** | .13** | −.04 | .04 | 1.00 | .47** | −.51** | −.57** | −.17** |
| | Sig. (1−tailed) | .026 | .024 | .359 | <.001 | <.001 | .142 | .155 | | <.001 | <.001 | <.001 | <.001 |
| | N | 1131 | 1126 | 1128 | 1133 | 753 | 747 | 747 | 1135 | 1131 | 980 | 979 | 957 |
| Chloride | Pearson Correlation | −.28** | −.29** | −.07* | −.63** | −.02 | −.46** | −.45** | .47** | 1.00 | −.41** | −.66** | −.36** |
| | Sig. (1−tailed) | <.001 | <.001 | .012 | <.001 | .288 | <.001 | <.001 | <.001 | | <.001 | <.001 | <.001 |
| | N | 1131 | 1125 | 1127 | 1133 | 755 | 749 | 749 | 1131 | 1135 | 980 | 979 | 958 |
| pH | Pearson Correlation | −.10** | .08** | −.01 | .11** | −.09** | −.08* | −.10** | −.51** | −.41** | 1.00 | .80** | .14** |
| | Sig. (1−tailed) | <.001 | .007 | .428 | <.001 | .007 | .014 | .006 | <.001 | <.001 | | <.001 | <.001 |
| | N | 985 | 981 | 977 | 982 | 691 | 686 | 686 | 980 | 980 | 988 | 987 | 837 |
| BE | Pearson Correlation | .06* | .15** | .04 | .33** | −.09** | .15** | .11** | −.57** | −.66** | .80** | 1.00 | .28** |
| | Sig. (1−tailed) | .030 | <.001 | .094 | <.001 | .007 | <.001 | .001 | <.001 | <.001 | <.001 | | <.001 |
| | N | 984 | 980 | 976 | 981 | 691 | 686 | 686 | 979 | 979 | 987 | 987 | 836 |
| Rumen chloride | Pearson Correlation | .23** | .23** | −.02 | .36** | .10** | .19** | .31** | −.17** | −.36** | .14** | .28** | 1.00 |
| | Sig. (1−tailed) | <.001 | <.001 | .299 | <.001 | .008 | <.001 | <.001 | <.001 | <.001 | <.001 | <.001 | |
| | N | 960 | 958 | 955 | 960 | 621 | 615 | 615 | 957 | 958 | 837 | 836 | 963 |

**. Correlation is significant at the 0.01 level (1−tailed).

*. Correlation is significant at the 0.05 level (1−tailed).

Table S16: Ultrasonographic findings in cattle with various types of small and large intestinal ileus

| Type of ileus (number of cattle | Small intestine dilated | Large intestine dilated | Intestinal atony | Free fluid in the ab- domen | Abomasal dilatation | Empty poststen. small int. | Cause of the ileus visible | Special findings |
|---|---|---|---|---|---|---|---|---|
| Caecal dilatation (30) | 43.3% (13/30) | 100% (30/30)[1] | 0% (0/30) | 20.0% (6/30)[2] | 0% | 0% | 100% (30/30) | |
| Compression (n=35) | 91.3% (21/23) | NA | 60.0% (12/20) | 39.1% (9/23)[3] | 21.7% (5/23) | 26.1% (6/23) | 4.3% (1/23)[4] | |
| HBS (63) | 100% (63/63) | NA | 98.4% (62/63) | 61.9% (39/63)[5] | 44.4% (28/63) | 34.9% (22/63) | 19.0% (12/63)[6] | |
| Incarceration (85) | 92.6% (63/68) | NA | 71.0% (44/62) | 48.5% (33/68) | 19.1% (13/68) | 8.8% (6/68) | 23.6% (17/68)[7] | |
| Intussusception (126) | 95.9% (118/123) | NA | 81.4% (92/113) | 65.9% (81/123)[8] | 28.5% (35/123) | 8.1% (10/123) | 5.7% (7/123)[9] | |
| Mesenteric torsion (61) | 83.8% (31/37) | 45.9% (17/37)[10] | 63.3% (19/30) | 56.8% (21/37)[11] | 0% | 10.8% (4/37) | 0% | [12] |
| Obstruction (110) | 94.3% (83/88) | NA | 52.4% (43/82) | 36% (32/89)[13] | 33.7% (30/89) | 29.2% (26/89) | 3.4%(3/89)[14] | |
| Paralytic ileus (57) | 100% (50/50) | 24.0% (12/50) | 52.0% (26/50) | 16.0% (8/50)[15] | 22.0% (11/50) | 0% | 0% | |
| Strangulation (60) | 100% (43/43) | NA | 67.6% (25/37) | 34.9% (15/43)[16] | 18.6% (8/43) | 20.9% (9/43) | 0% | |
| Torsion of the spiral colon (58) | 66.7% (12/18) | 68.0% (17/25)[17] | 69.6% (16/23) | 66.7% (16/24)[18] | 0% | 0% | 0% | [19] |
| Volvulus (47) | 87.1% (27/31) | 3.7% (1/27)[20] | 66.7% (18/27) | 48.4% (15/31)[21] | 0% | 22.6% (7/31) | 0% | |
| Mean (total) | 91.3% (524/574) | NA[22] | 66.5% (357/537) | 46.4% (275/593) | 22.3% (130/582) | 15.5% (90/582) | 12.0% (70/582) | |

[1] The wall of the dilated caecum and the proximal ansa of the colon were visible in all cows
[2] Fluid without fibrin (6)
[3] Fluid without fibrin (3), fluid with fibrin (6)
[4] Adhesions between dilated jejunum and chambered abdominal abscess
[5] Fluid without fibrin (31), fluid with fibrin (8)
[6] Blood clots
[7] The incarceration (small intestine) was visible in 17 of 19 cattle with abdominal hernias that were scanned
[8] Fluid without fibrin (73), fluid with fibrin (8)
[9] Intussusception visible
[10] Spiral colon dilated (12), caecum dilated (5)
[11] Fluid without fibrin (17), fluid with fibrin (4)
[12] Ultrasonographic localisation of the ileus in the small intestine (18), in the caecum (6), in the spiral colon (4), in the small and large intestines (7), no information (2)
[13] Fluid without fibrin (32)
[14] Obstructive mass visible
[15] Fluid without fibrin (8)
[16] Fluid without fibrin (15)
[17] Spiral colon dilated (44.0%, 11/25), caecum dilated (24.0%, 6/25)
[18] Fluid without fibrin (11), fluid with fibrin (5)
[19] Ultrasonographic localisation of the ileus in the caecum (40.0%, 10/25), in the spiral colon (12.0%, 3/25), in the small and large intestines (20.0%, 5/25), in the small intestine (16.0%, 4/25), no information (12.0%, 3/25)
[20] In one cow, the spiral colon was dilated
[21] Fluid without fibrin (12), fluid with fibrin (3)
[22] Not analysed, because this finding was limited to 5 ileus types

Table S17: Dilated small intestine in 574 cattle with various types of ileus
(frequency distributions, chi-square, and Bonferroni post-hoc tests)

| | | | HBS | Paralytic ileus | Strangulation | Intussus-ception | Obstruction | Incarceration | Compression | Volvulus | Mesenteric torsion | Torsion of the spiral colon | Caecal dilatation | Total |
|---|---|---|---|---|---|---|---|---|---|---|---|---|---|---|
| Small intestines dilated | Small intestines dilated | Count | $63_{a}$ | $50_{a}$ | $43_{a}$ | $118_{a}$ | $83_{a}$ | $63_{a,b}$ | $21_{a,b}$ | $27_{a,b}$ | $31_{a,b}$ | $12_{b,c}$ | $13_{c}$ | 524 |
| | | % within | 100.0% | 100.0% | 100.0% | 95.9% | 94.3% | 92.6% | 91.3% | 87.1% | 83.8% | 66.7% | 43.3% | 91.3% |
| | Small intestines not dilated | Count | $0_{a}$ | $0_{a}$ | $0_{a}$ | $5_{a}$ | $5_{a}$ | $5_{a,b}$ | $2_{a,b}$ | $4_{a,b}$ | $6_{a,b}$ | $6_{b,c}$ | $17_{c}$ | 50 |
| | | % within | 0.0% | 0.0% | 0.0% | 4.1% | 5.7% | 7.4% | 8.7% | 12.9% | 16.2% | 33.3% | 56.7% | 8.7% |
| Total | | Count | 63 | 50 | 43 | 123 | 88 | 68 | 23 | 31 | 37 | 18 | 30 | 574 |
| | | % within | 100.0% | 100.0% | 100.0% | 100.0% | 100.0% | 100.0% | 100.0% | 100.0% | 100.0% | 100.0% | 100.0% | 100.0% |

Each subscript letter denotes a subset of categories whose column proportions do not differ significantly from each other at the .05 level.

**Chi-Square Tests**

| | Value | df | Asymptotic Significance (2-sided) |
|---|---|---|---|
| Pearson Chi-Square | 123.188[a] | 10 | <.001 |
| Likelihood Ratio | 89.439 | 10 | <.001 |
| N of Valid Cases | 574 | | |

Table S18: Intestinal atony in 537 cattle with various types of ileus
(frequency distributions, chi-square, and Bonferroni post-hoc tests)

| | | | HBS | Intussus-ception | Incarceration | Torsion of the spiral colon | Strangulation | Volvulus | Mesenteric torsion | Compression | Obstruction | Paralytic ileus | Caecal dilatation | Total |
|---|---|---|---|---|---|---|---|---|---|---|---|---|---|---|
| Intestinal atony | Intestinal atony | Count | $62_{a}$ | $92_{a,b}$ | $44_{b,c}$ | $16_{b,c}$ | $25_{b,c}$ | $18_{b,c}$ | $19_{b,c}$ | $12_{b,c}$ | $43_{c}$ | $26_{c}$ | $0_{d}$ | 357 |
| | | % within | 98.4% | 81.4% | 71.0% | 69.6% | 67.6% | 66.7% | 63.3% | 60.0% | 52.4% | 52.0% | 0.0% | 66.5% |
| | No intestinal atony | Count | $1_{a}$ | $21_{a,b}$ | $18_{b,c}$ | $7_{b,c}$ | $12_{b,c}$ | $9_{b,c}$ | $11_{b,c}$ | $8_{b,c}$ | $39_{c}$ | $24_{c}$ | $30_{d}$ | 180 |
| | | % within | 1.6% | 18.6% | 29.0% | 30.4% | 32.4% | 33.3% | 36.7% | 40.0% | 47.6% | 48.0% | 100.0% | 33.5% |
| Total | | Count | 63 | 113 | 62 | 23 | 37 | 27 | 30 | 20 | 82 | 50 | 30 | 537 |
| | | % within | 100.0% | 100.0% | 100.0% | 100.0% | 100.0% | 100.0% | 100.0% | 100.0% | 100.0% | 100.0% | 100.0% | 100.0% |

Each subscript letter denotes a subset of categories whose column proportions do not differ significantly from each other at the .05 level.

**Chi-Square Tests**

| | Value | df | Asymptotic Significance (2-sided) |
|---|---|---|---|
| Pearson Chi-Square | 112.788[a] | 10 | <.001 |
| Likelihood Ratio | 133.180 | 10 | <.001 |
| N of Valid Cases | 537 | | |

Table S19: Free fluid in the abdomen in 581 cattle with various types of ileus (frequency distributions, chi-square, and Bonferroni post-hoc tests)

| | | | Torsion of the spiral colon | Intussus-ception | HBS | Mesenteric torsion | Incarceration | Volvulus | Compression | Obstruction | Strangulation | Caecal dilatation | Paralytic ileus | Total |
|---|---|---|---|---|---|---|---|---|---|---|---|---|---|---|
| Free fluid in the abdomen | Free fluid in the abdomen | Count | $16_{a, b}$ | $81_{b}$ | $39_{a, b}$ | $21_{a, b, c}$ | $33_{a, b, c}$ | $15_{a, b, c, d}$ | $9_{a, b, c, d}$ | $32_{a, c, d}$ | $15_{a, c, d}$ | $6_{c, d}$ | $8_{d}$ | 275 |
| | | % within | 66.7% | 65.9% | 61.9% | 56.8% | 48.5% | 48.4% | 39.1% | 36.0% | 34.9% | 20.0% | 16.0% | 47.3% |
| | No free fluid in the abdomen | Count | $8_{a, b}$ | $42_{b}$ | $24_{a, b}$ | $16_{a, b, c}$ | $35_{a, b, c}$ | $16_{a, b, c, d}$ | $14_{a, b, c, d}$ | $57_{a, c, d}$ | $28_{a, c, d}$ | $24_{c, d}$ | $42_{d}$ | 306 |
| | | % within | 33.3% | 34.1% | 38.1% | 43.2% | 51.5% | 51.6% | 60.9% | 64.0% | 65.1% | 80.0% | 84.0% | 52.7% |
| Total | | Count | 24 | 123 | 63 | 37 | 68 | 31 | 23 | 89 | 43 | 30 | 50 | 581 |
| | | % within | 100.0% | 100.0% | 100.0% | 100.0% | 100.0% | 100.0% | 100.0% | 100.0% | 100.0% | 100.0% | 100.0% | 100.0% |

Each subscript letter denotes a subset of categories whose column proportions do not differ significantly from each other at the .05 level.

**Chi-Square Tests**

| | Value | df | Asymptotic Significance (2-sided) |
|---|---|---|---|
| Pearson Chi-Square | $63.858^{a}$ | 10 | <.001 |
| Likelihood Ratio | 67.139 | 10 | <.001 |
| N of Valid Cases | 581 | | |

Table S20: Abomasal dilatation in 582 cattle with various types of ileus (frequency distributions, chi-square, and Bonferroni post-hoc tests)

| | | | HBS | Obstruction | Intussus-ception | Paralytic ileus | Compression | Incarceration | Strangulation | Torsion of the spiral colon | Volvulus | Mesenteric torsion | Caecal dilatation | Total |
|---|---|---|---|---|---|---|---|---|---|---|---|---|---|---|
| Abomasal dilatation | Abomasal dilatation | Count | $28_{a}$ | $30_{a}$ | $35_{a, b}$ | $11_{a, b, c}$ | $5_{a, b, c}$ | $13_{a, b, c}$ | $8_{a, b, c}$ | $0_{b, c}$ | $0_{c}$ | $0_{c}$ | $0_{c}$ | 130 |
| | | % within | 44.4% | 33.7% | 28.5% | 22.0% | 21.7% | 19.1% | 18.6% | 0.0% | 0.0% | 0.0% | 0.0% | 22.3% |
| | No abomasal dilatation | Count | $35_{a}$ | $59_{a}$ | $88_{a, b}$ | $39_{a, b, c}$ | $18_{a, b, c}$ | $55_{a, b, c}$ | $35_{a, b, c}$ | $25_{b, c}$ | $31_{c}$ | $37_{c}$ | $30_{c}$ | 452 |
| | | % within | 55.6% | 66.3% | 71.5% | 78.0% | 78.3% | 80.9% | 81.4% | 100.0% | 100.0% | 100.0% | 100.0% | 77.7% |
| Total | | Count | 63 | 89 | 123 | 50 | 23 | 68 | 43 | 25 | 31 | 37 | 30 | 582 |
| | | % within | 100.0% | 100.0% | 100.0% | 100.0% | 100.0% | 100.0% | 100.0% | 100.0% | 100.0% | 100.0% | 100.0% | 100.0% |

Each subscript letter denotes a subset of categories whose column proportions do not differ significantly from each other at the .05 level.

**Chi-Square Tests**

| | Value | df | Asymptotic Significance (2-sided) |
|---|---|---|---|
| Pearson Chi-Square | $63.173^{a}$ | 10 | <.001 |
| Likelihood Ratio | 86.568 | 10 | <.001 |
| N of Valid Cases | 582 | | |

Table S21: Empty poststenotic small intestine in 582 cattle with various types of ileus (frequency distributions, chi-square, and Bonferroni post-hoc tests)

| | | | HBS | Obstruction | Compression | Volvulus | Strangulation | Mesenteric torsion | Incarceration | Intussus-ception | Torsion of the spiral colon | Paralytic ileus | Caecal dilatation | Total |
|---|---|---|---|---|---|---|---|---|---|---|---|---|---|---|
| Empty poststenotic small intestines | Empty poststenotic small intestines | Count | $22_{a}$ | $26_{a, b}$ | $6_{a, b, c}$ | $7_{a, b, c}$ | $9_{a, b, c}$ | $4_{a, b, c, d}$ | $6_{b, c, d}$ | $10_{c, d}$ | $0_{b, c, d}$ | $0_{d}$ | $0_{c, d}$ | 90 |
| | | % within | 34.9% | 29.2% | 26.1% | 22.6% | 20.9% | 10.8% | 8.8% | 8.1% | 0.0% | 0.0% | 0.0% | 15.5% |
| | No empty poststenotic small intestines | Count | $41_{a}$ | $63_{a, b}$ | $17_{a, b, c}$ | $24_{a, b, c}$ | $34_{a, b, c}$ | $33_{a, b, c, d}$ | $62_{b, c, d}$ | $113_{c, d}$ | $25_{b, c, d}$ | $50_{d}$ | $30_{c, d}$ | 492 |
| | | % within | 65.1% | 70.8% | 73.9% | 77.4% | 79.1% | 89.2% | 91.2% | 91.9% | 100.0% | 100.0% | 100.0% | 84.5% |
| Total | | Count | 63 | 89 | 23 | 31 | 43 | 37 | 68 | 123 | 25 | 50 | 30 | 582 |
| | | % within | 100.0% | 100.0% | 100.0% | 100.0% | 100.0% | 100.0% | 100.0% | 100.0% | 100.0% | 100.0% | 100.0% | 100.0% |

Each subscript letter denotes a subset of categories whose column proportions do not differ significantly from each other at the .05 level.

**Chi-Square Tests**

| | Value | df | Asymptotic Significance (2-sided) |
|---|---|---|---|
| Pearson Chi-Square | $62.459^{a}$ | 10 | <.001 |
| Likelihood Ratio | 73.333 | 10 | <.001 |
| N of Valid Cases | 582 | | |
